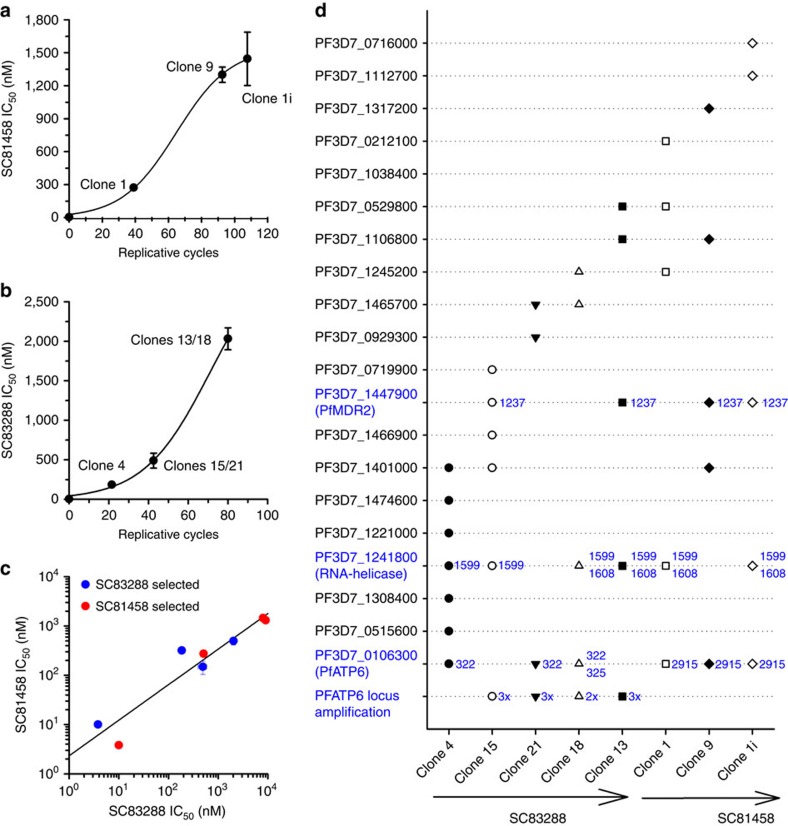# Erratum: SC83288 is a clinical development candidate for the treatment of severe malaria

**DOI:** 10.1038/ncomms15273

**Published:** 2017-04-06

**Authors:** Stefano Pegoraro, Maëlle Duffey, Thomas D. Otto, Yulin Wang, Roman Rösemann, Roland Baumgartner, Stefanie K. Fehler, Leonardo Lucantoni, Vicky M. Avery, Alicia Moreno-Sabater, Dominique Mazier, Henri J. Vial, Stefan Strobl, Cecilia P. Sanchez, Michael Lanzer

Nature Communications 8 Article number:14193 ; DOI: 10.1038/ncomms14193 (2017); Published 01 31 2017; Updated 04 06 2017

This Article contains errors in Figs. 1 and 8 that were introduced during the production process. The compound on the lower right side of Fig. 1 is labelled incorrectly and should be labelled ‘SC83288'. The correct version of Fig. 1 appears below as Fig. 1. In Fig. 8c, ‘SC83458 selected' should read ‘SC81458 selected' and in Fig. 8d ‘SC81758' should read ‘SC81458'. The correct version of Fig. 8 appears below as Fig. 2.

## Figures and Tables

**Figure 1:**
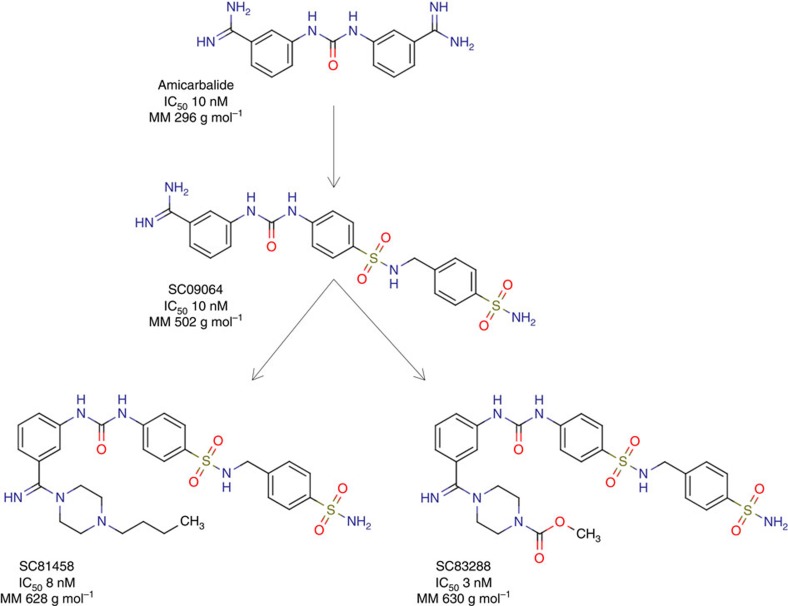


**Figure 2:**