# Supplementary material for: SC83288 is a clinical development candidate for the treatment of severe malaria
Source: Nat Commun. 2017 Jan 31;8:14193. doi: 10.1038/ncomms14193 (PMC5290327; doi:10.1038/ncomms14193)
Supplement: Supplementary Information — Supplementary Figures, Supplementary Tables, Supplementary Notes and Supplementary References [file ncomms14193-s1.pdf]

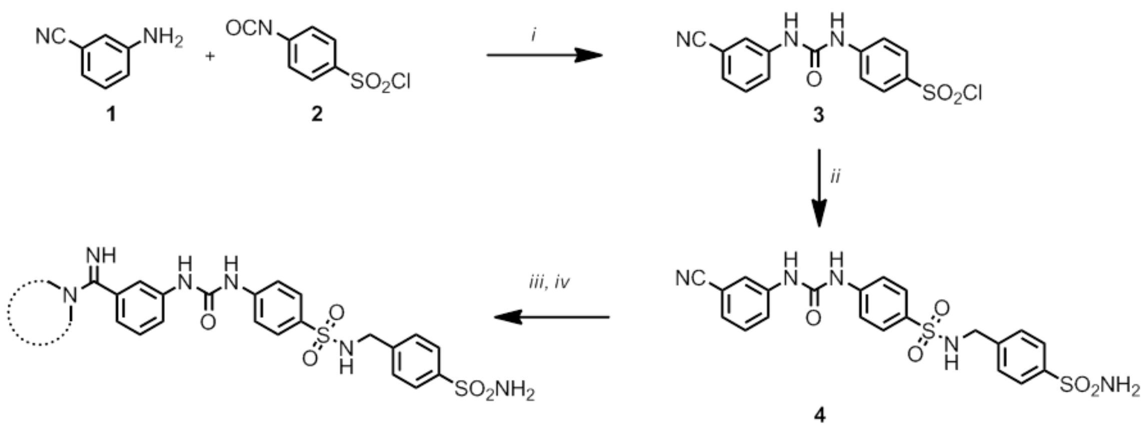

Supplementary Figure 1: Synthesis of SC81458 and SC83288. Scheme of the 4 step synthesis. Reagents and conditions: (i) acetonitrile, RT; (ii) 4-(aminomethyl)benzenesulfonamide, diisopropyletylamine, dimethylformamide; (iii) 4M HCl in dioxane, methanol; (iv) secondary amine, diisopropyletylamine, dimethylformamide, 70°C. Further detail is provided in the Materials and Methods section.

a

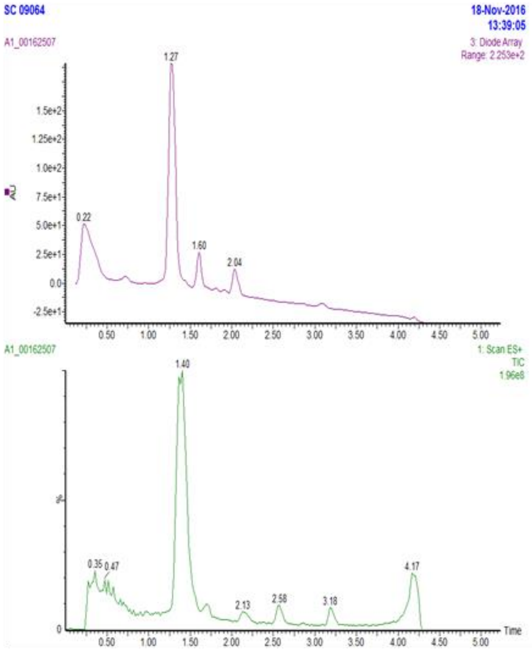
SC 09064
18-Nov-2016
13:39:05
A1_00162507
3: Diode Array
Range: 2.253e+2
1.27
0.22
1.60
2.04
AU
A1_00162507
1: Scan ES+
TIC
1.96e8
1.40
0.35
0.47
2.13
2.58
3.18
4.17
%
Time

b

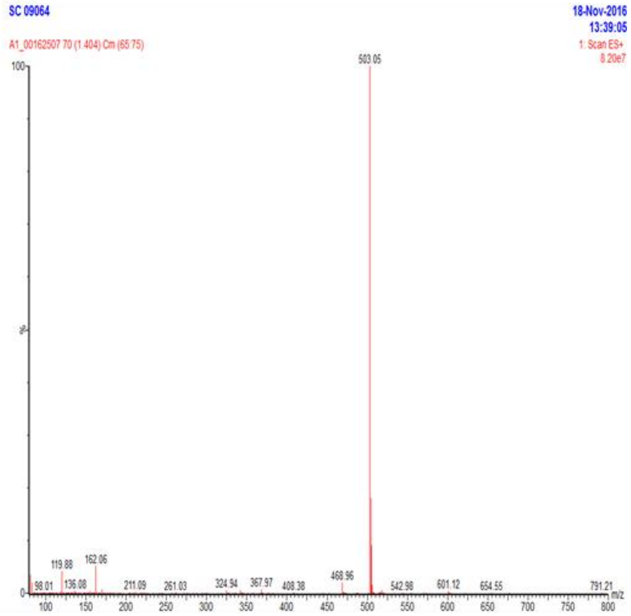
SC 09064
18-Nov-2016
13:39:05
A1_00162507 70 (1.404) Cm (65:75)
1: Scan ES+
8.20e7
503.05
119.88
162.06
98.01
136.08
211.09
261.03
324.94
367.97
408.38
468.96
542.98
601.12
654.55
791.21
%
m/z

c

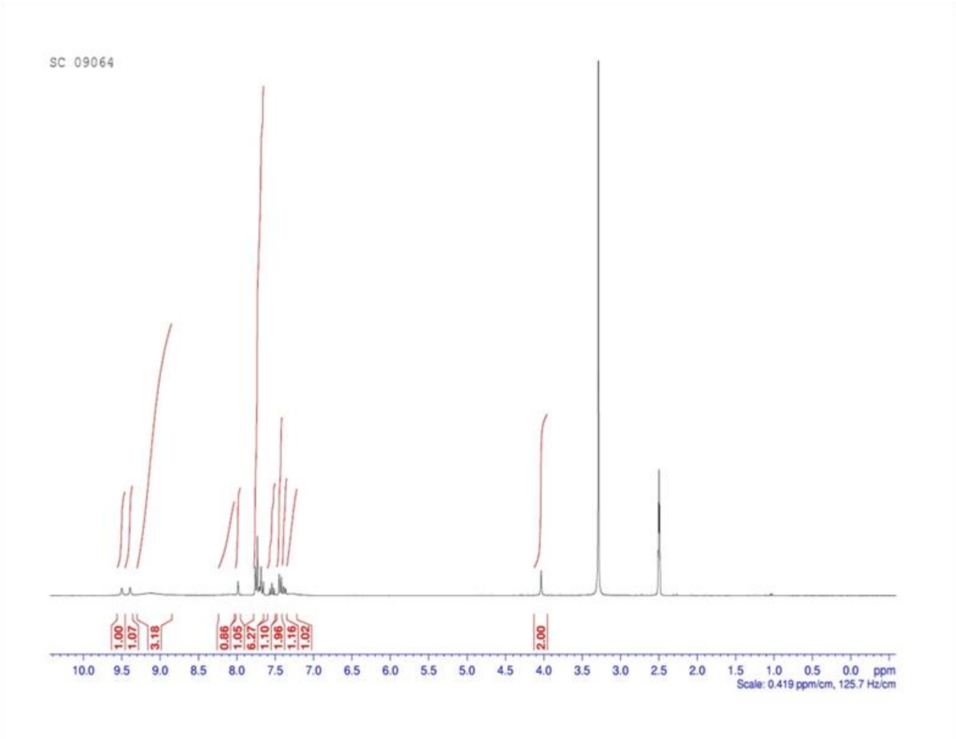
SC 09064
1.00
1.07
3.18
0.86
1.05
6.27
1.10
1.96
1.16
1.02
2.00
ppm
Scale: 0.419 ppm/cm, 125.7 Hz/cm

d

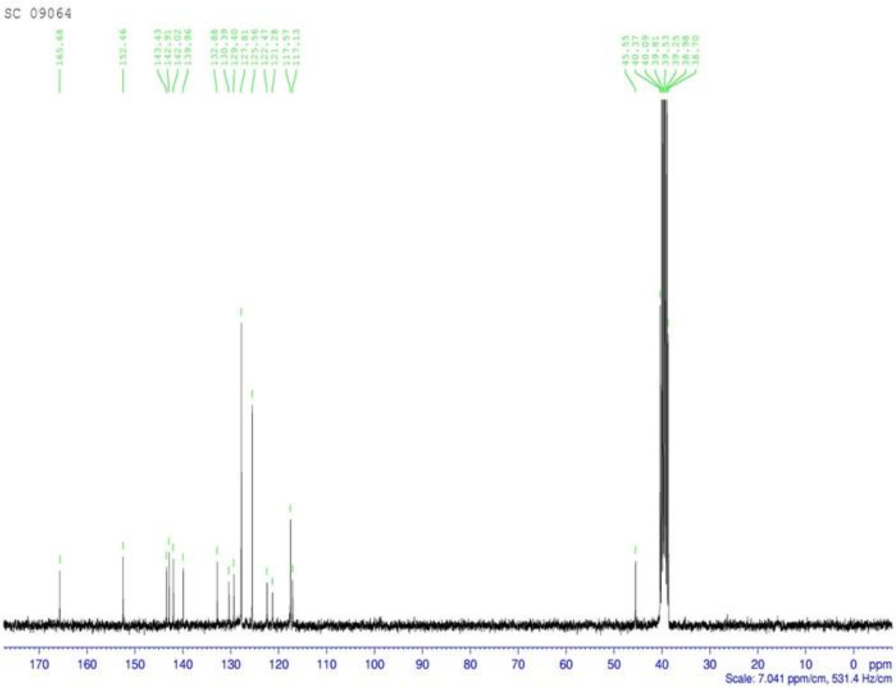
SC 09064
165.68
152.46
143.43
142.91
142.02
139.96
132.88
130.39
129.40
127.81
125.56
122.47
121.28
117.57
117.13
45.55
40.37
40.09
39.81
39.53
39.25
38.98
38.70
170 160 150 140 130 120 110 100 90 80 70 60 50 40 30 20 10 0 ppm
Scale: 7.041 ppm/cm, 531.4 Hz/cm

e

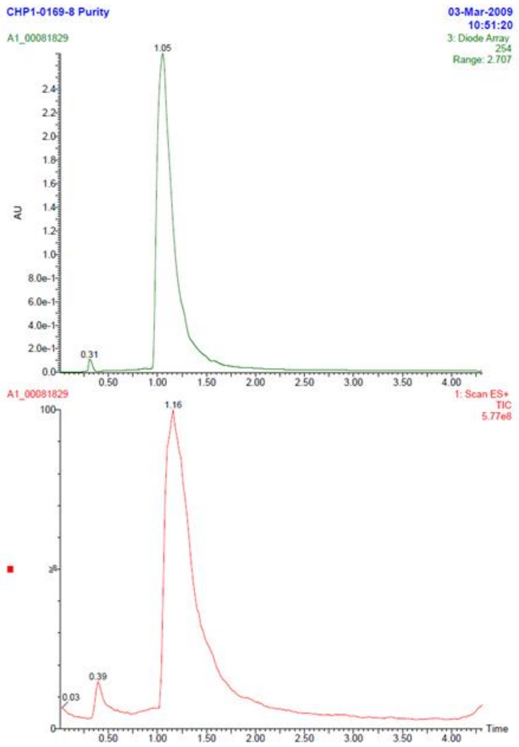
CHP1-0169-8 Purity
03-Mar-2009
10:51:20
A1_00081829
3: Diode Array
254
Range: 2.707
AU
1.05
0.31
A1_00081829
1: Scan ES+
TIC
5.77e8
1.16
0.39
0.03
%
Time

f

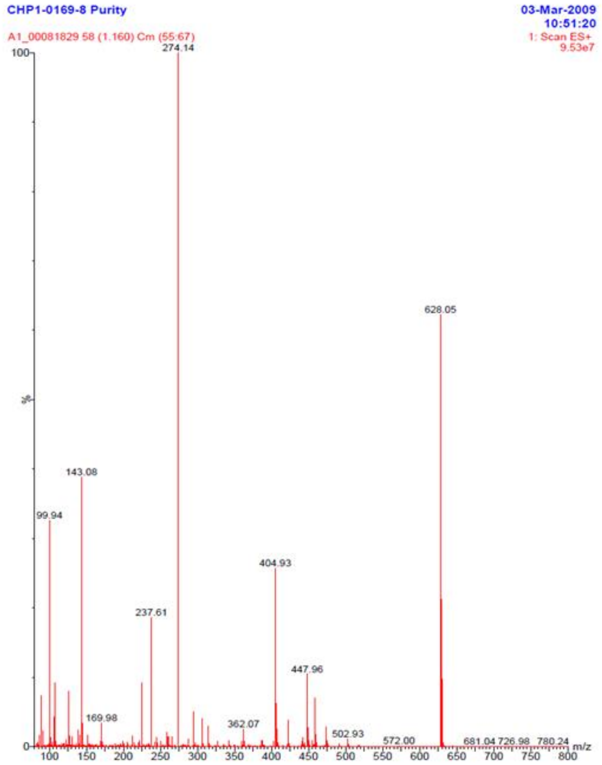
CHP1-0169-8 Purity
03-Mar-2009
10:51:20
A1_00081829 58 (1.160) Cm (55:67)
1: Scan ES+
9.53e7
274.14
628.05
143.08
99.94
404.93
237.61
447.96
169.98
362.07
502.93
572.00
681.04 726.98 780.24
%
m/z

g

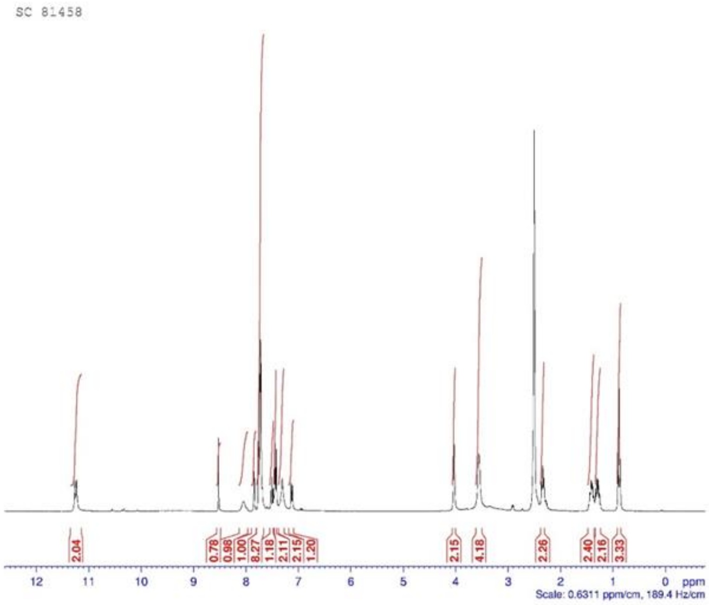
SC 81458
2.04
0.78
0.98
1.00
8.27
1.18
2.11
2.15
1.20
2.15
4.18
2.26
2.40
2.16
3.33
ppm
Scale: 0.6311 ppm/cm, 189.4 Hz/cm

h

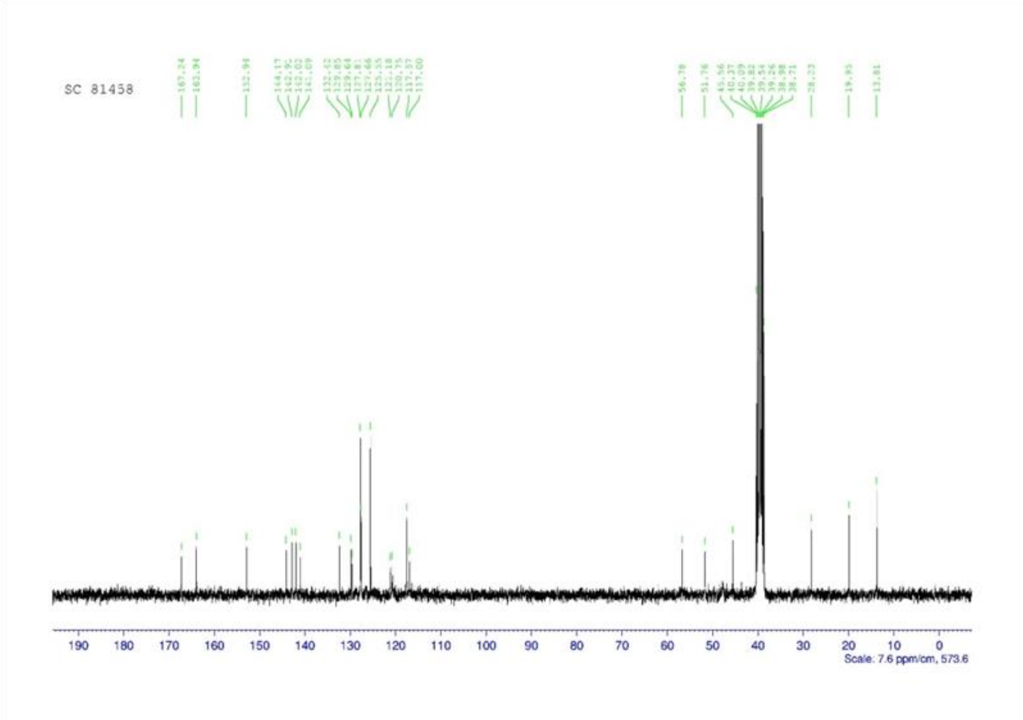
SC 81458
167.24
163.94
152.94
144.17
141.09
132.42
129.85
129.64
127.66
125.55
120.75
117.57
117.00
56.78
51.76
45.56
40.37
40.09
39.82
39.54
39.26
38.98
38.71
28.23
19.95
13.81
190 180 170 160 150 140 130 120 110 100 90 80 70 60 50 40 30 20 10 0
Scale: 7.6 ppm/cm, 573.6

i

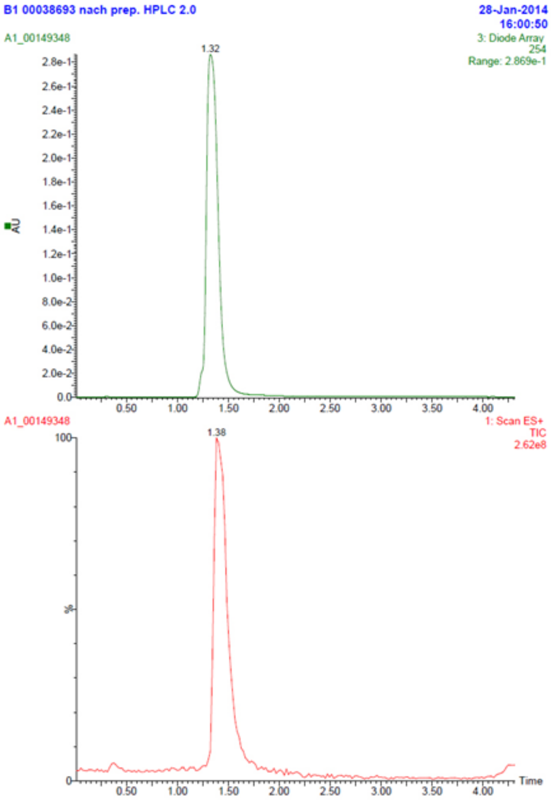
B1 00038693 nach prep. HPLC 2.0
28-Jan-2014
16:00:50
A1_00149348
3: Diode Array
254
Range: 2.869e-1
1.32
AU
Time
A1_00149348
1: Scan ES+
TIC
2.62e8
1.38
%

j

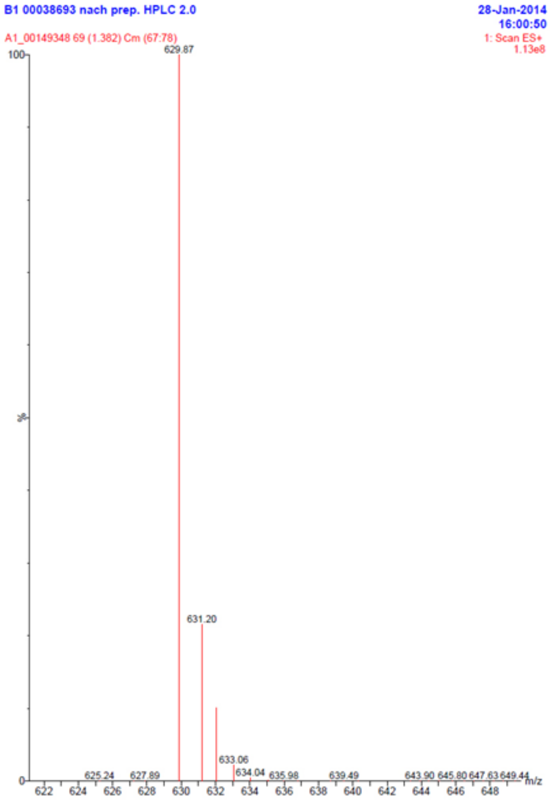
B1 00038693 nach prep. HPLC 2.0
28-Jan-2014
16:00:50
A1_00149348 69 (1.382) Cm (67:78)
1: Scan ES+
1.13e8
629.87
631.20
633.06
634.04
625.24
627.89
635.98
639.49
643.90
645.80
647.63
649.44
m/z
%

k

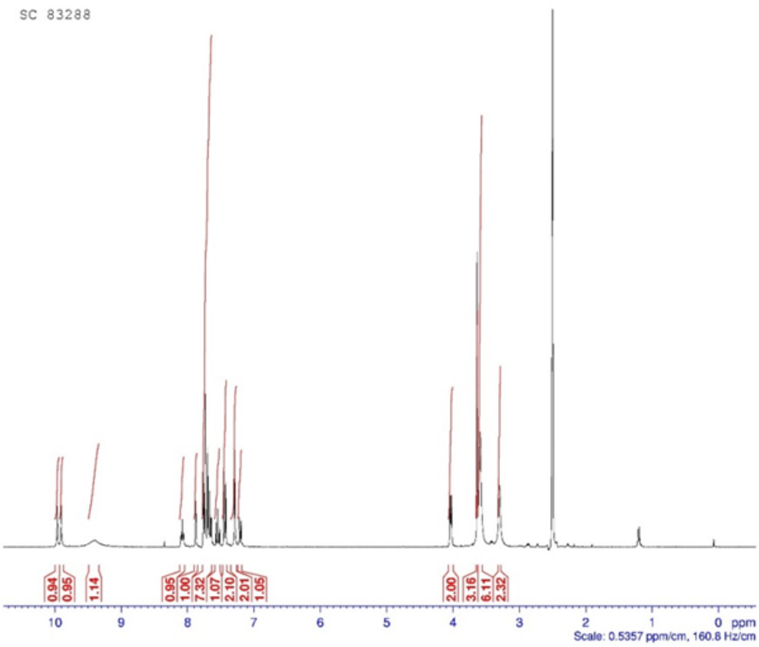
SC 83288
0.94
0.95
1.14
0.95
1.00
7.32
1.07
2.10
2.01
1.05
2.00
3.16
6.11
2.32
ppm
Scale: 0.5357 ppm/cm, 160.8 Hz/cm

l

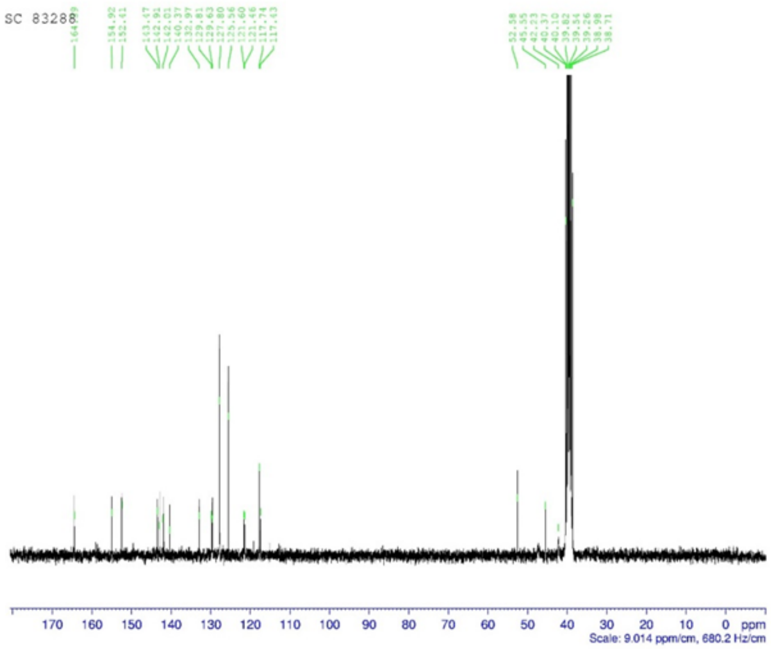

Supplementary Figure 2: Characterization of SC09064, SC81458 and SC83288. MS chromatogram (**a**), MS spectrum (**b**), ^{1}H NMR spectrum (**c**) and ^{13}C NMR spectrum (**d**) of the hit compound SC09064. MS chromatogram (**e**), MS spectrum (**f**), ^{1}H NMR spectrum (**g**) and ^{13}C NMR spectrum (**h**) of the lead compound SC81458. MS chromatogram (**i**), MS spectrum (**j**), ^{1}H NMR spectrum (**k**) and ^{13}C NMR spectrum (**l**) of the lead compound SC83288.

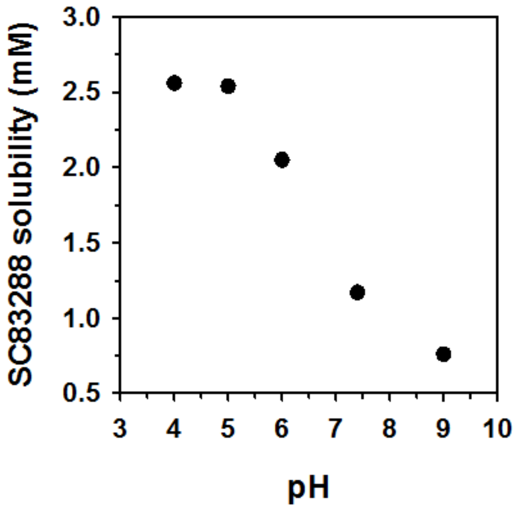

Supplementary Figure 3: Solubility profile of SC83288 in phosphate buffered saline at different pH.

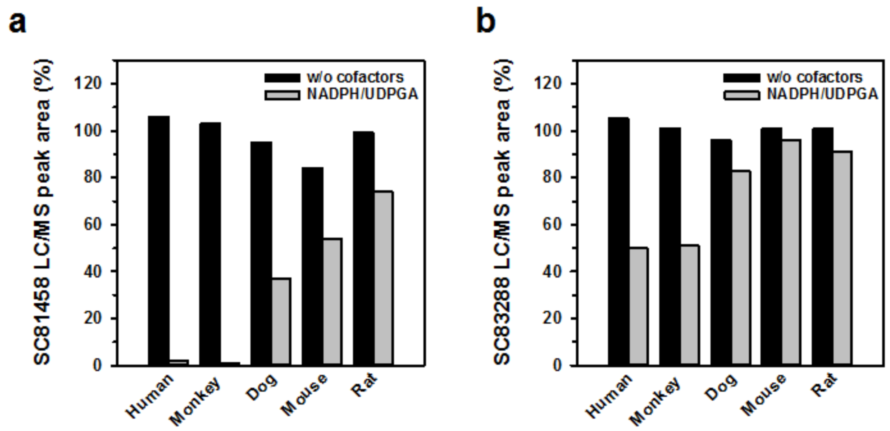

Supplementary Figure 4: Stability of SC81458 and SC83288 in microsomal extracts from different species. Relative LC/MS peak areas of SC81458 (**a**) or SC83288 (**b**) after 60 min of incubation at 37°C with (gray) and without (black) cofactors in microsomal extracts from human, monkey, dog, mouse, and rat.

a

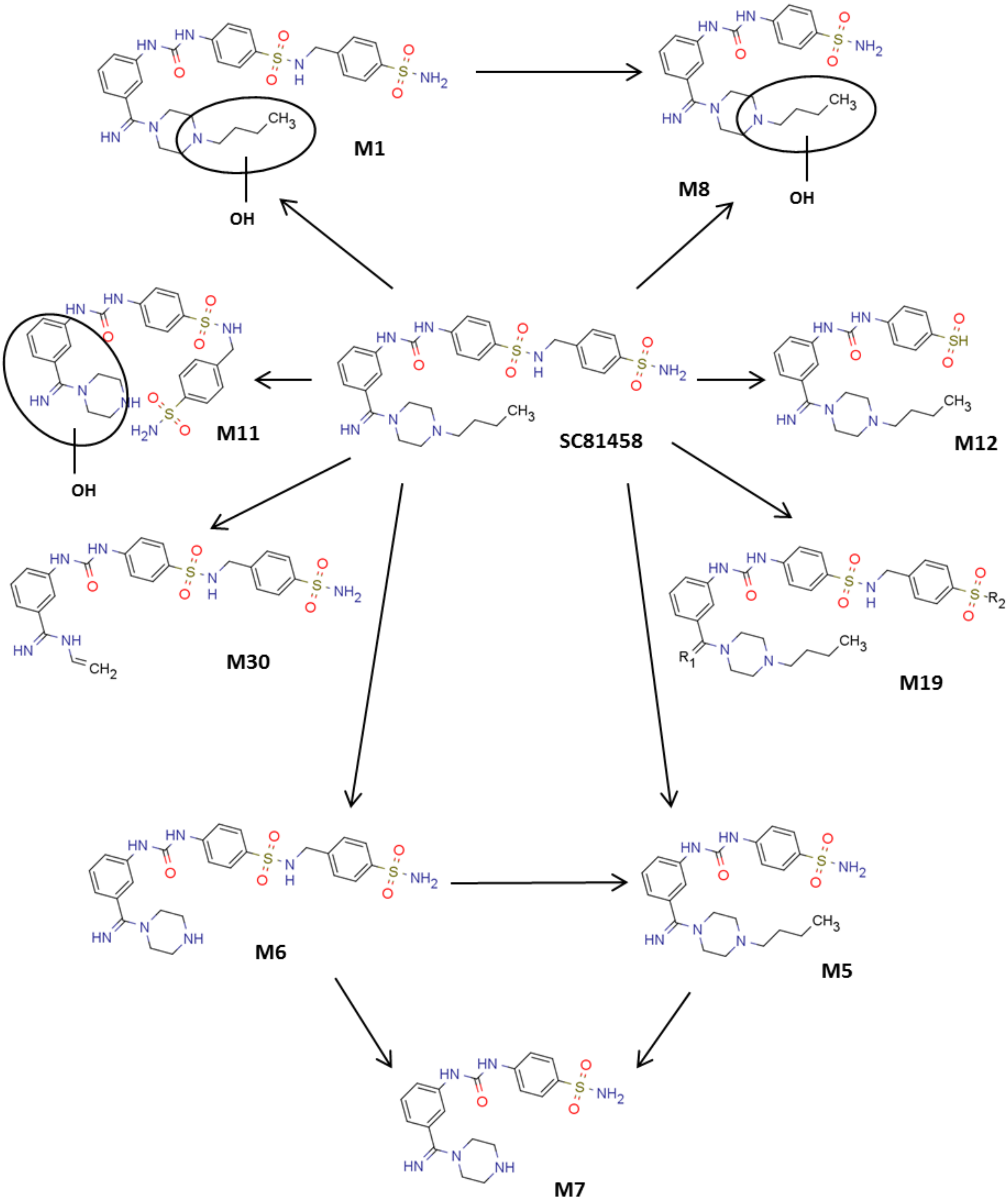

b

	Metabolite identification	m/z found	m/z calc.	Time (min)
SC81458	Parent	628.2374	628.2376	2.77
M1	Hydroxylation in the butyl side chain or in piperazine ring	644.2320<br>529.1321<br>303.1822	644.2325<br>529.1325<br>303.1821	2.58
M2	Hydroxylation (or N-oxidation)	644.2316	644.2325	2.49
M3	Dehydrogenation	626.2216	626.2219	2.72
M4	Dehydrogenation + hydroxylation (or N-oxidation)	642.2173	642.2169	2.6
M5	N-dealkylation (loss of C7H7NSO2)	459.2179<br>3601132	459.2178<br>3601130	2.65
M6	N-dealkylation (loss of C4H8)	572.1779	572.1750	2.38
M7	N-dealkylation (loss of C4H8) + N-dealkylation (loss of C7H7NSO2)	403.1541	403.1552	1.35
M8	Hydroxylation in the butyl side chain or in piperazine ring + N-dealkylation (loss of C7H7NSO2)	475.2127<br>360.1097	475.2128<br>360.1130	2
M9	Dihydroxylation (/N-oxidation)	660.2282	660.2274	2.44
M10	Dihydroxylation (/N-oxidation)	660.2292	660.2274	2.62
M11	Hydroxylation/N-oxidation in area from piperazine ring to carbamide bond + N-dealkylation (loss of C4H8)	588.1708<br>247.1183	588.1699<br>247.1195	2.47
M12	S-N bond dissociation (loss of C7H8N2SO2)	444.2072<br>287.1884<br>143.1554	444.2069<br>2871872<br>143.1548	2.43
M13	Dehydrogenation + (N-dealkylation (loss of C7H7NSO2)	457.2025	457.2022	2.59
M14	Dehydrogenation + (N-dealkylation (loss of C7H7NSO2)	457.2033	457.2022	2.79
M15	Hydroxylation (or N-oxidation)+ dehydrogenation + N-dealkylation (loss of C7H7NSO2)	473.1971	473.1971	2.03
M16	Hydroxylation (or N-oxidation)+ dehydrogenation + N-dealkylation (loss of C7H7NSO2)	473.1977	473.1971	2.37
M17	Hydroxylation (or N-oxidation)+ dehydrogenation + N-dealkylation (loss of C7H7NSO2)	473.1974	473.1971	2.47
M18	Hydroxylation (or N-oxidation)+ dehydrogenation + N-dealkylation (loss of C7H7NSO2)	473.1982	473.1971	2.79
M19	oxidative deamination	629.222	629.2216	2.69
M20	Dihydroxylation (or N-oxidation)+dehydrogenation	658.2108	658.2118	2.32
M21	Dihydroxylation (or N-oxidation)+dehydrogenation	658.2114	658.2118	2.5
M22	Dihydroxylation (or N-oxidation)+ dehydrogenation + N-dealkylation (loss of C7H7NSO2)	489.1924	489.1920	1.39
M23	Dihydroxylation (or N-oxidation)+ dehydrogenation + N-dealkylation (loss of C7H7NSO2)	489.1921	489.1920	1.65
M24	Dihydroxylation (or N-oxidation)+ dehydrogenation + N-dealkylation (loss of C7H7NSO2)	489.1921	489.1920	1.74
M25	Dihydroxylation (or N-oxidation)+ dehydrogenation + N-dealkylation (loss of C7H7NSO2)	489.1925	489.1920	1.86
M26	N-dealkylation (loss of C4H8)+ N-dealkylation (loss of C7H7NSO2)+acetylation	447.1826	4471815	1.6
M27	Dehydrogenation + hydroxylation (or N-oxidation)	642.2162	642.2169	2.65
M28	Dehydrogenation	626.2193	626.2219	2.87
M29	dehydrogenation + hydroxylation (or N-oxidation)	642.2173	642.2169	2.87
M30	N-dealkylation (loss of C4H8NC2H5)	529.1328	529.1328	2.57

c

	Human (%)	Monkey (%)	Dog (%)	Mouse (%)	Rat (%)
SC81458					
M1	**15**	**29.2**	**24.7**	**18.8**	**41.9**
M2	0.1	0.2	0.2	1	0.4
M3	0.1		1.3	0.5	1.3
M4	2.4	**8.7**	1.3	0.7	1.1
M5	**51.4**	0.1	3.1	**49**	**23.3**
M6	**7**	**10.5**	**63.6**	**22.2**	**27.8**
M7	4.7	**7.6**	1.6	-	0.3
M8	**16.3**	**25.9**	0.5	0.2	0.5
M9	0.1	0.3	0.1	-	-
M10	0.1		0.1	-	0.4
M11	0.1	0.3	3.1	3.8	1.8
M12	0.2	0.1	0.1	0.5	0.3
M13	1.3		0.1	-	0.1
M14	0.2		-	-	-
M15	0.6	**10.5**	-	-	-
M16	0.1		-	-	-
M17	0.1		-	-	-
M18	0.1		-	-	-
M19	0.2	0.1	0.3	0.6	0.4
M20	-	.1	0.1	-	-
M21	-	1.7	-	-	-
M22	-	0.2	-	-	-
M23	-	0.2	-	-	-
M24	-	0.2	-	-	-
M25	-	0.8	-	-	-
M26	-	2.5	-	-	-
M27	-	-	-	-	0.3
M28	-	-	-	0.5	-
M29	-	-	-	0.4	-
M30	-	-	-	1.8	-

Supplementary Figure 5: Metabolites and biotransformation of SC81458 in microsomal extracts. (**a**) Suggested metabolic reactions and biotransformation sites of SC81458. (**b**) Description of the biotransformations leading to detected fragments by LC/ESI/TOF-MS analysis. (**c**) Shares of each SC81458 metabolite from the total combined metabolite peak area in each studied species. Main metabolites having share of 5% or more of the total combined peak area of all metabolites are bolded.

A

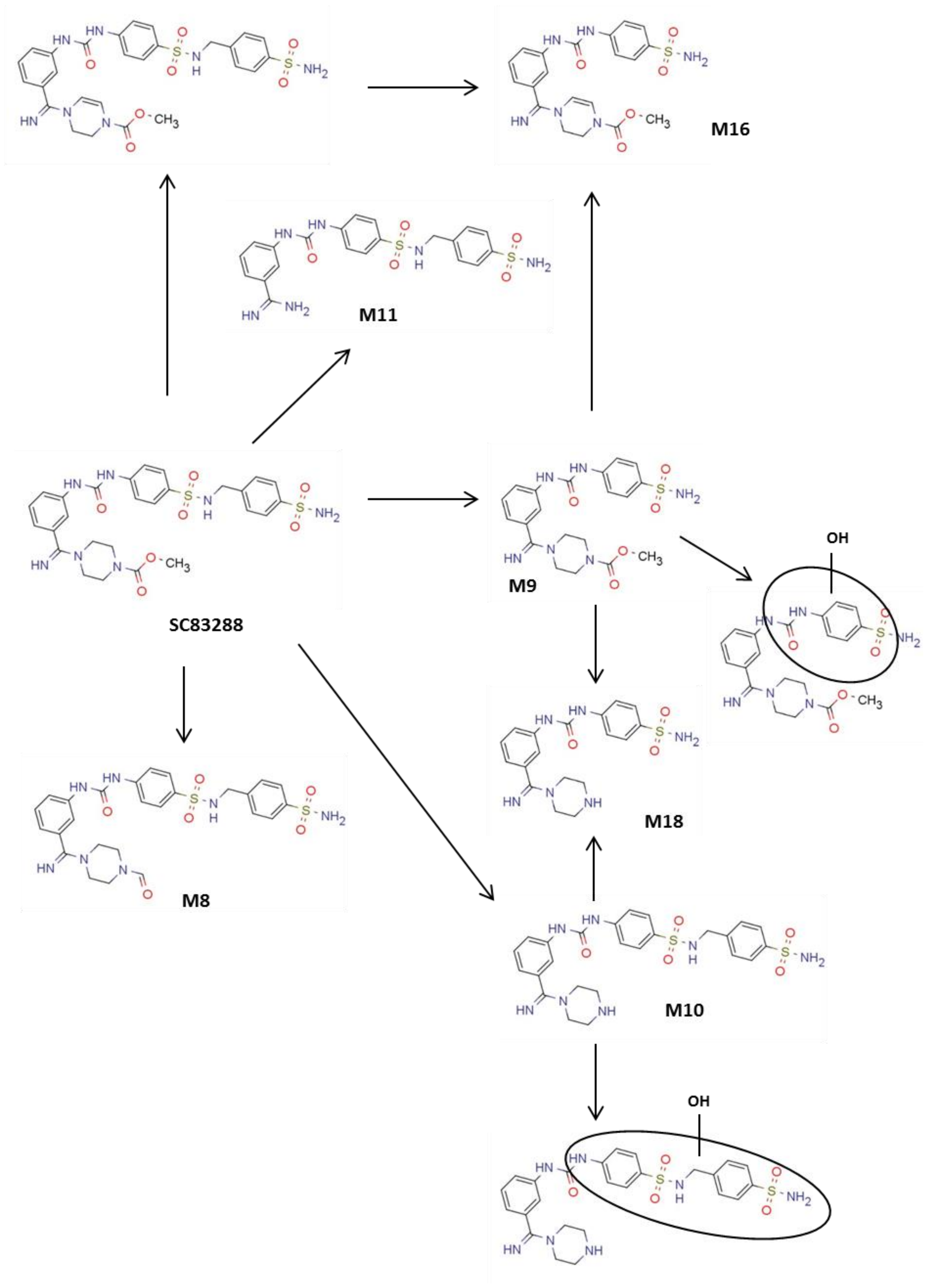

b

	Metabolite identification	m/z found	m/z calc.	Time (min)
SC83288	Parent	630.1797	630.1805	2.80
M1	Hydroxylation/N-oxidation	646.1724	646.1754	3.16
M2	Hydroxylation/N-oxidation	646.1727	646.1754	2.83
M3	Hydroxylation/N-oxidation	646.1755	646.1754	2.79
M4	Hydroxylation/N-oxidation	646.1756	646.1754	2.72
M5	Dehydrogenation	628.1612	628.1648	2.86
M6	2 × Hydroxylation/N-oxidation	662.1668	662.1703	2.72
M7	2 × Hydroxylation/N-oxidation + hydrogenation	664.1833	664.1859	2.68
M8	Cleavage of CH2O	600.1713	600.1699	2.62
M9	N-dealkylation (loss of C7H7NO2S)	461.1611	461.1607	2.57
	Fragment (loss of C6H8N2O2S)	289.1302	289.1301	
M10	N-dealkylation (loss of C2H2O2)	572.1750	572.1750	2.30
	Fragment (loss of C7H7NO2S)	403.1552	403.1552	
	Fragment (loss of C13H15N3O4S2)	231.1251	231.1246	
M11	Piperazine ring opening followed by N-dealkylation (loss of C6H9NO2)	503.1166	503.1171	2.48
M12	Hydroxylation/N-oxidation (to C6H7N2O2S -area) + N-dealkylation (loss of C7H7NO2S)	477.1537	477.1556	2.92
	Fragment (loss of C6H8N2O3S)	289.1313	289.1301	
M13	Hydroxylation/N-oxidation + N-dealkylation (loss of C7H7NO2S)	477.1527	477.1556	2.47
M14	Hydroxylation/N-oxidation + N-dealkylation (loss of C7H7NO2S)	477.1547	477.1556	2.41
M15	Hydroxylation/N-oxidation (to C13H14N3O4S2 -area) + N-dealkylation (loss of C2H2O2)	588.1703	588.1699	2.63
	Fragment (loss of C11H13N3O3S2)	289.1305	289.1301	
M16	Dehydrogenation + N-dealkylation (loss of C7H7NO2S)	459.1466	459.1451	2.63
M17	2 × Hydroxylation/N-oxidation + hydrogenation + N-dealkylation (loss of C7H7NO2S)	495.1657	495.1662	2.42
M18	N-dealkylation (loss of C7H7NO2S) + N-dealkylation (loss of C2H2O2)	4031.548	403.1552	0.57
	Fragment (loss of C6H8N2O2S)	231.1251	231.1246	
M19	Hydroxylation/N-oxidation + N-dealkylation (loss of C7H7NO2S) + N-dealkylation (loss of C2H2O2)	419.1515	419.1502	2.32
M20	2 × Hydroxylation/N-oxidation + N-dealkylation (loss of C7H7NO2S) + N-dealkylation (loss of C2H2O2)	435.1467	435.1451	2.44

c

	Human (%)	Monkey (%)	Dog (%)	Mouse (%)	Rat (%)
SC83288					
M1	-	-	-	-	0.7
M2	-	0.2	-	-	0.7
M3	-	-	-	-	1.1
M4	1.2	1.4	1.1	**14.3**	3.5
M5	0.2	0.4	-	2.2	0.3
M6	0.1	0.2	-	2.6	0.6
M7	0.4	0.6	-	3.4	0.9
M8	0.1	0.2	0.5	0.9	0.6
M9	**82**	**72.3**	4.8	**69.9**	**69**
M10	**13.3**	**16.2**	**87.3**	**5**	**19.7**
M11	0.9	1.1	-	-	-
M12	0.2	0.1	-	0.5	0.6
M13	0.3	0.3	-	-	-
M14	0.2	0.3	-	-	-
M15	0.2	0.6	**6.3**	1.5	2
M16	0.2	0.2	-	-	-
M17	0.1	0.2	-	-	-
M18	0.3	**5.4**	-	-	0.3
M19	-	0.2	-	-	-
M20	0.2	0.2	-	-	-

Supplementary Figure 6: Metabolites and biotransformation of SC83288 in microsomal extracts. (**a**) Suggested metabolic reactions and biotransformation sites of SC83288. (**b**) Description of the biotransformations leading to the detected fragments by LC/ESI/TOF-MS analysis. (**c**) Shares of each SC83288 metabolite from the total combined metabolite peak area in each studied species. Main metabolites having share of 5% or more of the total combined peak area of all metabolites are bolded.

a

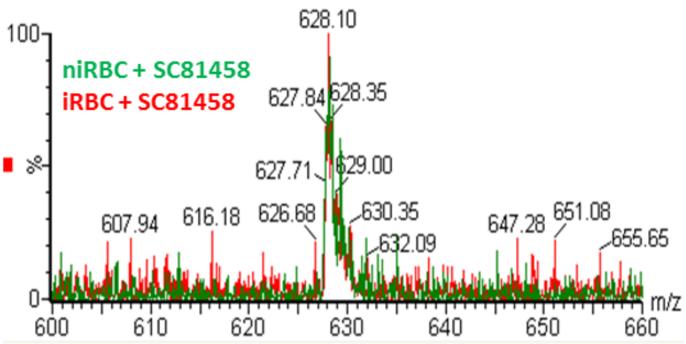
niRBC + SC81458
iRBC + SC81458
628.10
627.84
628.35
627.71
629.00
607.94
616.18
626.68
630.35
632.09
647.28
651.08
655.65
100
%
0
m/z
600
610
620
630
640
650
660

b

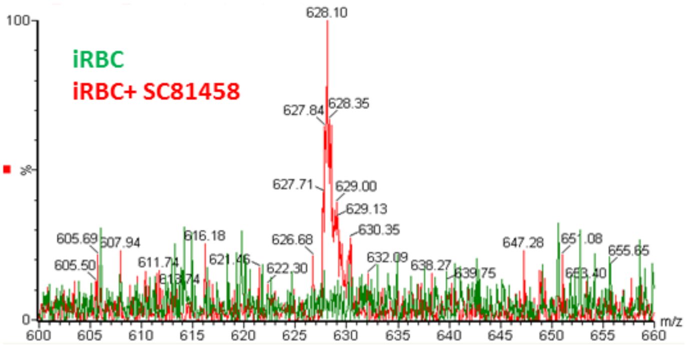
iRBC
iRBC+ SC81458
628.10
627.84
628.35
627.71
629.00
629.13
630.35
605.69
607.94
616.18
626.68
647.28
655.65
605.50
611.74
621.46
622.30
632.09
638.27
639.75
100
%
0
m/z
600
605
610
615
620
625
630
635
640
645
650
655
660

c

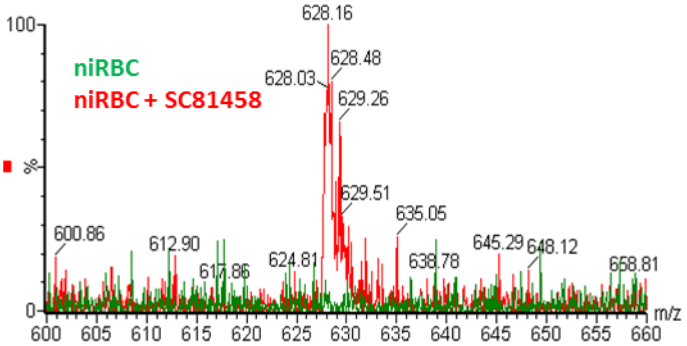
niRBC
niRBC + SC81458
628.16
628.48
628.03
629.26
629.51
635.05
600.86
612.90
624.81
645.29
648.12
638.78
658.81
100
%
0
m/z
600
605
610
615
620
625
630
635
640
645
650
655
660

d

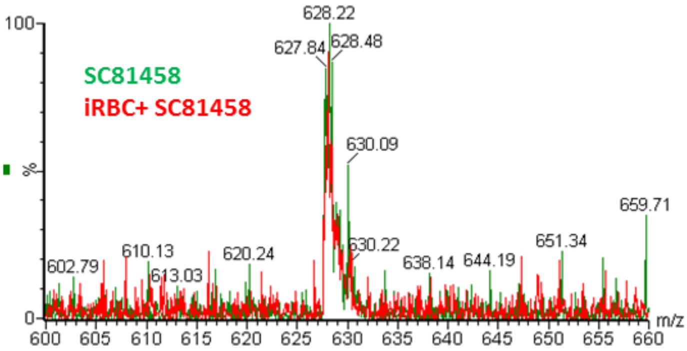
SC81458
iRBC+ SC81458
628.22
627.84
628.48
630.09
659.71
610.13
620.24
651.34
602.79
613.03
630.22
638.14
644.19
100
%
0
m/z
600
605
610
615
620
625
630
635
640
645
650
655
660

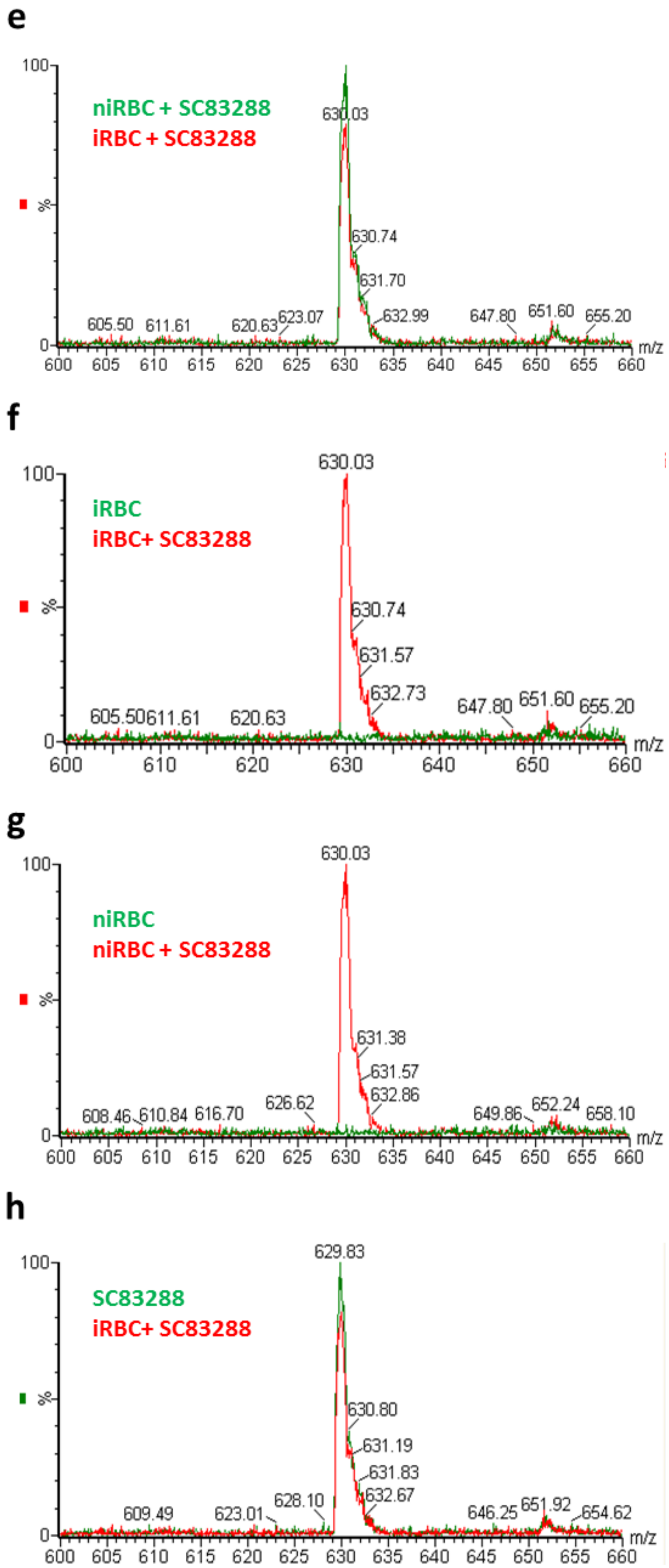

Supplementary Figure 7: Stability of SC81458 and SC83288 in blood cell culture. SC81458 and SC83288 (2 µM) were incubated with *P. falciparum*-infected erythrocytes, uninfected erythrocytes, or without any cells for 6 h at 37°C before the integrity of the

compound was investigated using MS chromatography following acetonitrile precipitation. (**a, e**) SC81458 (**a**) and SC83288 (**e**) with infected erythrocytes (iRBC, red) or uninfected erythrocytes (niRBC, green). (**b, f**) Infected erythrocytes incubated with 0.04 % DMSO/PBS (iRBC, green) or infected erythrocytes incubated with SC81458 (**b**; iRBC+SC81458, red) or SC83288 (**f**; iRBC+SC83288, red). (**c, g**) Uninfected erythrocytes incubated with 0.04 % DMSO/PBS (niRBC, green) or uninfected erythrocytes incubated with SC81458 (**c**; niRBC+SC81458, red) or SC83288 (**g**; niRBC+SC83288, red). (**d, h**) Infected erythrocytes incubated with SC81458 (**d**; iRBC+SC81458, red) or SC83288 (**h**; iRBC+SC83288, red), and SC81458 (**d**; SC81458, green) or SC83288 (**h**; SC83288, green) incubated in culture medium.

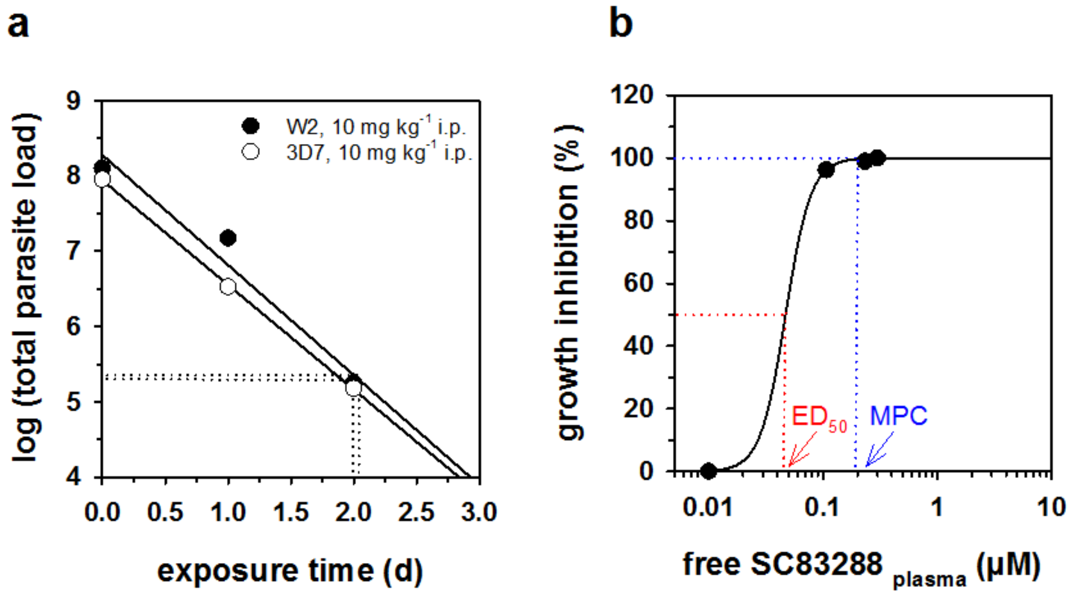

Supplementary Figure 8: Cure rate of SC83288. (**a**) Reduction of the parasite load in NSG mice engrafted with human erythrocytes with time following treatment with 10 mg kg^{-1} i.p. SC83288 once a day for four days. The data were taken from Figures 6C and D. The percent parasitemia was converted to total parasite load by assuming a total red blood cell count per mouse of ~1.5 x 10^{10} red blood cells[1]. The dotted lines show the parasite reduction ratio (PRR), i.e. the reduction of the parasite load over one asexual replication cycle of the parasite, here 48 h. (**b**) Inhibition of *P. falciparum* growth in NSG mice engrafted with human erythrocytes after 2 days treatment with 10 mg kg^{-1} i.p. SC83288. Growth inhibition is analyzed as a function of the free SC83288 plasma concentration. The minimum parasiticidal concentration (MPC) and the ED_{50} are depicted as blue and red dotted lines, respectively.

a

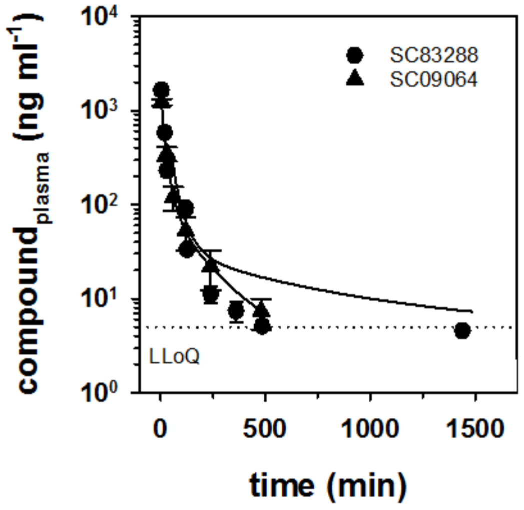

b

Compound	SC83288	SC09064
Application	i.v.	
Species	rat	
Dose (mg kg^{-1})	1	1
$AUC_{0\text{-}inf}$ (ng h ml^{-1})	709	732
C_0 (ng ml^{-1})	2188	1409
C_{max} (ng ml^{-1})	1641	1225
t_{max} (min)	5	5
$t_{1/2\ initial}$ (min)	9	16
$t_{1/2\ terminal}$ (min)	625	131
$t_{1/2\ effective}$ (min)	52	44
$MRT_{0\text{-}t}$ (h)	1.25	1.05
CL (ml h^{-1} kg^{-1})	1410	1366
V_C (ml kg^{-1})	457	710

c

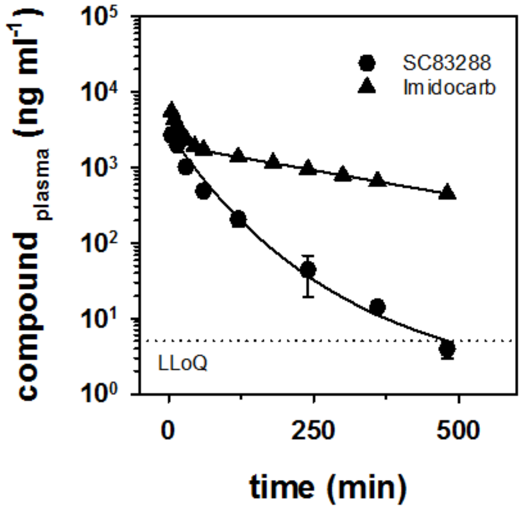

d

Compound	SC83288	Imidocarb
Application	i.v.	
Species	dog	
Dose (mg kg^{-1})	1	1
$AUC_{0\text{-}inf}$ (ng h ml^{-1})	1331	11922
C_0 (ng ml^{-1})	3282	6801
C_{max} (ng ml^{-1})	2685	5518
t_{max} (min)	5	5
$t_{1/2\ initial}$ (min)	12	21
$t_{1/2\ terminal}$ (min)	90	224
$t_{1/2\ effective}$ (min)	35	112
$MRT_{0\text{-}t}$ (h)	0.84	2.70
CL (ml h^{-1} kg^{-1})	751	84
V_C (ml kg^{-1})	305	147

Supplementary Figure 9: Comparison of the pharmacokinetic profiles of SC09064, SC83288, and imidocarb. (**a**) Mean plasma concentration (normalized to a dose of 1 mg kg^{1}) of SC09064 (closed triangles) and SC83288 (closed circles) over time following i.v. administration in rats of 0.25 mg kg^{-1} and 2 mg kg^{-1}, respectively. The mean ± SEM are shown for at least three animals. Data for SC09064 were examined using a non-compartment analysis and relevant PK parameters are summarized in (**b**). (**c**) Mean plasma concentration (normalized to a dose of 1 mg kg^{1}) of SC83288 (closed circles) and imidocarb (closed triangles) over time following i.v. administration in dogs of 2 mg kg^{-1} and 4 mg kg^{-1}. The mean ± SEM are shown for at least three animals treated with SC83288. Data for imidocarb were extracted from Abdullah and Baggot (1983)[2] and relevant PK parameters are listed in (**d**). The dotted lines show the lower limit of quantification (LLoQ).

a

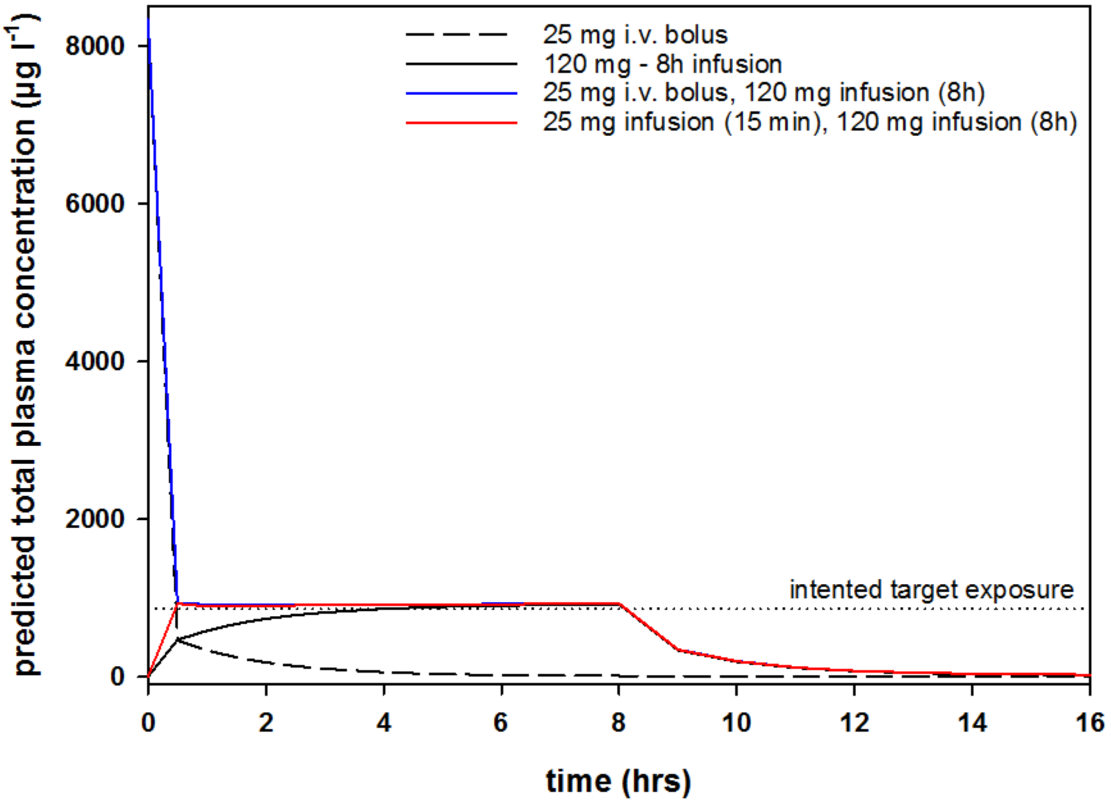

b

Parameter	Estimate	StdError	CV (%)
K_p	0.495	0.121	24.5
fd1	0.146	0.070	47.9
fd2	0.009	0.002	23.6
b	0.756	0.018	2.30
a	0.611	0.061	10.0
c	0.347	0.053	15.2
**Clearance (secondary parameter)**			
CL_{mouse}	0.042	0.005	12.0
CL_{monkey}	1.800	0.184	10.2
CL_{rat}	0.225	0.023	10.4
CL_{man}	15.189	1.860	12.2

Supplementary Figure 10: Simulations of humen PK profiles of SC83288 utilizing a minimal physiologically-based PK model. (**a**) Simulation of various dosing schemes. i.v. bolus of 25mg (dotted line); 8 hour infusion of 120 mg (black solid line); i.v. bolus of 25 mg as loading dose, followed by 8 hour infusion of 120 mg (blue solid line); and a combination of 15 min infusion of 25 mg followed by 8 hour infusion of 120 mg (red solid line). (**b**) Final parameter estimates and precisions of the fit of preclinical plasma PK data to mPBPK model.

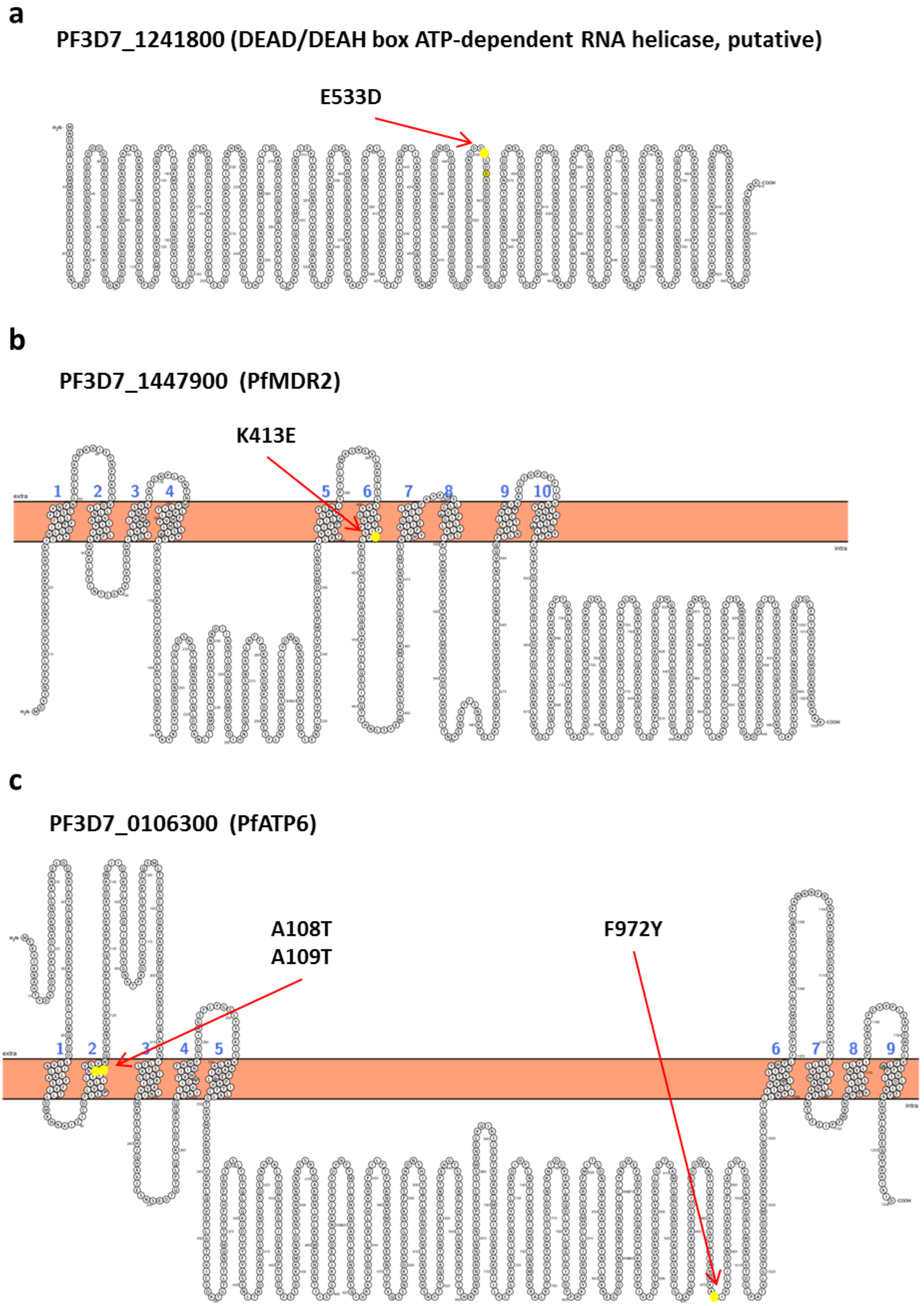
a
PF3D7_1241800 (DEAD/DEAH box ATP-dependent RNA helicase, putative)
E533D
b
PF3D7_1447900 (PfMDR2)
K413E
1
2
3
4
5
6
7
8
9
10
c
PF3D7_0106300 (PfATP6)
A108T
A109T
F972Y
1
2
3
4
5
6
7
8
9

d

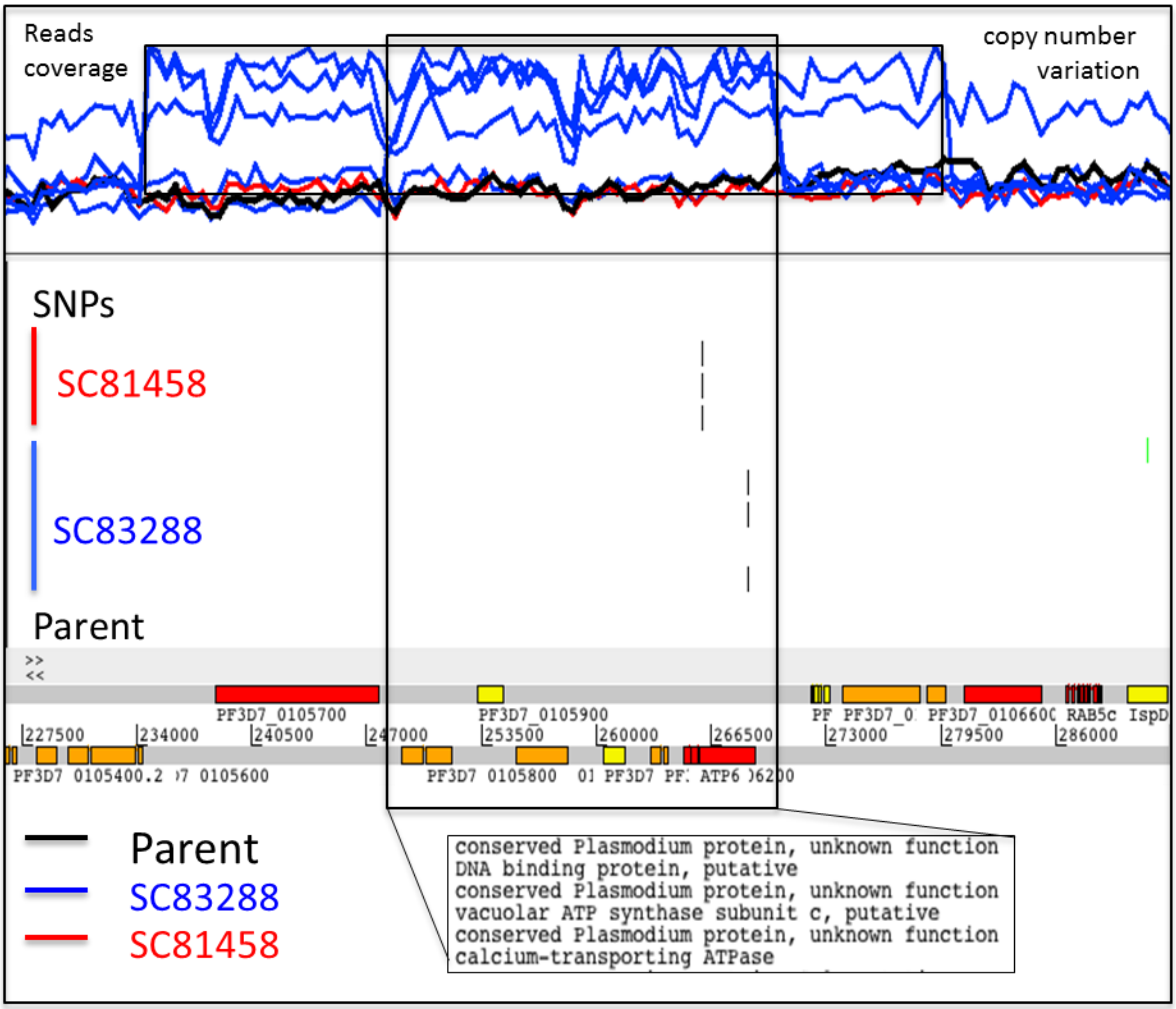

Supplementary Figure 11: Topological models and polymorphisms identified in gene products associated with SC81458 and SC83288 resistance. (**a**) The putative ATP-dependent RNA helicase DBP9, (**b**) PfMDR2; (**c**) PfATP6. The models were generated using the online tool Protter (version 1.0)[3]. (**d**) Snap shot showing amplification of the chromosome 1 domain containing PfATP6 in SC81458 and SC83288 selected clones.

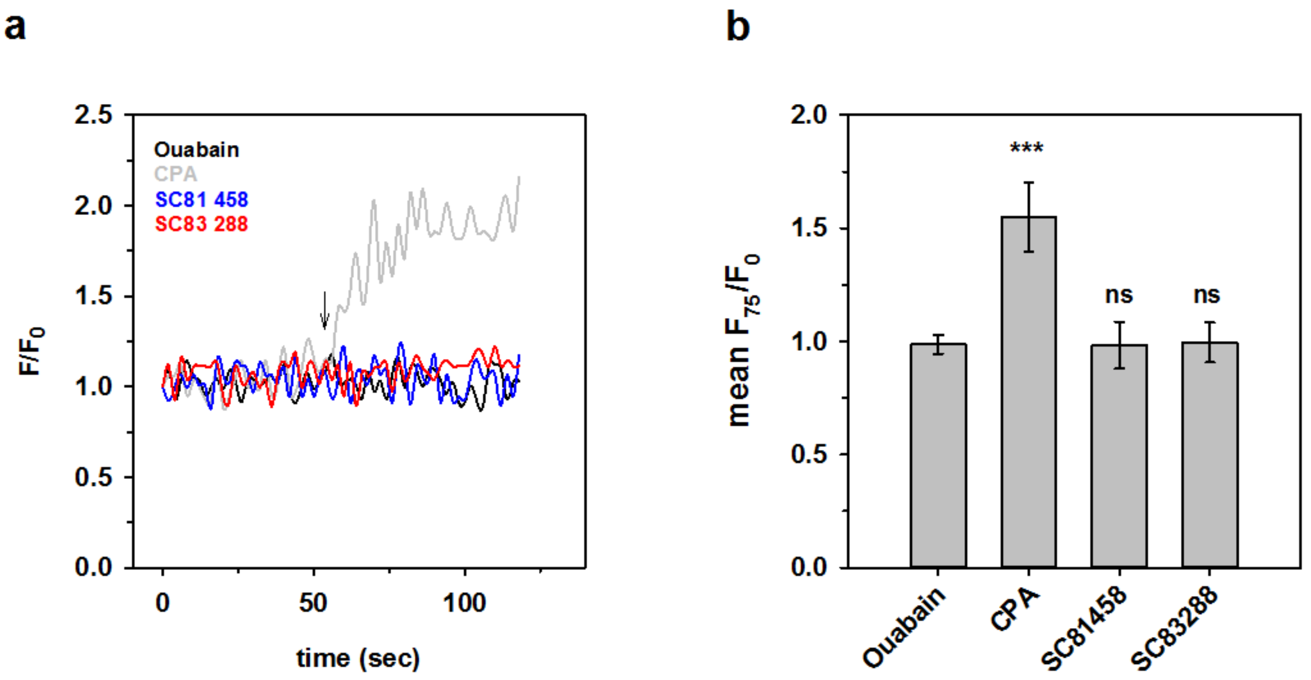

Supplementary Figure 12: SC81458 and SC83288 do not induced cytoplasmic Ca^{2+} responses in *P. falciparum*. *P. falciparum*-infected erythrocytes (trophozoites of the HB3 strain) were stained with the Ca^{2+} sensitive fluorochrome Fluo 4-AM (10 µM) and cytoplasmic Ca^{2+} transients were recorded in response to SC81458 (blue; 10 µM), SC83288 (red; 10 µM), ouabain (black; 10 µM), and CPA (gray; 10 µM), using a confocal live cell setup. (**a**) Representative Ca^{2+} traces in the parasite's cytoplasm. The mean fluorescence intensity (F) was normalized to the fluorescence intensity at time point zero (F_0). (**b**) The mean fluorescence intensity at time point 75 sec (F_{75}) was normalized to the fluorescence intensity at time point zero (F_0). The mean ± SEM are shown for at least five independent biological replicates with at least eight determinations in each replicate. Statistical significance was evaluated using the one way ANOVA Holm-Sidak test. ***, $p < 0.001$.

Supplementary Table 1: Compounds tested.

East-side optimization

East-Aryl-sulfone chain influence

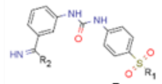

	R_1	R_2	IC_{50} [nM]
SC08165 *(423 Da)*	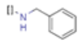	$-NH_2$	134
SC08985 *(422 Da)*	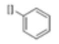	$-NH_2$	2990
SC09231 *(477 Da)*	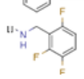	$-NH_2$	13,2
SC43796 *(466 Da)*	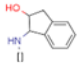	$-NH_2$	46,9
SC80166 *(576 Da)*	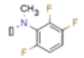	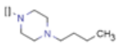	14,6
SC81475 *(598 Da)*	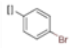	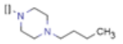	598
SC82341 *(603 Da)*	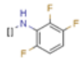	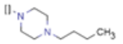	544
SC82343 *(549 Da)*	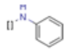	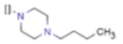	595
SC82746 *(529 Da)*	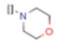	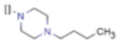	2580
SC82893 *(547 Da)*	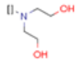	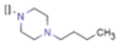	6250
SC82894 *(375 Da)*	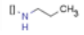	$-NH_2$	365
SC82895 *(501 Da)*	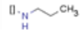	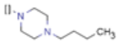	1760
SC85259 *(642 Da)*	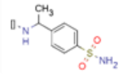	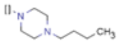	224
SC85343 *(565 Da)*	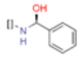	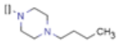	1550
SC86048 *(640 Da)*	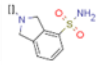	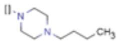	73,4
SC86610 *(663 Da)*	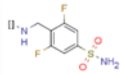	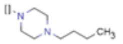	894
SC86714 *(645 Da)*	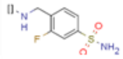	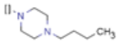	873
SC87211 *(634 Da)*	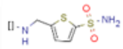	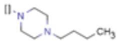	42,1

East-Aryl side-chain influence

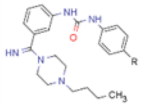

	R	IC_{50} [nM]
SC86354 *(617 Da)*	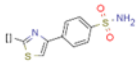	1710
SC86943 *(515 Da)*	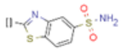	739

West-side optimization

Piperazine chain influence

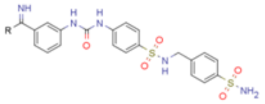

Piperazine chain

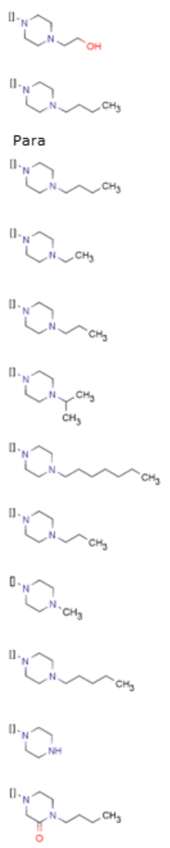

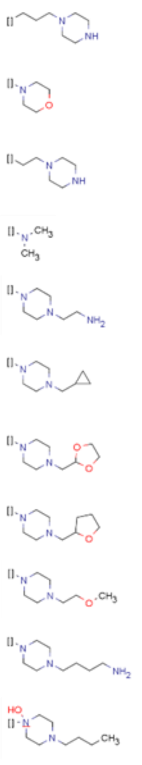

Compound	IC₅₀ [nM]
SC81452 *(616 Da)*	100
**SC81458** ***(628 Da)***	8
SC82342 *(628 Da)* (Para)	4,1
SC82365 *(600 Da)*	24,9
SC82367 *(614 Da)*	7
SC82486 *(614 Da)*	38,3
SC82487 *(670 Da)*	196
SC82508 *(614 Da)*	5,8
SC82682 *(586 Da)*	277
SC82833 *(642 Da)*	13,2
SC82904 *(572 Da)*	98,3
SC86669 *(641 Da)*	11,5
SC87409 *(628 Da)*	285
SC87507 *(573 Da)*	139
SC87508 *(600 Da)*	834
SC87860	626
SC87866 *(615 Da)*	263
SC87867 *(626 Da)*	10
SC87868 *(658 Da)*	37,3
SC87869 *(656 Da)*	53,9
SC87870 *(630 Da)*	30
SC88381 *(643 Da)*	2570
SC88464 *(644 Da)*	125

Acyl-piperazine chain / *Alkylating amine*

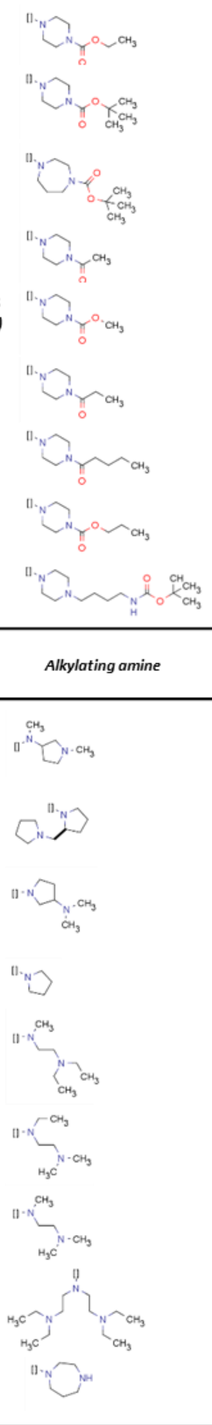

Compound	IC₅₀ [nM]
Acyl-piperazine chain	
SC82686 *(644 Da)*	4,4
SC82693 *(672 Da)*	606
SC82694 *(686 Da)*	859
SC83287 (614 Da)	25,4
**SC83288** ***(630 Da)***	3
SC83289 *(628 Da)*	3,8
SC83290 *(656 Da)*	24,8
SC83311 *(658 Da)*	11,6
SC87410 *(743 Da)*	743
Alkylating amine	
SC82683 *(600 Da)*	117
SC82684 *(640 Da)*	34
SC82689 *(600 Da)*	146
SC82832 *(557 Da)*	176
SC82869 *(616 Da)*	88,7
SC82870 *(602 Da)*	73,6
SC82871 *(588 Da)*	110
SC82872 *(701 Da)*	136
SC82905 *(586 Da)*	75,2

Polar groups

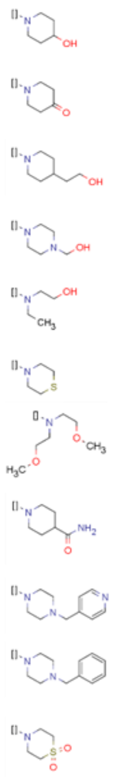

Compound	IC₅₀ [nM]
SC77225 *(587 Da)*	165
SC81450 *(585 Da)*	278
SC81452 *(616 Da)*	100
SC81453 *(602 Da)*	282
SC81454 *(575 Da)*	201
SC81455 *(589 Da)*	242
SC81456 *(619 Da)*	496
SC81457 *(614 Da)*	149
SC82687 *(663 Da)*	995
SC82692 *(662 Da)*	209
SC85039 *(621 Da)*	41,4

West-side optimization

Imine chain influence

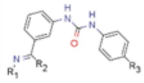

	R1	R2	R3	IC50 [nM]
SC88408 *(506 g mol-1)*	-OH	$-NH_2$	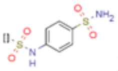	> 16 µM
SC89127 *(646 g mol-1)*	-OH	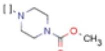	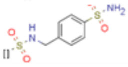	948
SC90436 *(672 g mol-1)*	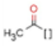	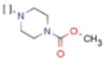	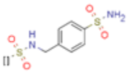	219
SC90478 *(756 g mol-1)*	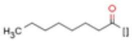	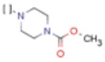	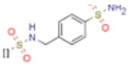	1910
SC90761 *(515 g mol-1)*	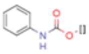	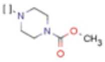	-H	308

Linker optimization

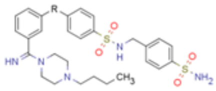

	R	IC50 [nM]
Diaryl urea linker		
SC83332 *(613 g mol-1)*	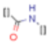	372
SC84185 *(627 g mol-1)*	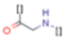	7160
SC84539 *(627 g mol-1)*	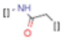	870
Middle N-alkyl linker		
SC85194 *(543 g mol-1)*	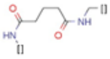	> 16 µM
SC85219 *(585 g mol-1)*	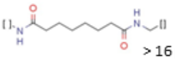	> 16 µM

Benzene linker		IC50 [nM]
SC86755 *(494 g mol-1)*	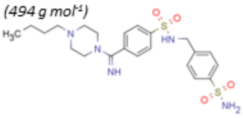	> 16 µM
SC86756 *(494 g mol-1)*	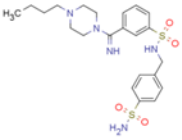	> 10 µM

West-side aryl substitution influence

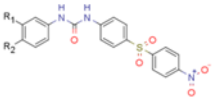

	R1	R2	IC50 [nM]
SC08323 *(439 g mol-1)*	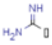	-H	226
SC08324 *(439 g mol-1)*	-H	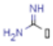	357
SC09457 *(526 g mol-1)*	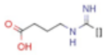	-H	> 16 µM
SC12599 *(466 g mol-1)*	-Cl	-Cl	4070
SC47789 *(510 g mol-1)*	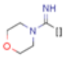	-H	88,7
SC47790 *(494 g mol-1)*	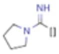	-H	61,9
SC47791 *(523 g mol-1)*	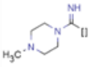	-H	122
SC47793 *(524 g mol-1)*	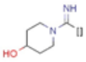	-H	31,8
SC47830 *(455 g mol-1)*	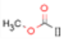	-H	> 16 µM
SC47946 *(440 g mol-1)*	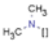	-H	6210
SC48019 *(497 g mol-1)*	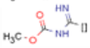	-H	1580
SC48020 *(512 g mol-1)*	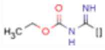	-H	750
SC48021 *(540 g mol-1)*	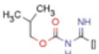	-H	1820
SC48022 *(540 g mol-1)*	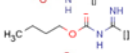	-H	1400
SC80504 *(525 g mol-1)*	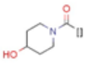	-H	7190
SC80548 *(524 g mol-1)*	-H	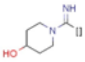	71,5
SC80613 *(440 g mol-1)*	-H	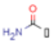	3350

Simultanous West- and East-side optimization

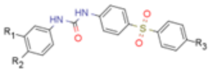

	R1	R2	R3	IC_{50} [nM]
SC48443 *(473 g mol-1)*	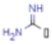	-H	-Br	63,9
SC77215 *(557 g mol-1)*	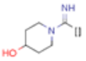	-H	-Br	67,9
SC80503 *(513 g mol-1)*	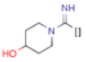	-H	-Cl	37,5
SC80549 *(430 g mol-1)*	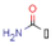	-H	-Cl	3270
SC80697 *(557 g mol-1)*	-H	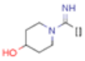	-Br	200
SC81468 *(571 g mol-1)*	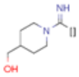	-H	-Br	1450
SC81471 *(589 g mol-1)*	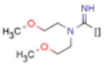	-H	-Br	2390
SC81472 *(560 g mol-1)*	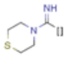	-H	-Br	51,2
SC81474 *(527 g mol-1)*	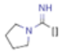	-H	-Br	47,8
SC81476 *(584 g mol-1)*	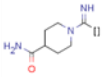	-H	-Br	41,1

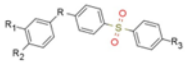

	R1	R2	R3	R	IC_{50} [nM]
SC81509 *(494 g mol-1)*	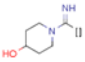	-H	$-NH_2$	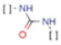	4170
SC08666 *(454 g mol-1)*	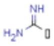	-H	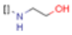	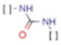	864
SC09187 *(482 g mol-1)*	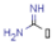	-H	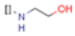	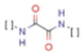	3470
SC48124 *(409 g mol-1)*	-H	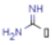	$-NH_2$	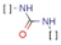	1020
SC48125 *(409 g mol-1)*	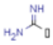	-H	$-NH_2$	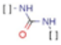	289

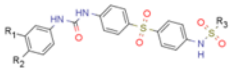

	R1	R2	R3	IC_{50} [nM]
SC48092 *(488 g mol-1)*	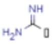	-H	$-CH_3$	3600
SC48129 *(569 g mol-1)*	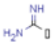	-H	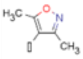	2200
SC48143 *(569 g mol-1)*	-H	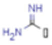	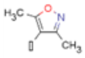	> 2560

Simultanous West- and East-side optimization

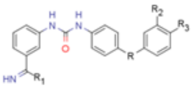

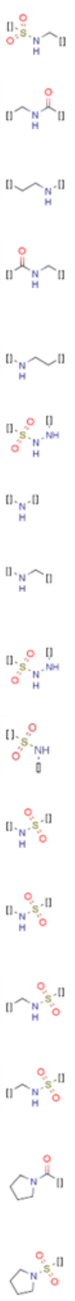

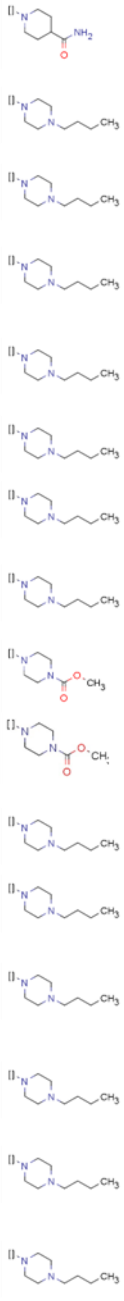

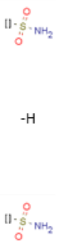

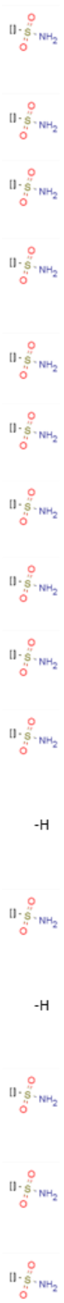

	R	R1	R2	R3	IC_{50} [nM]
SC81457 *(614 g mol⁻¹)*			-H		149
SC84129 *(592 g mol⁻¹)*			-H		1460
SC84199 *(578 g mol⁻¹)*			-H		1440
SC84302 *(592 g mol⁻¹)*			-H		2170
SC84472 *(578 g mol⁻¹)*			-H		2180
SC84509 *(629 g mol⁻¹)*			-H		1000
SC84952 *(550 g mol⁻¹)*			-H		4100
SC84969 *(564 g mol⁻¹)*			-H		2530
SC85036 *(631 g mol⁻¹)*			-H		5280
SC85955 *(616 g mol⁻¹)*			-H		4870
SC86099 *(613 g mol⁻¹)*				-H	1370
SC86100 *(613 g mol⁻¹)*			-H		439
SC86123 *(627 g mol⁻¹)*				-H	820
SC86124 *(627 g mol⁻¹)*			-H		448
SC88165 *(604 g mol⁻¹)*			-H		1560
SC88166 *(640 g mol⁻¹)*			-H		1600

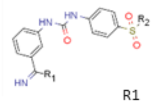

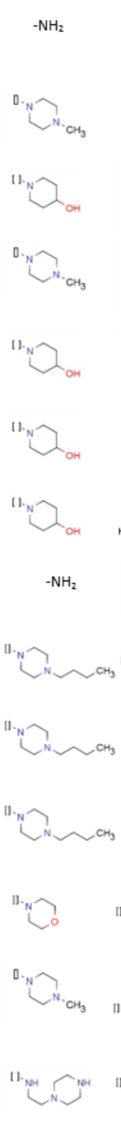

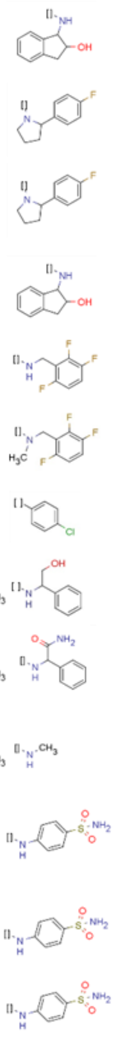

	R1	R2	IC_{50} [nM]
SC48590 *(403 g mol⁻¹)*	$-NH_2$		1020
SC50582 *(549 g mol⁻¹)*			> 16 µM
SC50603 *(566 g mol⁻¹)*			452
SC50604 *(565 g mol⁻¹)*			901
SC50633 *(550 g mol⁻¹)*			3820
SC77227 *(562 g mol⁻¹)*			244
SC80166 *(576 g mol⁻¹)*			14,6
SC80502 *(429 g mol⁻¹)*	$-NH_2$		77,5
SC85370 *(579 g mol⁻¹)*			2520
SC85371 *(592 g mol⁻¹)*			2900
SC86525 *(473 g mol⁻¹)*			2170
SC87510 *(559 g mol⁻¹)*			4600
SC87512 *(572 g mol⁻¹)*			> 16 µM
SC87513 *(601 g mol⁻¹)*			9130

Others

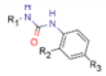	R_1	R_2	R_3	IC_{50} [nM]
SC08603 *(300 g mol^{-1})*	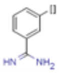	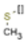	-H	> 16 µM
SC81373 *(441 g mol^{-1})*	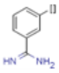	-H	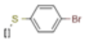	256
SC82748 *(448 g mol^{-1})*	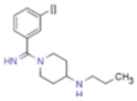	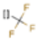	-H	1150
SC88407 *(439 g mol^{-1})*	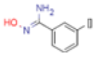	-H	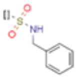	> 16 µM
SC90612 *(587 g mol^{-1})*	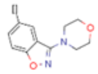	-H	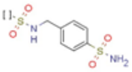	6170

Fragments

East-side fragments		IC_{50} [nM]
SC09367 *(186 g mol^{-1})*	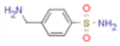	> 16µM
SC12215 *(341 g mol^{-1})*	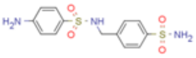	> 16µM
SC41565 *(157 g mol^{-1})*	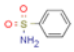	> 16µM
SC83244 *(383 g mol^{-1})*	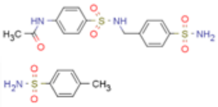	> 16µM
SC83448 *(171 g mol^{-1})*	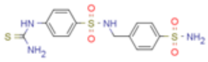	> 16µM
SC86438 *(400 g mol^{-1})*		> 16µM
**West-side fragment**		
SC66414 *(142 g mol^{-1})*	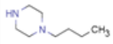	> 16µM

Supplementary Table 2: *In vitro* IC_{50}, solubility in water, and permeability of various SC compounds

Compound	*In vitro* IC_{50}	Solubility in H_2O	P_{app}
	nM	*$\mu g\ ml^{-1}$*	*$10^{-6}\ cm\ s^{-1}$*
amicarbalide	10	100	-
SC08165	134	n.d.	1.22
SC09064	10	316	0.28
SC43796	100	n.d.	0.13
SC48092	3600	320	n.d.
SC48125	120	100	0.2
SC48443	63.9	100	n.d.
SC77225	165	n.d.	0.38
SC81452	100	32	n.d.
SC81458	8	890	0.76
SC82367	7	>1000	5.40
SC82486	38	100	2.50
SC82487	196	>1000	n.d.
SC82687	995	32	n.d.
SC82833	13	100	n.d.
SC82869	89	320	n.d.
SC83287	5000	10	n.d.
SC83288	3	940	0.42
SC83289	1820	32	1.70
SC83290	40	>1000	n.d.
SC85343	1550	n.d.	1.75
SC87507	139	n.d.	1.23

n.d., not determined.

Supplementary Table 3: IC_{50} values of various *P. falciparum* strains

Strain	SC81458	SC83288	Chloroquine	Quinine	Quinidine	Mefloquine	Pyrimethamine	Amodiaquin	Artemisinin
	nM	*nM*	*nM*	*nM*	*nM*	*nM*	*nM*	*nM*	*nM*
Dd2	8 ± 1	3 ± 1	135 ± 21	100 ± 1	15 ± 1	4 ± 0.1	22 000 ± 1 700	7 ± 1	2 ± 0.1
D10	9 ± 4	5 ± 1	11 ± 3	58 ± 5	16 ± 1	1 ± 0.1	11 ± 2	7 ± 2	9 ± 0.4
3D7	13 ± 1	8 ± 2	3 ± 1	14 ± 2	14 ± 1	4 ± 0.1	16 ± 3	8 ± 1	8 ± 0.3
FCR3	13 ± 8	6 ± 1	25 ± 4	182 ± 27	32 ± 6	4 ± 0.6	31 ± 8	8 ± 0.4	5 ± 1
K1	9 ± 3	5 ± 1	70 ± 14	46 ± 4	12 ± 3	1 ± 0.2	> 15 000	9 ± 1	3 ± 1
Thai19	7 ± 1	4 ± 1	74 ± 17	62 ± 1	22 ± 5	1 ± 0.1	> 15 000	9 ± 1	3 ± 1
7G8	18 ± 5	10 ± 1	30 ± 7	70 ± 17	16 ± 4	2 ± 0.1	> 15 000	7 ± 2	1 ± 0.3
NF54	0.4 ± 0.05	2 ± 1	n.d.	n.d.	n.d.	n.d.	n.d.	n.d.	n.d.

The means ± of the standard error of the means of three independent determinations are shown.
n.d., not determined.

Supplementary Table 4: Kinome scan of SC81458

Kinase target	Remaining enzyme activity (%)	Kinase target	Remaining enzyme activity (%)	Kinase target	Remaining enzyme activity (%)
AAK1	100	CAMK4	51	EGFR(G719C)	80
ABL1	100	CAMKK1	93	EGFR(G719S)	100
ABL1(E255K)	100	CAMKK2	100	EGFR(L747-E749del, A750P)	86
ABL1(F317I)	100	CDC2L1	100	EGFR(L747-E752del, P753S)	84
ABL1(F317L)	100	CDC2L2	100	EGFR(L747-T751del, Sins)	69
ABL1(H396P)	100	CDK11	91	EGFR(L858R)	92
ABL1(M351T)	100	CDK2	100	EGFR(L858R, T790M)	100
ABL1(Q252H)	95	CDK3	100	EGFR(L861Q)	91
ABL1(T315I)	100	CDK5	100	EGFR(S752-I759del)	99
ABL1(Y253F)	92	CDK7	100	EPHA1	91
ABL2	77	CDK8	100	EPHA2	99
ACVR1	84	CDK9	100	EPHA3	100
ACVR1b	100	CDKL2	100	EPHA4	98
ACVR2a	100	CDKL3	98	EPHA5	100
ACVR2b	93	CDKL5	100	EPHA6	92
ACVRL1	72	CHEK1	98	EPHA7	81
ADCK3	100	CHEK2	100	EPHA8	83
ADCK4	100	CIT	94	EPHB1	84
AKT1	87	CLK1	100	EPHB2	100
AKT2	84	CLK2	100	EPHB3	98
AKT3	66	CLK3	100	EPHB4	100
ALK	100	CLK4	92	ERBB2	100
AMPK-alpha1	100	CSF1R	82	ERBB3	81
AMPK-alpha2	96	CSK	100	ERBB4	100
ANKK1	99	CSNK1a1l	100	ERK1	99
ARK5	92	CSNK1d	100	ERK2	80
ASK1	100	CSNK1e	100	ERK3	100
ASK2	100	CSNK1g1	100	ERK4	100
AURKA	68	CSNK1g2	100	ERK5	100
AURKB	78	CSNK1g3	100	ERK8	100
AURKC	73	CSNK2a1	62	ERN1	100
AXL	100	CSNK2a2	78	FAK	100
BIKE	85	CTK	100	FER	100
BLK	100	DAPK1	100	FES	100
BMPR1a	100	DAPK2	100	FGFR1	100
BMPR1b	85	DAPK3	100	FGFR2	100
BMPR2	100	DCAMKL1	100	FGFR3	100
BMX	100	DCAMKL2	100	FGFR3(G697C)	100
BRAF	95	DCAMKL3	62	FGFR4	100
BRAF(V600E)	100	DDR1	91	FGR	100
BRK	100	DDR2	100	FLT1	91
BRSK1	69	DLK	100	FLT3	100
BRSK2	100	DMPK	76	FLT3(D835H)	100
BTK	100	DMPK2	100	FLT3(D835Y)	89
CAMK1	82	DRAK1	98	FLT3(ITD)	85
CAMK1d	91	DRAK2	88	FLT3(K663Q)	93
CAMK1g	98	DYRK1a	100	FLT3(N841I)	100
CAMK2a	100	DYRK1b	87	FLT4	100
CAMK2b	100	DYRK2	100	FRK	100
CAMK2d	90	EGFR	100	FYN	100
CAMK2g	100	EGFR(E46-A750del)	96	GAK	92

Kinase target	Remaining enzyme activity (%)	Kinase target	Remaining enzyme activity (%)	Kinase target	Remaining enzyme activity (%)
GCN2(kin.Dom.2, S808G)	100	MAP3K4	100	OSR1	100
GRK1	100	MAP4K2	100	p38-alpha	88
GRK4	92	MAP4K3	95	p38-beta	100
GRK7	100	MAP4K4	100	p38-delta	89
GSK3a	100	MAP4K5	100	p38-gamma	85
GSK3b	100	MAPKAPK2	71	PAK1	100
HCK	100	MAPKAPK5	91	PAK2	100
HIPK1	100	MARK1	100	PAK3	93
HIPK2	100	MARK2	100	PAK4	100
HIPK3	99	MARK3	99	PAK6	95
HIPK4	100	MARK4	100	PAK7	100
HPK1	100	MAST1	46	PCTK1	92
HUNK	100	MEK1	100	PCTK2	97
ICK	73	MEK2	100	PCTk3	98
IGF1R	77	MEK3	100	PDGFRa	87
IKK-alpha	100	MEK4	100	PDGFRb	61
IKK-beta	100	MEK6	81	PDPK1	74
IKK-epsilon	100	MELK	100	PFTAIRE2	100
INSR	100	MERTK	100	PFTK1	100
INSRR	95	MET	78	PHKG1	80
IRAK1	95	MET(M1250T)	100	PHKG2	100
IRAK3	69	MET(Y1235D)	100	PIK3C2b	98
ITK	86	MINK	95	PIK3C2g	100
JAK1(JH1domain-catalytic)	87	MKNK1	71	PIK3CA	83
JAK1(JH2domain-pseudokinase)	89	MKNK2	100	PIK3CA(C420R)	100
JAK2(JH1domain-catalytic)	100	MLCK	100	PIK3CA(E542K)	100
JAK3(JH1domain-catalytic)	100	MLK1	64	PIK3CA(E545A)	100
JNK1	98	MLK2	100	PIK3CA(E545K)	100
JNK2	97	MLK3	100	PIK3CA(H1047L)	100
JNK3	100	MRCKA	100	PIK3CA(H1047Y)	100
KIT	60	MRCKB	99	PIK3CA(M1043I)	100
KIT(D816V)	100	MST1	100	PIK3CA(Q546K)	100
KIT(L576P)	66	MST1R	91	PIK3Cb	70
KIT(V559D)	100	MST2	100	PIK3Cd	73
KIT(V559D, T670I)	85	MST3	100	PIK3Cg	92
KIT(V559D, V654A)	100	MST4	100	PIK4Cb	100
LATS1	82	MUSK	95	PIM1	100
LATS2	100	MYLK	95	PIM2	87
LCK	100	MYLK2	100	PIM3	100
LIMK1	76	MYO3a	100	PIP5K1a	100
LIMK2	100	MYO3b	100	PIP5K2b	84
LKB1	88	NDR1	100	PKAC-apha	75
LOK	100	NDR2	100	PKAC-beta	78
LTK	100	NEK1	83	PKMYT1	90
LYN	97	NEK2	96	PKN1	100
LZK	100	NEK5	100	PKN2	67
MAK	91	NEK6	100	PLK1	96
MAP3K1	100	NEK7	95	PLK2	100
MAP3K15	100	NEK9	100	PLK3	75
MAP3K2	100	NIM1	100	PLK4	93
MAP3K3	100	NLK	95	PRKCD	100

Kinase target	Remaining enzyme activity (%)	Kinase target	Remaining enzyme activity (%)
PRKCE	100	SRPK1	100
PRKCH	92	SRPK2	93
PRKCQ	100	SRPK3	83
PRKD1	65	STK16	100
PRKD2	97	STK33	100
PRKD3	100	STK35	87
PRKG1	86	STK36	100
PRKG2	80	STK39	57
PRKR	91	SYK	100
PRKX	72	TAK1	100
PRP4	100	TAO1	100
PYK2	98	TAOK1	100
QSK	100	TAOK3	100
RAF1	100	TBK1	100
RET	89	TEC	90
RET(M918T)	100	TESK1	100
RRET(V804L)	100	TGFBR1	100
RET(V804M)	100	TGFBR2	80
RIOK1	75	TIE1	99
RIOK2	98	TIE2	100
RIOK3	77	TLK1	100
RIPK1	100	TLK2	97
RIPK2	96	TNIK	100
RIPK4	74	TNK1	70
ROCK1	100	TNK2	94
ROCK2	76	TNNI3K	100
ROS1	100	TRKa	100
RPS6KA1(Kin.Dom.1-N-term)	100	TRKb	100
RPS6KA1(Kin.Dom.2-C-term)	100	TRKc	100
RPS6KA2(Kin.Dom.1-N-term)	100	TSSK1B	97
RPS6KA2(Kin.Dom.2-C-term)	100	TTK	91
RPS6KA3(Kin.Dom.1-N-term)	100	TXK	87
RPS6KA4(Kin.Dom.1-N-term)	85	TYK2(JHdomain-catalytic)	76
RPS6KA4(Kin.Dom.2-C-term)	92	TYK2(JHdomain-speudokinase)	93
RPS6KA5(Kin.Dom.1-N-term)	96	TYRO3	79
RPS6KA5(Kin.Dom.2-C-term)	100	ULK1	100
RPS6KA6(Kin.Dom.1-N-term)	68	ULK2	100
RPS6KA6(Kin.Dom.2-C-term)	100	ULK3	73
SBK1	72	VEGFR2	84
SgK085	100	WEE1	100
SgK110	100	WEE2	100
SIK	97	YANK2	99
SIK2	100	YANK3	97
SLK	100	YES	97
SNARK	100	YSK1	76
SRC	81	YSK4	100
SRMS	79	ZAK	100
		ZAP70	89

Supplementary Table 5: Interaction of SC81458 and SC83288 with human transporters and receptors

Receptor/Transporter	Inhibition of control specific binding (%)		Control specific binding		
	SC81458	SC83288	compound	IC_{50} (M)	K_i (M)
A_1 (h)	7	8	DPCPX	8.2E-09	5.2E-09
A_{2A} (h)	5	13	NECA	3.7E-08	3.0E-08
A_3 (h)	0	0	IB-MECA	1.1E-09	6.4E-10
a_1 (non-selective)	21	9	prazosin	8.2E-10	2.2E-10
a_2 (non-selective)	21	14	yohimbine	6.9E-08	3.0E-08
b_1 (h)	-2	7	atenolol	2.8E-07	2.0E-07
b_2 (h)	6	27	ICI 118551	1.2E-09	4.0E-10
AT_1 (h)	11	6	saralasin	1.5E-09	7.6E-10
BZD (central)	16	70	diazepam	9.8E-09	8.3E-09
B_2 (h)	7	-4	NPC 567	1.8E-08	1.1E-08
CB_1 (h)	21	1	CP 55940	1.7E-09	1.5E-09
CCK_1 (CCK_A) (h)	0	-5	CCK-8s	2.0E-10	1.5E-10
D_1 (h)	51	79	SCH 23390	8.1E-10	3.2E-10
D_{2S} (h)	15	1	(+)butaclamol	1.0E-08	3.5E-09
ET_A (h)	1	0	endothelin-1	7.8E-11	3.9E-11
GABA (non-selective)	14	6	GABA	1.6E-08	9.5E-09
GAL_2 (h)	-15	4	galanin	1.1E-09	1.1E-09
CXCR2 (IL-8B) (h)	-8	-1	IL-8	6.3E-11	2.9E-11
CCR1 (h)	-13	-2	MIP-1a	6.0E-11	3.0E-11
H_1 (h)	6	8	pyrilamine	4.8E-09	1.7E-09
H_2 (h)	26	0	cimetidine	1.2E-07	1.2E-07
MC_4 (h)	-10	4	NDP-a-MSH	3.4E-10	3.1E-10
MT_1 (ML_{1A}) (h)	-5	-16	melatonin	1.6E-10	9.9E-11
M_1 (h)	36	75	pirenzepine	2.9E-08	2.5E-08
M_2 (h)	45	68	methoctramine	4.8E-08	3.3E-08
M_3 (h)	48	55	4-DAMP	9.6E-10	6.8E-10
NK_2 (h)	51	20	[$Nleu^{10}$]-NKA (4-10)	3.8E-09	2.1E-09
NK_3 (h)	6	5	SB 222200	1.6E-08	8.9E-09
Y_1 (h)	0	0	NPY	2.6E-10	1.8E-10
Y_2 (h)	-20	-4	NPY	1.3E-10	5.2E-11
NTS_1 (NT_1) (h)	1	1	neurotensin	1.1E-09	8.6E-10
d_2 (DOP) (h)	10	1	DPDPE	4.0E-09	2.4E-09
k (KOP)	43	36	U 50488	1.3E-09	8.9E-10
m (MOP) (h)	24	25	DAMGO	4.8E-10	2.0E-10
NOP (ORL1) (h)	2	10	nociceptin	9.5E-10	2.1E-10
TP (h) (TXA_2/PGH_2)	-10	-5	U 44069	1.4E-08	6.2E-09
5-HT_{1A} (h)	2	15	8-OH-DPAT	6.7E-10	4.2E-10
5-HT_{1B}	-3	6	serotonin	9.5E-09	5.8E-09
5-HT_{2A} (h)	17	30	ketanserin	3.3E-09	1.8E-09
5-HT_{2B} (h)	37	17	(±)DOI	5.1E-09	4.6E-09
5-HT_3 (h)	1	17	MDL 72222	6.9E-09	4.8E-09
5-HT_{5a} (h)	1	16	serotonin	4.2E-07	2.1E-07
5-HT_6 (h)	-2	11	serotonin	1.5E-07	7.3E-08
5-HT_7 (h)	19	4	serotonin	5.4E-10	2.0E-10
sst (non-selective)	-2	4	somatostatin-14	1.5E-10	9.2E-11
$VPAC_1$ (VIP_1) (h)	-4	-5	VIP	7.0E-11	3.9E-11
V_{1a} (h)	-1	11	[$d(CH_2)_5^1$,$Tyr(Me)_2$]-AVP	1.7E-09	1.0E-09
Ca^{2+} channel (L, verapamil site)	17	14	D 600	6.7E-08	3.4E-08
K_V channel	0	2	a-dendrotoxin	1.5E-10	1.2E-10
Na^+ channel (site 2)	45	19	veratridine	1.0E-05	9.0E-06
Cl^- channel (GABA-gated)	-28	-37	picrotoxinin	1.2E-07	1.0E-07
norepinephrine transporter (h)	73	59	protriptyline	5.5E-09	4.1E-09
dopamine transporter (h)	79	82	BTCP	1.1E-08	6.0E-09
5-HT transporter (h)	28	38	imipramine	4.2E-09	1.9E-09

Supplementary Table 6: Cytochromes P inhibitory potency of SC81458 and SC83288

Enzyme	Substrate	Metabolite	Remaining enzyme activity (%)	
			SC81458 (10 µM)	SC83288 (10 µM)
CYP1A2	melatonin	6-OH-melatonin	95	97
CYP2A6	coumarin	7-OH-coumarin	98	101
CYP2B6	bupropion	OH-bupropion	87	95
CYP2C8	amodiaquine	Desethylamodiaquine	95	89
CYP2C9	tolbutamide	OH-tolbutamide	104	103
CYP2C19a	omeprazole	5-OH-omeprazole	87	104
CYP2C19b	omeprazole	desmethylomeprazole	89	96
CYP2D6	dextromethorphan	O-desmethyldextromethorphan	107	91
CYP2E1	chlorzoxazone	6-OH-chlorzoxazone	106	68
CYP3A4a	midazolam	1'-OH-midazolam	74	92
CYP3A4b	testosterone	6β-OH-testosterone	77	88
CYP3A4c	omeprazole	omeprazole sulphone	66	87
CYP3A4d	omeprazole	3-OH-omeprazole	98	92

Supplementary Table 7: Clinical laboratory parameters in rats after treatment with SC83288

Clinical Biochemistry

		Reference Range		24h			
		**Lower limit**	**Upper limit**	**Placebo (n=3)**	**15 mg/kg (n=3)**	**22.5 mg/kg (n=2)**	**30 mg/kg (n=3)**
ALB	g dl^{-1}	3.8	5.0	4.8	4.9	5.0	5.4
ALB/GLOB	g dl^{-1}	2.2	6.3	8.2	5.5	6.7	8.9
ALP	U l^{-1}	224	627	470	306	399	424
ALT	U l^{-1}	37	79	57	51	58	58
AMY	U l^{-1}	685	1029	915	909	921	962
AST	U l^{-1}	59	115	72	70	79	85
BUN	mg dl^{-1}	8.3	18.8	14.3	14.7	17.5	15.7
BUN/CREA	mg dl^{-1}	20.0	90.8	64.4	66.7	72.5	78.3
CA	mg dl^{-1}	10.4	11.6	11.0	11.0	11.0	11.2
CK	U l^{-1}	71	355	87	176	97	91
CRE	mg dl^{-1}	0.2	0.5	0.2	0.2	0.3	0.2
GLOB	g dl^{-1}	0.8	1.8	0.7	0.9	0.8	0.6
GLU	mg dl^{-1}	151	262	165	202	160	170
K+	mmol l^{-1}	4.8	6.9	5.5	5.1	5.3	5.4
NA/K	mmol l^{-1}	19.6	28.5	24.7	26.7	25.7	25.2
NA+	mmol l^{-1}	132	141	136	136	135	136
PHOS	mg dl^{-1}	7.5	10.7	9.0	9.4	9.2	9.0
TBIL	mg dl^{-1}	0.1	0.4	0.3	0.4	0.3	0.3
TP	g dl^{-1}	5.2	6.0	5.5	5.8	5.7	6.0

Heamatology

		Reference Range		24h			
		**Lower limit**	**Upper limit**	**Placebo (n=3)**	**15 mg/kg (n=3)**	**22.5 mg/kg (n=2)**	**30 mg/kg (n=3)**
EOS	%	0.1	1.8	0.2	0.4	0.3	0.3
GRA	10^3 mm^{-3}	0.9	4.0	1.7	3.0	2.4	1.8
GRA	%	9.9	26.8	13.3	17.7	16.2	15.5
HCT	%	32.7	44.5	39.6	40.9	39.6	40.1
HGB	g dl^{-1}	12.5	15.1	14.3	14.3	14.2	14.3
LYM	10^3 mm^{-3}	5.3	15.3	9.8	13.4	11.7	9.3
LYM	%	71.4	89.1	85.4	80.7	82.1	83.0
MCH	pg	19.0	24.5	20.9	20.5	21.0	20.5
MCHC	g dl^{-1}	31.6	41.1	36.0	35.1	35.9	35.8
MCV	μm^3	56	64	58	58	59	57
MON	10^3 mm^{-3}	0.0	0.3	0.1	0.2	0.2	0.1
MON	%	0.9	2.1	1.3	1.6	1.8	1.5
MPV	μm^3	5.5	7.2	5.7	5.7	5.9	5.7
PLT	10^3 mm^{-3}	381	1049	821	812	847	869
RBC	10^3 mm^{-3}	5.42	7.33	6.82	7.00	6.77	7.00
RDW	%	11.5	14.2	13.3	13.3	13.3	13.4
WBC	10^3 mm^{-3}	6.4	19.8	11.6	16.7	14.3	11.2

Supplementary Table 8: Activity of various SC compounds in mouse malaria model systems

Compound	*In vitro* IC_{50}	Model organism	Dose	Administrative route	Outcome
	nM		*mg kg^{-1}*		
SC08165	450	*P. berghei*	50	i.p 1x4 days	inactive
SC09064	10	*P. berghei*	50	i.p. 1x4 days	inactive
SC09064	10	*P. berghei*	10	i.v. 1x4 days	inactive
SC09064	10	*P. chabaudi*	10	i.v. 1x4 days	inactive
SC09064	10	*P. vinckei*	30	i.p. 1x4 days	inactive
SC09823	390	*P. berghei*	50	i.p. 1x4 days	inactive
SC77215	68	*P. vinckei*	20	i.p. 1x4 days	45% decrease of parasitemia
SC81458	8	*P. berghei*	30	i.p. 1x4 days	inactive
SC81458	8	*P. vinckei*	30	i.p. 1x4 days	> 90% decrease of parasitemia, rescrudescence
SC82904	99	*P. vinckei*	30	i.p. 1x4 days	> 80 % decrease of parasitemia, no complete clearance
SC83288	3	*P. berghei*	30	i.p. 1x4 days	inactive
SC83288	3	*P. vinckei*	20	i.p. 1x4 days	complete clearance, no recrudescence
SC83290	30	*P. vinckei*	30	i.p. 1x4 days	>90% decrease of parasitemia, no complete clearance

Supplementary Table 9: Plasma protein binding of SC83288

Compound	Bound fraction (%)			
	human	rat	mouse	cynomolgus
SC83288	78 ± 1.3	80 ± 0.6	89 ± 2.2	82 ± 2.8

The means ± standard error of the means of three independent determinations are shown.

Supplementary Table 10: Summary of sequencing data and variant calling

Internal ID	Description	Sample ID	Read Accession number	Read Accession number	Reads (number)	Mapped reads (%)	Fold coverage	NP (number)	Indels (number)
PfPar	Parent	ERS445938	ERR573917	ERR571272	7,317,338	92.8	46.3	3,210	4,304
Pfcl4_1		ERS445939	ERR573918	ERR571273	8,050,856	93.2	51.2	4,829	4,799
Pfcl15		ERS445940	ERR573919	ERR571274	6,852,992	92.7	43.3	3,839	4,416
Pfcl21		ERS445941	ERR573920	ERR571275	7,494,362	91.8	46.9	3,816	4,397
Pfcl18		ERS445942	ERR573921	ERR571276	5,737,904	92.6	36.2	3,072	4,052
Pfcl13		ERS445943	ERR573922	ERR571277	6,506,440	92.4	41.0	3,492	4,240
Pfcl1		ERS445944	ERR573923	ERR571278	4,721,080	92.0	29.6	3,023	3,732
Pfcl9		ERS445945	ERR573924	ERR571279	6,134,674	92.3	38.6	3,218	4,167
Pfcl1b		ERS445946	ERR573925	ERR571280	6,833,770	93.0	43.3	3,552	4,443

Each sample was run twice.

Supplementary Table 11: IC_{50} values of resistant strains to licensed drugs

Drug	Dd2 SC81458 resistant	Dd2 SC83288 resistant	Dd2
	nM	*nM*	*nM*
Chloroquine	150 ± 12	120 ± 25	135 ± 21
Quinine	164 ± 10	94 ± 9	100 ± 10
Quinidine	26 ± 1	9 ± 0.4	15 ± 1
Mefloquine	18 ± 1	6 ± 0.4	4 ± 0.1
Pyrimethamine	31 000 ± 2000	22 000 ± 2000	22 000 ± 2000
Amodiaquine	8 ± 3	5 ± 1	7 ± 1
Artemisinine	4 ± 1	1 ± 0.2	2 ± 0.1
Atovaquone	0.3 ± 0.1	0.8 ± 0.1	0.6 ± 0.1

The means ± of the standard error of the means of five independent determinations are shown.

Supplementary Note 1

Analytical HPLC-MS determinations were perfomed using Waters 2700 Autosampler, Waters 1525 Multisolvent Delivery System and Micromass ZQ single quadrupole mass spectrometer with electrospray source. Column: Chromolith Fast Gradient C18 (Merck), 50 x 2 mm, with stainless steel 2 µm prefilter. Eluent A, H_2O + 0.1% HCOOH; eluent B, MeCN. The $[M+H]^+$ data is reported below. Preparative HPLC-MS were performed with a Waters 2700 Autosampler, Waters 600 Multisolvent Delivery System with preparative pump heads (500 µL), Waters 600S Controller and Waters ZQ single quadrupole mass spectrometer with electrospray source. Column: Waters X-Terra RP18, 5 µm, 19 x 150 mm. Eluent A, H_2O + 0.1% HCOOH; eluent B, MeCN. Different linear gradients, individually adapted to sample. ^{1}H-NMR and ^{13}C-NMR were performed with a Bruker AV300 (300.13 Mhz) at temperature of 305 K. Abbreviations used for the peak identification were: s = singlet; d = doublet; t = triplet; q = quartet; m = multiplet; J = ^{1}H-^{1}H coupling constant.

Lead-optimization campaign – Synthesis of the “focussed-library”

All the compounds were obtained via solution synthesis methodologies. After work-up of the reactions, the compounds were purified by preparative HPLC/MS and characterized by LC/MS and in the case enough material was obtained, the corresponding compounds were further characterized by ^{1}H NMR. If the compounds were commercially available (Fisher Scientific, Acros Organics, Sigma-Aldrich, Maybridge Chemical), no characterization was performed.

The analytical analysis of the compounds listed in Supplementary Table 1 is provided below.

SC09064: $[M+H]^+$ calcd. for $C_{21}H_{22}N_6O_5S_2$, 503.117; found for $[M+H]^+$: 503.1. ^{1}HNMR (300 MHz, DMSO-d6) δ = 4.04 (s, 2 H), 7.21-7.33 (bm, 1 H), 7.38 (d, J = 8.0 Hz, 1 H), 7.44 (d, J = 8.5 Hz, 2 H), 7.55 (t, J = 7.9 Hz, 1 H), 7.66-7.76 (m, 6 H), 7.98-7.99 (m, 1 H), 8.01-8.17 (bm, 1 H), 8.92-9.27 (bm, 3 H), 9.39 (s, 1 H), 9.50 (s, 1 H). ^{13}CNMR (75 MHz, DMSO-d6) δ = 45.55, 117.13 (2×), 117.57, 121.28, 122.47, 125.56 (2×), 127.81 (4×), 129.40, 130.39, 132.88, 139.96, 142.02, 142.91, 143.43, 152.46, 165.68. **SC08165:** $[M+H]^+$ calcd. for $C_{21}H_{21}N_5O_3S$, 424.145; found for $[M+H]^+$: 424.1. **SC09231:** $[M+H]^+$ calcd. for $C_{21}H_{18}F_3N_5O_3S$, 478.116; found for $[M+H]^+$: 478.1. ^{1}H NMR (DMSO-*d$_6$*): 9.81-8.96 (m, 5H), 8.08 (t, *J*=5.7Hz, 1H), 7.97 (t, *J*=1.7Hz, 1H), 7.75-7.61 (m, 5H), 7.54 (t, *J*=8.0Hz, 1H), 7.46-7.35 (m, 2H), 7.05 (ddt, J_{HF}=9.2Hz, J_{HH}=9.2Hz, J_{HF}=2.2Hz, J_{HF}=3.8Hz, 1H), 4.03 (d, *J*=4.7Hz, 2H).**SC43796:** $[M+H]^+$ calcd. for $C_{23}H_{23}N_5O_4S$, 466.155; found for $[M+H]^+$: 466.1. ^{1}H NMR (DMSO-*d$_6$*): 10.40 (s, 1H), 10.21 (s, 1H), 7.99-7.92 (m, 1H), 7.85 (d, *J* = 8.8 Hz, 2H), 7.75 (d, *J* = 8.2 Hz, 1H), 7.67 (d, *J* = 8.8 Hz, 2H), 7.52 (t, *J* = 8.0 Hz, 1H), 7.39 (d, *J* = 7.9 Hz, 1H), 7.19-7.05 (m, 3H), 6.95 (d, *J* = 7.1 Hz, 1H), 5.01-4.94 (m, 1H), 4.57 (d, *J* = 4.7 Hz, 1H), 4.16-4.07 (m, 1H), 2.98-2.65 (m, 2H). (4H overlapped). **SC80166:** $[M+H]^+$ calcd. for $C_{27}H_{28}F_3N_5O_4S$, 576.188; found for $[M+H]^+$: 576.2. **SC81475**: $[M+H]^+$ calcd. for $C_{28}H_{32}BrN_5O_3S$, 599.148; found for $[M+H]^+$:

599.1. **SC82341:** $[M+H]^+$ calcd. for $C_{29}H_{33}F_3N_6O_3S$, 603.236; found for $[M+H]^+$: 603.2. ^1H NMR (DMSO-d_6): 11.00 (broad, 1H, NH); 10.97 (broad, 1H, NH); 8.03 (broad, 1H, NH); 7.83 (m, 1H, Ar-H); 7.74-7.63 (m, 5H, Ar-H); 7.51-7.33 (m, 2H, Ar-H); 7.13-7.00 (m, 2H, Ar-H); 4.02 (broad, 2H, CH_2); water signal partially covers a group signals; 2.33 (m, 2H, CH_2); 1.46-1.23 (m, 4H, $2CH_2$); 0.88 (t, 3H, J=7.2 Hz, CH_3). **SC82343:** $[M+H]^+$ calcd. for $C_{29}H_{36}N_6O_3S$, 549.263; found for $[M+H]^+$: 549.2. ^1H NMR (DMSO-d_6): 11.28-11.14 (broad, 2H, 2NH); 8.52 (s broad, 1H, NH); 7.94-7.87 (broad, 1H, NH); 7.83 (m, 1H, Ar-H); 7.75-7.67 (m, 5H, Ar-H); 7.47 (m, 1H, Ar-H); 7.32-7.19 (m, 5H, Ar-H); 7.11-7.07 (m, 1H, Ar-H); 3.95 (s broad, 2H, CH_2); water signal partially covers a group signals; 2.33 (m, 2H, CH_2); 1.46-1.23 (m, 4H, $2CH_2$); 0.88 (t, 3H, J=7.2 Hz, CH_3). **SC82746:** $[M+H]^+$ calcd. for $C_{26}H_{36}N_6O_4S$, 529.260; found for $[M+H]^+$: 529.2. ^1H NMR (DMSO-d_6): 9.83-9.13 (broad, 3H, 3NH); 7.90 (broad, 1H, Ar-H); 7.80-7.56 (m, 6H, Ar-H); 7.28-7.22 (m, 1H, Ar-H); 3.67 (m, 4H, $2CH_2$); 2.89 (m, 4H, $2CH_2$); water signal partially covers a group signals; 1.60-1.42 (broad, 2H, CH_2); 1.41-1.28 (m, 2H, CH_2); 0.93 (t, 3H, J=7.2 Hz, CH_3). **SC82893:** $[M+H]^+$ calcd. for $C_{26}H_{38}N_6O_5S$, 547.270; found for $[M+H]^+$: 547.2. ^1H NMR (DMSO-d_6):9.81 (broad, 2H, NH); 9.48 (broad, 1H, NH); 7.97 (broad, 1H, Ar-H); 7.79-7.57 (m, 6H, Ar-H); 7.29-7.24 (m, 1H, Ar-H); 3.55 (t, 4H, J=6.3 Hz, $2CH_2$); 3.18 (t, 4H, J=6.3 Hz, $2CH_2$); water signal partially covers a group signals; 1.68-1.56 (broad, 2H, CH_2); 1.43-1.31 (m, 2H, CH_2); 0.95 (t, 3H, J=7.3 Hz, CH_3). **SC82894:** $[M+H]^+$ calcd. for $C_{17}H_{21}N_5O_3S$, 376.144; found for $[M+H]^+$: 376.1. ^1H NMR (DMSO-d_6): 9.52-8.88 (m, 5H), 7.98 (t, *J*=1.7Hz, 1H), 7.73-7.64 (m, 5H), 7.54 (t, *J*=8.0Hz, 1H), 7.41-7.35 (m, 2H), 2.68 (dd, *J*=7.0Hz, *J*=13.0Hz, 2H), 1.43-1.31 (m, 2H), 0.79 (t, *J*=7.4Hz, 3H). **SC82895:** $[M+H]^+$ calcd. for $C_{25}H_{36}N_6O_3S$, 501.264; found for $[M+H]^+$: 501.3. ^1H NMR (DMSO-d_6): 9.77-9.27 (m broad, 3H, NH); 7.86 (broad, 1H, Ar-H); 7.66-7.46 (m, 6H, NH+Ar-H); 7.32 (m, 1H, Ar-H); 7.18-7.13 (m, 1H, Ar-H); water signal partially covers a group signals; 2.65-2.57 (m, 2H, CH_2); 1.58-1.44 (broad, 2H, CH_2); 1.36-1.20 (m, 4H, $2CH_2$); 0.84 (t, 3H, J=7.3 Hz, CH_3); 0.72 (t, 3H, J=7.4 Hz, CH_3). **SC85259:** $[M+H]^+$ calcd. for $C_{30}H_{39}N_7O_5S_2$, 642.253; found for $[M+H]^+$: 642.2. ^1HNMR (DMSO-d_6): 11.25 (s, 2H), 8.50 (s, 1H), 8.23-7.96 (m, 2H), 7.88-7.80 (m, 1H), 7.79-7.56 (m, 8H), 7.50 (t, *J* = 7.94 Hz, 1H), 7.41 (d, *J* = 8.4 Hz, 2H), 7.37-7.21 (m, 2H), 7.12 (d, *J* = 7.6 Hz, 1H), 4.43-4.27 (m, 1H), under water peak (6H), 2.40-2.22 (m, 2H), 1.49-1.24 (m, 4H), 1.20 (d, *J* = 6.9 Hz, 3H), 0.88 (t, *J* = 7.23, 7.23 Hz, 3H). ^{13}CNMR (75 MHz, DMSO-d_6): 13.81, 19.95, 23.14, 28.23, 43.68, 51.76 (2×), 52.28 (2×), 56.77, 117.01, 117.39 (2×), 120.73, 121.19, 152.52 (2×), 126.51 (2×), 127.50 (2×), 129.64, 129.81, 133.17, 141.13, 142.58, 143.97, 147.76, 152.97, 163.94, 167.01 ($HCOO^-$). **SC85343:** $[M+H]^+$ calcd. for $C_{30}H_{38}N_6O_4S$, 579.275; found for $[M+H]^+$: 579.3. ^1H NMR (DMSO-d_6): 10.54 (bs, 2H), 8.45 (s, 1H), 7.89 (d, *J*=8.4Hz, 1H), 7.83-7.80 (m, 1H), 7.68 (d, *J*=8.7Hz, 1H), 7.58-7.48 (m, 5H), 7.23-7.10 (m, 5H), 4.77 (bs, 1H), 4.20 (dd, *J*=7.0Hz, *J*=13.6Hz, 1H), 3.63-3.16 (m, 11H) under water peak, 2.36-2.31 (m, 2H), 1.47-1.24 (m, 4H), 0.88 (t, *J*=7.2Hz, 3H). **SC86048:** $[M+H]^+$ calcd. for $C_{30}H_{37}N_7O_5S_2$, 640.237; found for $[M+H]^+$: 640.2. ^1H NMR (DMSO-d_6):

11.37-11.17 (broad, 2H, 2NH), 7.81-7.87 (m, 7H, Ar-H); 7.50-7.41 (m, 2H, Ar-H); 7.28 (broad, 2H, NH_2); 7.12-7.09 (m, 1H, Ar-H); 4.64-4.59 (m, 4H, $2CH_2$); water signal partially covers a group signals; 2.33 (m, 2H, CH_2); 1.45-1.22 (m, 4H, $2CH_2$); 0.88 (t, 3H, J=7.2 Hz, CH_3). **SC86610:** $[M+H]^+$ calcd. for $C_{30}H_{36}F_2N_6O_5S_2$, 664.302; found for $[M+H]^+$: 664.2. ^1H NMR (DMSO-d_6): 11.43 (bs, 2H), 8.43 (s, 1H), 8.12-7.98 (bm, 1H), 7.88-7.83 (m, 1H), 7.78-7.65 (m, 6H), 7.50 (t, *J*=7.9Hz, 1H), 7.19-7.10 (m, 2H), 4.02 (bs, 2H), 3.76-3.30 (m, 8H) under water peak, 2.36-2.25 (m, 2H), 1.45-1.24 (m, 4H), 0.91-0.85 (m, 3H). **SC86714:** $[M+H]^+$ calcd. for $C_{29}H_{36}FN_7O_5S_2$, 646.223; found for $[M+H]^+$: 646.2. ^1H NMR (DMSO-d_6): 10.55-10.46 (m, 2H), 8.32-8.31 (m, 1H), 8.16-8.09 (m, 1H), 7.93 (dd, *J*=2.3Hz, *J*=7.0Hz, 1H), 7.82 (bs, 1H), 7.79-7.65 (m, 6H), 7.52 (t, *J*=7.9Hz, 1H), 7.45-7.30 (m, 3H), 7.16 (d, *J*=7.5Hz, 1H), 4.03-4.02 (m, 2H), 3.68-3.11 (m, 8H) under water peak, 2.36-2.32 (m, 2H), 1.47-1.23 (m, 4H), 0.88 (t, *J*=7.2Hz, 3H). **SC87211:** $[M+H]^+$ calcd. for $C_{27}H_{35}N_7O_5S_3$, 634.194; found for $[M+H]^+$: 634.1. ^1H NMR (DMSO-d_6): 11.22 (s, 1H, 1NH); 11.17 (s broad, 1H, 1NH); 8.23 (broad, 1H, NH); 7.84 (m broad, 1H, Ar-H); 7.76-7.47 (m, 8H, NH_2+6 Ar-H); 7.33 (d, 1H, J=3.7 Hz, CH); 7.14-7.10 (m, 1H, Ar-H); 6.91 (d, 1H, J=3.7 Hz, CH); 4.17 (broad, 2H, CH_2); 3.55 (broad, 4H, $2CH_2$); DMSO signal covers a group of signals; 2.34 (t, 2H, J=7.2 Hz, CH_2); 1.46-1.23 (m, 4H, $2CH_2$); 0.88 (t, 3H, J=7.2 Hz, CH_3). **SC86354:** $[M+H]^+$ calcd. for $C_{31}H_{35}N_7O_3S_2$, 618.232; found for $[M+H]^+$: 618.2. ^1H NMR (DMSO-d_6): 9.77-9.36 (m, 4H), 8.29 (s, 1H), 8.23 (d, *J*=8.6Hz, 2H), 8.03-7.89 (m, 4H), 7.71-7.53 (m, 4H), 7.39 (bs, 2H), 7.22 (d, *J*=7.5Hz, 1H), 3.66-3.17 (m, 10H) under water peak, 1.63-1.50 (bm, 2H), 1.33 (dd, *J*=7.4Hz, *J*=14.7Hz, 2H), 0.91 (t, *J*=7.2Hz, 3H). **SC86943:** $[M+H]^+$ calcd. for $C_{29}H_{33}N_7O_3S_2$, 592.216; found for $[M+H]^+$: 592.2. ^1H NMR (DMSO-d_6): 8.28 (bs, 1H), 8.25 (d, *J*=1.6Hz, 1H), 7.95 (bs, 1H), 7.74 (dd, *J*=1.8Hz, *J*=8.4Hz, 2H), 7.63 (d, *J*=8.0Hz, 1H), 7.51 (t, *J*=7.9Hz, 1H), 7.21 (bs, 2H), 7.14 (d, *J*=7.5Hz, 1H), 3.69-3.19 (m, 8H) under water peak, 2.38-2.31 (m, 2H), 1.48-1.22 (m, 4H), 0.88 (t, *J*=7.2Hz, 3H). **SC81458:** $[M+H]^+$ calcd. for $C_{29}H_{37}N_7O_5S_2$, 628.237; found for $[M+H]^+$: 628.1. ^{1}HNMR (300 MHz, DMSO-d_6): 11.19-11.29 (m, 2 H), 8.53 (s, 1 H), 7.99-8.12 (m, 1 H), 7.83-7.86 (m, 1 H), 7.69-7.76 (m, 8 H), 7.50 (t, *J* = 7.9 Hz, 1 H), 7.44 (d, *J* = 8.3 Hz, 2 H), 7.27-7.36 (m, 2 H), 7.13 (d, *J* = 7.6 Hz, 1 H), 4.03 (bs, 2 H), 3.52-3.60 (bm, 4 H), 2.50 (m, 4 H under DMSO peak), 2.32-2.36 (m, 2 H), 1.35-1.46 (m, 2 H), 1.24-1.33 (m, 2 H), 0.88 (t, *J* = 7.2 Hz, 3 H). ^{13}CNMR (75 MHz, DMSO-d_6): 13.81, 19.95, 28.23, 45.56 (3×), 51.76 (2×), 56.78, 116.99, 117.57 (2×), 120.75, 121.18, 125.55 (2×), 127.66 (2×), 127.81 (2×), 129.64, 129.85, 132.42, 141.09, 142.02, 142.91, 144.17, 152.94, 163.94, 167.24 ($HCOO^-$). **SC82342:** $[M+H]^+$ calcd. for $C_{29}H_{37}N_7O_5S_2$, 628.307; found for $[M+H]^+$: 628.1. **SC82365:** $[M+H]^+$ calcd. for $C_{27}H_{33}N_7O_5S_2$, 600.201; found for $[M+H]^+$: 600.1. ^1H NMR (DMSO-d_6):10.57-10.31 (m, 1H), 10.12 (bs, 1H), 8.30-8.09 (m, 1H), 7.91-7.49 (m, 11H), 7.43 (d, *J* = 8.14 Hz, 2H), 7.32 (s, 2H), 4.01 (d, *J* = 6.07 Hz, 2H), under water peak (10), 1.16 (dd, *J* = 6.07 Hz, 3H). **SC82367:** $[M+H]^+$ calcd. for $C_{28}H_{35}N_7O_5S_2$, 614.222; found for $[M+H]^+$: 614.1. ^1H NMR (DMSO-d_6): 9.80 (s broad, 1H, NH); 9.75 (s broad, 1H, NH); 9.70 (s broad,

1H, NH); 9.45 (s broad, 1H, NH); 8.08 (t, 1H, J=6.3 Hz, NH); 7.93 (m, 1H, Ar-H); 7.76-7.62 (m, 7H, Ar-H); 7.56 (m, 1H, Ar-H); 7.45-7.41 (m, 2H, Ar-H); 7.28 (s broad, 2H, NH_2); 7.25-7.21 (m, 1H, Ar-H); 4.04 (d, 2H, J=6.3 Hz, CH_2); water signal partially covers a group signals; 1.70-1.58 (m broad, 2H, CH_2); 0.92 (t, 3H, J=7.4 Hz, CH_3). **SC82486:** $[M+H]^+$ calcd. for $C_{28}H_{35}N_7O_5S_2$, 614.222; found for $[M+H]^+$: 614.2. ^1H NMR (DMSO-d_6): 10.14 (s 1H), 10.07 (s, 1H), 9.49 (bs, 1H), 8.17 (bs, 1H), 8.08 (t, *J*=6.3Hz, 1H), 7.78-7.72 (m, 5H), 7.70-7.64 (m, 3H), 7.53 (t, *J*=7.9Hz, 1H), 7.44 (d, *J*=8.4Hz, 2H), 7.29 (bs, 1H), 7.18 (d, *J*=7.7Hz, 1H), 4.03 (d, *J*=6.2Hz, 2H), 3.51-3.12 (m, 4H), 2.78-2.57 (m, 5H), 0.99 (d, *J*=6.5Hz, 6H). **SC82487:** $[M+H]^+$ calcd. for $C_{32}H_{43}N_7O_5S_2$, 670.284; found for $[M+H]^+$: 670.1 ^1H NMR (DMSO-d_6): 11.58-11.40 (m, 2H), 8.50 (s, 1H), 7.89-7.82 (m, 1H), 7.80-7.66 (m, 9H), 7.53-7.39 (m, 3H), 7.36-7.25 (m, 1H), 7.11 (d, *J* = 7.7 Hz, 1H), 4.02 (bs, 2H), under water peak (6H), 2.40-2.19 (m, 4H), 1.50-1.35 (m, 2H), 1.33-1.18 (m, 8H), 0.86 (t, *J* = 6.7 Hz, 3H). **SC82508:** $[M+H]^+$ calcd. for $C_{28}H_{33}N_7O_5S_2$, 612.206; found for $[M+H]^+$: 612.1. **SC82682:** $[M+H]^+$ calcd. for $C_{26}H_{31}N_7O_5S_2$, 586.190; found for $[M+H]^+$: 586.1. ^1H NMR (DMSO-d_6): 9.79 (s broad, 1H, NH); 9.66 (s broad, 1H, NH); 9.61 (broad, 1H, NH); 9.44 (s broad, 1H, NH); 8.09 (t, 1H, J=6.3 Hz, NH); 7.93 (m, 1H, Ar-H); 7.76-7.54 (m, 8H, Ar-H); 7.45-7.41 (m, 2H, Ar-H); 7.29 (s broad, 2H, NH_2); 7.24-7.21 (m, 1H, Ar-H); 4.04 (d, 2H, J=6.2 Hz, CH_2); water signal partially covers a group of signals. **SC82833:** $[M+H]^+$ calcd. for $C_{30}H_{39}N_7O_5S_2$, 642.253; found for $[M+H]^+$: 642.1. ^1H NMR (DMSO-d_6): 9.75 (very broad, 1H, NH); 9.54 (broad, 1H, NH); 9.50 (broad, 1H, NH); 9.40 (very broad, 1H, NH); 8.08 (t, 1H, J=6.3 Hz, NH); 7.92 (broad, 1H, Ar-H); 7.76-7.53 (m, 8H, Ar-H); 7.44-7.40 (m, 2H, Ar-H); 7.27 (broad, 2H, NH_2); 7.25-7.20 (m, 1H, Ar-H); 4.03 (d, 2H, J=6.3 Hz, CH_2); water signal partially covers a group signals; 1.65-1.53 (broad, 2H, CH_2); 1.35-1.24 (m, 4H, $2CH_2$); 0.88 (t, 3H, J=6.8 Hz, CH_3). **SC82904:** $[M+H]^+$ calcd. for $C_{25}H_{29}N_7O_5S_2$, 572.175; found for $[M+H]^+$: 572.1. ^1H NMR (DMSO-d_6): 9.83-9.41 (very broad, 4H, 4NH); 8.09 (t, 1H, J=6.4 Hz, NH); 7.93 (broad, 1H, Ar-H); 7.76-7.53 (m, 8H, Ar-H); 7.45-7.41 (m, 2H, Ar-H); 7.29 (s broad, 2H, NH_2); 7.27-7.23 (m, 1H, Ar-H); 4.04 (d, 2H, J=6.3 Hz, CH_2); 3.95 (m broad, 2H, CH_2); 3.58 (m broad, 2H, CH_2); water signal partially covers a group of signals. **SC86669:** $[M+H]^+$ calcd. for $C_{29}H_{35}N_7O_6S_2$, 642.217; found for $[M+H]^+$: 642.1. ^1H NMR (DMSO-d_6): 10.59 (very broad, 2H, 2NH); 8.06 (t broad, 1H, NH); 7.85-7.82 (m broad, 1H, Ar-H); 7.77-7.66 (m, 7H, Ar-H); 7.54-7.41 (m, 3H, Ar-H); 7.29 (s broad, 2H, NH_2); 7.18-7.13 (m, 1H, Ar-H); 4.16 (broad, 1H, CO-CH_2-N); 4.03 (d broad, 2H, CH_2); 3.68-3.44 (broad, 4H, $2CH_2$); 3.35 (t, 2H; J=7.2 Hz, CH2); water signal covers a group of signals; 1.54-1.21 (m, 4H, $2CH_2$); 0.90 (t, 3H; J=7.3 Hz, CH_3). **SC87409:** $[M+H]^+$ calcd. for $C_{29}H_{38}N_8O_5S_2$, 643.248; found for $[M+H]^+$: 643.2. ^1H NMR (DMSO-d_6): 10.16 (m, 1H), 10.03 (m, 1H), 9.80 (bs, 1H), 9.47 (bs, 1H), 9.10 (bs, 1H), 8.13-8.04 (m, 1H), 7.93-7.87 (m, 1H), 7.79-7.71 (m, 5H), 7.66 (d, *J*=8.9Hz, 2H), 7.53 (t, *J*=8.0Hz, 1H), 7.44 (d, *J*=8.3Hz, 2H), 7.35-7.27 (m, 3H), 4.03 (d, *J*=6.3Hz, 2H), 3.48-3.16 (m, 12H) under water peak, 1.76-1.55 (m, 4H). **SC87507:** $[M+H]^+$ calcd. for $C_{25}H_{28}N_6O_6S_2$, 573.159; found for $[M+H]^+$: 573.1. ^1H NMR

(DMSO-d_6): 9.81 (s broad, 1H, NH); 9.74 (s broad, 1H, NH); 9.37 (broad, 1H, NH); 8.07 (t, 1H, J=6.2 Hz, NH); 7.85 (m, 1H, Ar-H); 7.76-7.62 (m, 7H, Ar-H); 7.55-7.42 (m, 3H, Ar-H); 7.28 (s broad, 2H, NH_2); 7.28-7.18 (m, 3H, NH_2+Ar-H); 4.03 (d, 2H, J=6.0 Hz, CH_2); 3.74-3.72 (m broad, 4H, 2CH_2); 3.54-3.58 (m broad, 4H, 2CH_2). ^{13}C NMR (75 MHz, DMSO-d_6): 45.44, 65.01 (2×), 117.29, 117.63 (2×), 121.28, 121.36, 125.44 (2×), 127.64 (2×), 127.68 (2×), 129.54, 129.64, 132.79, 140.33, 141.87, 142.79, 143.43, 152.34, 164.10. **SC87508:** $[M+H]^+$ calcd. for $C_{27}H_{34}N_8O_5S_2$, 615.217; found for $[M+H]^+$: 615.2. ^1H NMR (DMSO-d_6): 11.63-11.44 (m, 2H), 8.41 (bs, 1H), 8.05-7.98 (m, 1H), 7.84 (d, J=8.3Hz, 1H), 7.79-7.66 (m, 5H), 7.50 (t, J=8.0Hz, 1H), 7.43 (d, J=8.4Hz, 2H), 7.25 (d, J=7.9Hz, 1H), 4.03 (bs, 2H), 3.59-3.47 (m, 2H), 3.00-2.80 (m, 4H), 2.68-2.39 (m, 6H). **SC87860:** $[M+H]^+$ calcd. for $C_{23}H_{26}N_6O_5S_2$, 531.148; found for $[M+H]^+$: 531.1. ^1H NMR (DMSO-d_6): 10.90 (s broad, 1H, 1NH); 10.85 (s broad, 1H, 1NH); 10.85 (s broad, 1H, 1NH); 9.14 (broad, 1H, NH); 8.05 (t broad, 1H, NH); 7.83 (m broad, 1H, Ar-H); 7.76-7.68 (m, 7H, Ar-H); 7.54-7.41 (m, 3H, Ar-H); 7.30 (s broad, 2H, NH_2); 7.16-7.11 (m, 1H, Ar-H); 4.03 (broad, 2H, CH_2); 3.11 (s broad, 6H, 2CH_3). **SC87866:** $[M+H]^+$ calcd. for $C_{28}H_{32}N_8O_5S_2$, 625.201; found for $[M+H]^+$: 625.1. ^1H NMR (DMSO-d_6): 11.40 (bm, 2H), 8.54 (bs, 1H), 8.03 (m, 1H), 7.85 (m, 1H), 7.79-7.65 (m, 6H), 7.51-7.38 (m, 3H), 7.30 (bs, 2H), 7.11 (d, J=7.4Hz, 1H), 4.02 (bm, 2H), 3.61-3.40 (m, 4H), 2.72-2.60 (m, 8H). **SC87867:** $[M+H]^+$ calcd. for $C_{29}H_{35}N_7O_5S_2$, 625.222; found for $[M+H]^+$: 625.1. ^1H NMR (DMSO-d_6): 11.52 (s, 1H, 1NH); 11.50 (s broad, 1H, 1NH); 8.03 (t broad, 1H, NH); 7.86 (m broad, 1H, Ar-H); 7.79-7.67 (m, 7H, Ar-H); 7.52-7.41 (m, 3H, Ar-H); 7.30 (s broad, 2H, NH_2); 7.14-7.09 (m, 1H, Ar-H); 4.03 (broad, 2H, CH_2); 3.57 (broad, 4H, 2CH_2); 2.60 (broad, 4H, 2CH_2); 2.26 (d, 2H, J=6.6 Hz, CH_2); 0.84 (m, 1H, CH); 0.47 (m, 2H, CH_2); 0.09 (m, 2H, CH_2). **SC87868:** $[M+H]^+$ calcd. for $C_{29}H_{35}N_7O_7S_2$, 658.211; found for $[M+H]^+$: 658.1.^1H NMR (DMSO-d_6): 11.53 (s, 1H, 1NH); 11.49 (s broad, 1H, 1NH); 8.02 (t broad, 1H, NH); 7.83 (m broad, 1H, Ar-H); 7.78-7.67 (m, 7H, Ar-H); 7.50-7.40 (m, 3H, Ar-H); 7.30 (s broad, 2H, NH_2); 7.11-7.06 (m, 1H, Ar-H); 4.93 (t, 1H, J=4.4 Hz, CH); 4.02 (broad, 2H, CH_2); 3.90-3.74 (m, 4H, 2CH_2); 3.51 (broad, 4H, 2CH_2); 2.65 (broad, 4H, 2CH_2); 2.57 (d, 2H, J=4.4 Hz, CH_2). **SC87869:** $[M+H]^+$ calcd. for $C_{30}H_{37}N_7O_6S_2$, 656.232; found for $[M+H]^+$: 656.1. ^1H NMR (DMSO-d_6): 11.38 (s, 1H, 1NH); 11.35 (s broad, 1H, 1NH); 8.03 (t broad, 1H, NH); 7.85 (m broad, 1H, Ar-H); 7.77-7.67 (m, 7H, Ar-H); 7.52-7.41 (m, 3H, Ar-H); 7.30 (s broad, 2H, NH_2); 7.13-7.09 (m, 1H, Ar-H); 4.02 (broad, 2H, CH_2); 3.98-3.90 (m, 1H, CH); 3.77-3.50 (group of signals, 6H, OCH_2+2pipCH_2); DMSO signal covers a group of signals; 1.98-1.41 (group of multiplets, 4H, 2CH_2). **SC87870:** $[M+H]^+$ calcd. for $C_{28}H_{35}N_7O_6S_2$, 630.217; found for $[M+H]^+$: 630.1. ^1H NMR (DMSO-d_6): 11.43 (s, 1H, 1NH); 11.40 (s broad, 1H, 1NH); 8.03 (t broad, 1H, NH); 7.85 (m broad, 1H, Ar-H); 7.78-7.67 (m, 7H, Ar-H); 7.52-7.41 (m, 3H, Ar-H); 7.30 (s broad, 2H, NH_2); 7.13-7.09 (m, 1H, Ar-H); 4.03 (broad, 2H, CH_2); 3.55 (broad, 4H, 2CH_2); 3.45 (t, 2H; J=5.6 Hz, OCH_2); 3.23 (s, 3H, CH_3); 2.62-2.53 (broad+q, 6H, 3CH_2). **SC88381:** $[M+H]^+$ calcd. for $C_{29}H_{38}N_8O_5S_2$, 643.248; found for $[M+H]^+$: 643.2. **SC88464:**

$[M+H]^+$ calcd. for $C_{29}H_{37}N_7O_6S_2$, 644.233; found for $[M+H]^+$: 644.2. **SC82686:** $[M+H]^+$ calcd. for $C_{28}H_{33}N_7O_7S_2$, 644.196; found for $[M+H]^+$: 644.1. ^{1}H NMR (DMSO-d_6): 9.61-9.21 (very broad, 4H, 4NH); 8.08 (t, 1H, NH); 7.89-7.87 (m, 1H, Ar-H); 7.77-7.52 (m, 8H, Ar-H); 7.45-7.41 (m, 2H, Ar-H); 7.28 ((broad, 2H, NH_2); 7.24-7.20 (m, 1H, Ar-H); 4.12-4.02 (m, 4H, CH_2+OCH_2); 3.82-3.40 (broad, 8H, 4CH_2); 1.20 (t, 3H, J=7.1 Hz, CH_3). ^{13}C NMR (75 MHz, DMSO-d_6): 14.38, 42.12, 45.45 (2×), 47.09 (2×), 60.97, 117.06, 117.45 (2×), 120.75, 121.09, 125.42 (2×), 127.55 (2×), 127.69 (2×), 129.48, 230.09, 132.32, 140.86, 141.90, 142.78, 152.77, 154.39, 164.63, 166.92. **SC82693:** $[M+H]^+$ calcd. for $C_{30}H_{37}N_7O_7S_2$, 672.227; found for $[M+H]^+$: 672.2. ^{1}H NMR (DMSO-d_6): 9.58 (s broad, 1H, NH); 9.52 (s broad, 1H, NH); 9.45 (broad, 1H, NH); 9.22 (s broad, 1H, NH); 8.08 (t, 1H, J=6.4 Hz, NH); 7.89 (m, 1H, Ar-H); 7.76-7.41 (m, 10H, Ar-H); 7.28 (s broad, 2H, NH_2); 7.23-7.20 (m, 1H, Ar-H); 4.03 (d, 2H, J=6.3 Hz, CH_2); 3.74 (m broad, 2H, CH_2); 3.63 (m broad, 2H, CH_2); water signal partially covers a group of signals; 1.42 (s, 9H, 3CH_3). **SC82694:** $[M+H]^+$ calcd. for $C_{31}H_{39}N_7O_7S_2$, 686.243; found for $[M+H]^+$: 686.2. **SC83287:** $[M+H]^+$ calcd. for $C_{27}H_{31}N_7O_6S_2$, 614.185; found for $[M+H]^+$: 614.1. **SC83288:** $[M+H]^+$ calcd. for $C_{27}H_{31}N_7O_7S_2$, 630.180 ; found for $[M+H]^+$: 630.1. ^{1}H NMR (300 MHz, DMSO-d_6): 9.96 (s, 1 H), 9.90 (s, 1 H), 9.39 (bs, 1 H), 8.08 (t, J = 6.3 Hz, 1 H), 7.86-7.89 (m, 1 H), 7.63-7.78 (m, 7 H), 7.54 (t, J = 7.9 Hz, 1 H), 7.44 (d, J = 8.3 Hz, 2 H), 7.29 (bs, 2 H), 7.21 (d, J = 7.5 Hz, 1 H), 4.03 (d, J = 6.1 Hz, 2 H), 3.64 (s, 3 H), 3.55-3.62 (m, 6 H), 3.28-3.32 (m, 2 H). ^{13}C NMR (75 MHz, DMSO-d_6): 42.23 (2×), 45.55 (3×), 52.58, 117.43, 117.74 (2×), 121.46, 121.60, 125.56 (2×), 127.80 (4×), 129.63, 129.81, 132.97, 140.37, 142.01, 142.91, 143.47, 152.41, 154.92, 164.39. element. anal.: element: (calc. comp. incl. add. (%) / found (%)): C: (48.68 / 48.25), Cl: (5.32 / 4.77), H: (4.84 / 5.6), N: (14.72 / 14.51), O: (16.81 / 18.1), S: (9.63 / 7.99). **SC83289:** $[M+H]^+$ calcd. for $C_{28}H_{33}N_7O_6S_2$, 628.201; found for $[M+H]^+$: 628.0. ^{1}H NMR (DMSO-d_6): 10.52 (very broad, 2H, 2NH); 9.38 (broad, 1H, NH); 8.05 (t broad, 1H, NH); 7.85 (broad, 1H, Ar-H); 7.76-7.66 (m, 7H, Ar-H); 7.54-7.41 (m, 3H, Ar-H); 7.29 (broad, 2H, NH_2); 7.19-7.14 (m, 1H, Ar-H); 4.03 (d broad, 2H, CH_2); 3.67-3.50 (broad, 8H, 4H_2); 2.35 (q, 2H, J=7.4 Hz, CH_2); 1.00 (t, 3H, J=7.4 Hz, CH_3). **SC83290:** $[M+H]^+$ calcd. for $C_{30}H_{37}N_7O_6S_2$, 656.232; found for $[M+H]^+$: 656.0. ^{1}H NMR (DMSO-d_6): 10.05-9.32 (very broad, 4H, 4NH); 8.07 (t, 1H, J=6.3 Hz, NH); 7.85 (broad, 1H, Ar-H); 7.76-7.62 (m, 7H, Ar-H); 7.57-7.51 (m, 1H, Ar-H);7.46-7.40 (m, 2H, Ar-H); 7.29 (broad, 2H, NH_2); 7.23-7.19 (m, 1H, Ar-H); 4.03 (d, 2H, J=5.9 Hz, CH_2); 3.69-3.52 (broad, 8H, 4H_2); 2.33 (t, 2H, J=7.4 Hz, CH_2); 1.49 (m, 2H, 7.4 Hz, CH_2); 1.30 (m, 2H, 7.4 Hz, CH_2); 0.88 (t, 3H, J=7.4 Hz, CH_3). **SC83311:** $[M+H]^+$ calcd. for $C_{29}H_{35}N_7O_7S_2$, 658.211; found for $[M+H]^+$: 658.1. ^{1}H NMR (DMSO-d_6): 11.06-10.71 (s broad, 2H, 2NH); 8.03 (t, 1H, J=6.1Hz, NH); 7.85-7.81 (m broad, 1H, Ar-H); 7.77-7.66 (m, 7H, Ar-H); 7.51-7.41 (m, 3H, Ar-H); 7.29 (broad, 2H, NH_2); 7.14-7.10 (m, 1H, Ar-H); 4.03 (d, 2H, J=6.0 Hz, CH_2); 3.98 (t, 2H, J=6.6 Hz, OCH_2); water signal covers a group of signals; 1.59 (m, 2H, CH_2); 0.89 (t, 3H, J=7.4 Hz, CH_3). **SC87410:** $[M+H]^+$ calcd. for $C_{34}H_{46}N_8O_7S_2$, 743.301; found for $[M+H]^+$: 743.3. ^{1}H NMR

(DMSO-d_6): 11.32-11.23 (m, 2H), 8.47 (s, 1H), 8.03 (t, J = 5.7 Hz, 1H), 7.85-7.80 (m, 1H), 7.79-7.66 (m, 7H), 7.52-7.39 (m, 3H), 7.30 (s, 2H), 7.09 (d, J = 7.6 Hz, 1H), 6.76 (s, 1H), 4.02 (d, J = 5.5 Hz, 2H), under water and DMSO peak (12H), 1.61-1.20 (m, 13H). **SC82683:** $[M+H]^+$ calcd. for $C_{27}H_{33}N_7O_5S_2$, 600.206; found for $[M+H]^+$: 600.2. **SC82684:** $[M+H]^+$ calcd. for $C_{30}H_{37}N_7O_5S_2$, 640.237; found for $[M+H]^+$: 640.2. **SC82689:** $[M+H]^+$ calcd. for $C_{27}H_{33}N_7O_5S_2$, 600.206; found for $[M+H]^+$: 600.2. ^{1}H NMR (DMSO-d_6): 9.80-9.55 (broad, 3H, 3NH), 9.04 (broad, 1H, NH); 8.08 (t, 1H, J=6.4 Hz, NH); 7.94 (m, 1H, Ar-H); 7.76-7.51 (m, 8H, Ar-H); 7.45-7.41 (m, 2H, Ar-H); 7.28 (broad, 2H, NH_2); 7.25-7.21 (m, 1H, Ar-H); 4.03 (d, 2H, J=6.2 Hz, CH_2); 3.84-3.54 (broad, 4H, $2CH_2$); water signal partially covers a group signals; 2.84-2.70 (broad, 6H, $2CH_3$); 2.37-2.01 (broad, 2H, CH_2). **SC82832:** $[M+H]^+$ calcd. for $C_{25}H_{28}N_6O_5S_2$, 557.164; found for $[M+H]^+$: 557.1. ^{1}H NMR (DMSO-d_6): 9.51 (s broad, 1H, NH); 9.42 (s broad, 1H, NH); 9.26 (broad, 1H, NH); 8.75 (s broad, 1H, NH); 8.08 (t, 1H, J=6.4 Hz, NH); 7.90 (m, 1H, Ar-H); 7.76-7.41 (m, 10H, Ar-H); 7.28 (s broad, 2H, NH_2); 7.25-7.21 (m, 1H, Ar-H); 4.04 (d, 2H, J=6.3 Hz, CH_2); 3.56 (t, 2H, J=6.9 Hz; CH_2); 3.41 (t, 2H, J=6.7 Hz; CH_2); 2.11-1.84 (m, 4H, $2CH_2$). **SC82869:** $[M+H]^+$ calcd. for $C_{28}H_{37}N_7O_5S_2$, 616.237; found for $[M+H]^+$: 616.2. ^{1}H NMR (DMSO-d_6): 9.87-9.17 (very broad, 4H, 4NH), 8.09 (t, 1H, J=6.3 Hz, NH); 7.96 (m, 1H, Ar-H); 7.77-7.55 (m, 8H, Ar-H); 7.45-7.41 (m, 2H, Ar-H); 7.28 (broad, 2H, NH_2); 7.25-7.21 (m, 1H, Ar-H); 4.04 (d, 2H, J=6.3 Hz, CH_2); water signal partially covers a group signals; 1.07 (m broad, 6H, $2CH_3$). **SC82870:** $[M+H]^+$ calcd. for $C_{27}H_{35}N_7O_5S_2$, 602.222; found for $[M+H]^+$: 602.2. **SC82871:** $[M+H]^+$ calcd. for $C_{26}H_{33}N_7O_5S_2$, 588.206; found for $[M+H]^+$: 588.2. ^{1}H NMR (DMSO-d_6): 9.90-9.59 (m, 2H), 9.15 (bs, 1H), 8.09 (t, J = 6.4 Hz, 1H), 7.93 (bs, 1H), 7.78-7.72 (m, 4H), 7.68 (d, J = 8.9 Hz, 2H), 7.64-7.53 (m, 2H), 7.43 (d, J = 8.3 Hz, 2H), 7.30-7.27 (m, 2H), 7.21 (d, J = 7.1 Hz, 1H), 4.04 (d, J = 6.3 Hz, 2H), 3.68-3.55 (m, 2H), 3.22 (s, 3H), 2.69-2.54 (m, 2H), under DMSO peak (6H). **SC82872:** $[M+H]^+$ calcd. for $C_{33}H_{48}N_8O_5S_2$, 701.326; found for $[M+H]^+$: 701.3. **SC82905:** $[M+H]^+$ calcd. for $C_{26}H_{31}N_7O_5S_2$, 586.190; found for $[M+H]^+$: 586.2. ^{1}H NMR (DMSO-d_6):9.61-9.46 (m, 2H), 9.19-9.07 (m, 1H), 8.02 (t, J = 6.4 Hz, 1H), 7.91-7.84 (m, 1H), 7.73-7.64 (m, 4H), 7.59 (d, J = 8.3 Hz, 2H), 7.49 (d, J = 4.8 Hz, 2H), 7.36 (d, J = 8.3 Hz, 2H), 7.22 (bs, 2H), 7.18-7.12 (m, 1H), 3.97 (d, J = 6.1 Hz, 2H), 3.92-3.81 (m, 2H), 3.71-3.56 (m, 4H), 3.47-3.35 (m, 2H), 2.00-1.80 (m, 3H). **SC77225:** $[M+H]^+$ calcd. for $C_{26}H_{30}N_6O_6S_2$, 587.174; found for $[M+H]^+$: 587.1. ^{1}H NMR (DMSO-d_6): 9.67 (s, 1H, NH); 9.61 (s, 1H, NH); 8.08 (t, 1H, J=6.3 Hz, NH); 7.86 (m, 1H, Ar-H); 7.76-7.51 (m, 8H, Ar-H); 7.45-7.42 (m, 2H, Ar-H); 7.28 (broad, 2H, NH_2); 7.21-7.18 (m, 1H, Ar-H); 4.95 (d broad, 1H, J=3.4 Hz, OH); 4.03 (d, 2H, J=6.2 Hz, CH_2); 3.90-3.83 (m broad, 1H, CH); water signal partially covers a group signals; 1.94-1.48 (broad, 4H, $2CH_2$). **SC81450:** $[M+H]^+$ calcd. for $C_{26}H_{28}N_6O_6S_2$, 585.159; found for $[M+H]^+$: 585.2. ^{1}H NMR (DMSO-d_6):9.69 (broad, 1H, NH); 9.62 (broad, 1H, NH); 8.08 (t, 1H, J=6.3 Hz, NH); 7.92 (m, 1H, Ar-H); 7.77-7.52 (m, 8H, Ar-H); 7.46-7.41 (m, 2H, Ar-H); 7.30-7.23 (m, 3H, Ar-H+NH_2); 4.03 (d, 2H, J=6.3 Hz, CH_2); 3.82 (broad, 4H, $2CH_2$); 2.64 (broad, 4H, $2CH_2$). **SC81452:**

$[M+H]^+$ calcd. for $C_{27}H_{33}N_7O_6S_2$, 616.201; found for $[M+H]^+$: 616.1. ^1H NMR (DMSO-d_6): 10.12 (broad, 1H, NH); 10.05 (broad, 1H, NH); 8.09 (t, 1H, J=6.2 Hz, NH); 7.79-7.62 (m, 8H, Ar-H); 7.53 (m, 1H, Ar-H); 7.47-7.41 (m, 2H, Ar-H); 7.29 (s broad, 2H, NH_2); 7.19-7.15 (m, 1H, Ar-H); 4.47 (t, 1H, J=5.3 Hz, OH); 4.03 (d, 2H, J=6.0 Hz, CH_2); 3.51 (m, 2H, OCH_2); water signal partially covers a group signals; 2.59 (broad, 4H, $2CH_2$). **SC81453:** $[M+H]^+$ calcd. for $C_{26}H_{31}N_7O_6S_2$, 602.185; found for $[M+H]^+$: 601.1. ^1H NMR (DMSO-d_6): 9.61 (broad, 1H, NH); 9.54 (broad, 1H, NH); 8.08 (t, 1H, J=6.2 Hz, NH); 7.83 (m, 1H, Ar-H); 7.79-7.41 (m, 11H, Ar-H); 7.28 (s broad, 2H, NH_2); 7.20-7.16 (m, 1H, Ar-H); 4.57 (t, 1H, J=5.1 Hz, OH); 4.03 (d, 2H, J=6.1 Hz, CH_2); water signal partially covers a group signals; 1.89-1.70 (broad, 3H, $CH+CH_2$); 1.40-1.20 (broad, 2H, CH_2). **SC81454:** $[M+H]^+$ calcd. for $C_{25}H_{30}N_6O_6S_2$, 575.174; found for $[M+H]^+$: 575.1. **SC81455:** $[M+H]^+$ calcd. for $C_{25}H_{28}N_6O_5S_3$, 589.136; found for $[M+H]^+$: 589.0. ^1H NMR (DMSO-d_6): 9.74 (broad, 1H, NH); 9.67 (broad, 1H, NH); 8.08 (t, 1H, J=6.3 Hz, NH); 7.87 (m, 1H, Ar-H); 7.77-7.51 (m, 8H, Ar-H); 7.46-7.41 (m, 2H, Ar-H); 7.28 (s broad, 2H, NH_2); 7.24-7.20 (m, 1H, Ar-H); 4.03 (d, 2H, J=6.2 Hz, CH_2); water signal partially covers a group signals; 2.85-2.80 (m, 4H, $2CH_2$). **SC81456:** $[M+H]^+$ calcd. for $C_{27}H_{34}N_6O_7S_2$, 619.201; found for $[M+H]^+$: 619.1. ^1H NMR (DMSO-d_6): 9.62 (broad, 1H, NH); 9.56 (broad, 1H, NH); 8.07 (t, 1H, J=6.3 Hz, NH); 7.79-7.41 (m, 13H, Ar-H); 7.28 (s broad, 2H, NH_2); 7.14-7.11 (m, 1H, Ar-H); 4.03 (d, 2H, J=6.2 Hz, CH_2); 3.82-3.48 (broad, 8H, $4CH_2$); water signal partially covers a group signals. **SC81457:** $[M+H]^+$ calcd. for $C_{27}H_{31}N_7O_6S_2$, 614.185; found for $[M+H]^+$: 614.1. ^1H NMR (DMSO-d_6): 9.63 (broad, 1H, NH); 9.56 (broad, 1H, NH); 8.08 (broad, 1H, NH); 7.84 (m, 1H, Ar-H); 7.77-7.42 (m, 10H, Ar-H); 7.32 (broad, 1H, NH_2); 7.28 (broad, 2H, NH_2); 7.21-7.18 (m, 1H, Ar-H); 6.87 (broad, 1H, NH_2); 4.04-4.02 (broad, 2H, CH_2); water signal partially covers a group signals; 1.92-1.62 (broad, 4H). **SC82687:** $[M+H]^+$ calcd. for $C_{31}H_{34}N_8O_5S_2$, 663.217; found for $[M+H]^+$: 663.2. ^1H NMR (DMSO-d_6): 9.63-9.52 (broad, 3H, 3NH), 9.25 (broad, 1H, NH); 8.67-8.65 (m, 2H, Pyr-H); 8.09 (t, 1H, J=6.1 Hz, NH); 7.88 (m, 1H, Ar-H); 7.77-7.52 (m, 10H, Ar-H+Pyr-H); 7.46-7.41 (m, 2H, Ar-H); 7.30 (broad, 2H, NH_2); 7.22-7.18 (m, 1H, Ar-H); 4.04 (d, 2H, J=6.2 Hz, CH_2); water signal partially covers a group signals; 2.80-2.57 (m, 4H, $2CH_2$). **SC82692:** $[M+H]^+$ calcd. for $C_{33}H_{48}N_8O_5S_2$, 701.326; found for $[M+H]^+$: 662.2. **SC85039:** $[M+H]^+$ calcd. for $C_{25}H_{28}N_6O_7S_3$, 621.126; found for $[M+H]^+$: 621.1.^1H NMR (DMSO-d_6): 9.88 (s, 1H), 9.60-9.27 (m, 2H), 8.08 (t, J = 6.3 Hz, 1H), 7.98 (bs, 1H), 7.81-7.70 (m, 4H), 7.67 (d, J = 8.6 Hz, 2H), 7.63-7.52 (m, 2H), 7.43 (d, J = 8.2 Hz, 2H), 7.34-7.25 (m, 3H), 4.04 (d, J = 6.3 Hz, 2H), 3.83-3.56 (m, 4H), 3.21-2.97 (m, 4H). **SC88408:** $[M+H]^+$ calcd. for $C_{20}H_{20}N_6O_6S_2$, 505.096; found for $[M+H]^+$: 506.1. ^1H NMR (DMSO-d_6): 9.74-9.48 (m, 1H), 9.29-9.08 (m, 1H), 9.03-8.88 (m, 1H), 8.00-7.56 (m, 8H), 7.55-7.41 (m, 1H), 7.37-7.15 (m, 6H), 5.72 (bs, 2H). **SC89127:** $[M+H]^+$ calcd. for $C_{27}H_{31}N_7O_8S_2$, 646.175; found for $[M+H]^+$: 646.2. **SC90436:** $[M+H]^+$ calcd. for $C_{29}H_{33}N_7O_8S_2$, 672.190; found for $[M+H]^+$: 672.2. **SC90478:** $[M+H]^+$ calcd. for $C_{34}H_{43}N_7O_8S_2$, 742.269; found for $[M+H]^+$: 756.3. ^1H NMR (DMSO-d_6): 10.87 (s, 1H), 9.19 (s,

1H), 9.03 (s, 1H), 8.07 (t, *J* = 6.3 Hz, 1H), 7.96 (t, *J* = 1.8 Hz, 1H), 7.78-7.62 (m, 8H), 7.56-7.49 (m, 1H), 7.47-7.39 (m, 4H), 7.28 (s, 2H), 4.07-3.97 (m, 2H), under water peak (8H), 2.75-2.62 (m, 2H), 1.65-1.47 (m, 2H), 1.39-1.20 (m, 8H), 0.92-0.80 (m, 3H). **SC83332 :**$[M+H]^+$ calcd. for $C_{29}H_{36}N_6O_5S_2$, 613.226; found for $[M+H]^+$: 613.2. **SC84185:** $[M+H]^+$ calcd. for $C_{30}H_{38}N_6O_5S_2$, 627.242; found for $[M+H]^+$: 627.2. **SC85194:** $[M+H]^+$ calcd. for $C_{27}H_{38}N_6O_4S$, 543.275; found for $[M+H]^+$: 543.3 **SC85219:** $[M+H]^+$ calcd. for $C_{29}H_{42}N_6O_4S$, 571.306; found for $[M+H]^+$: 585.3. **SC86755:** $[M+H]^+$ calcd. for $C_{22}H_{31}N_5O_4S_2$, 494.189; found for $[M+H]^+$: 494.2. **SC86756:** $[M+H]^+$ calcd. for $C_{22}H_{31}N_5O_4S_2$, 494.189; found for $[M+H]^+$: 494.2. **SC08323:** $[M+H]^+$ calcd. for $C_{20}H_{17}N_5O_5S$, 440.103; found for $[M+H]^+$: 440.1. **SC08324:** $[M+H]^+$ calcd. for $C_{20}H_{17}N_5O_5S$, 440.103; found for $[M+H]^+$: 440.1. **SC47789:** $[M+H]^+$ calcd. for $C_{24}H_{23}N_5O_6S$, 510.144; found for $[M+H]^+$: 510.1. **SC47790:** $[M+H]^+$ calcd. for $C_{24}H_{23}N_5O_5S$, 494.149; found for $[M+H]^+$: 494.0. ^1H NMR (DMSO-d_6): 10.09 (s, 1H, NH); 9.86 (s, 1H, NH); 9.25-8.65 (broad, 2H, NH_2); 8.31 (m, 2H, Ar-H); 8.10 (m, 2H, Ar-H); 7.85 (m, 2H, Ar-H); 7.73 (m, 1H, Ar-H); 7.63 (m, 2H, Ar-H); 7.52 (m, 1H, Ar-H); 7.43 (m, 1H, Ar-H); 7.14 (m, 1H, Ar-H); 3.46-3.28 (broad, 4H, $2NCH_2$) water signal partly covers this group of signals; 1.96-1.80 (broad, 4H, $2CH_2$). **SC47791:** $[M+H]^+$ calcd. for $C_{25}H_{26}N_6O_5S$, 523.176; found for $[M+H]^+$: 523.2 ^1H NMR (DMSO-d_6): 10.18 (broad, 1H, NH); 9.96 (broad, 1H, NH); 9.51 (broad, 1H, NH); 9.26 (broad, 1H, NH); 8.39 (m, 2H, Ar-H); 8.18 (m, 2H, Ar-H); 7.94 (m, 2H, Ar-H); 7.76 (m, 1H, Ar-H); 7.71 (m, 2H, Ar-H); 7.63 (m, 1H, Ar-H); 7.53 (m, 1H, Ar-H); 7.18 (m, 1H, Ar-H); 3.76-3.28 (broad, 4H, $2NCH_2$) water signal partly covers this group of signals; 2.55-2.38 (broad, 4H, $2NCH_2$) DMSO signal covers partly this group of signals; 2.23 (s, 3H, CH_3). **SC47793:** $[M+H]^+$ calcd. for $C_{25}H_{25}N_5O_6S$, 524.160; found for $[M+H]^+$: 524.1. ^1H NMR (DMSO-d_6):10.13 (s, 1H, NH); 9.91 (s, 1H, NH); 9.37-9.16 (broad, 2H, NH); 8.29 (AB, 4H, J=63.3 Hz, 8.9 Hz, Ar-H); 7.82 (AB, 4H, J=67.0 Hz, 8.9 Hz, Ar-H); 7.78 (s, 1H, Ar-H); 7.61 (m, 1H, Ar-H); 7.52 (t, 1H, J=7.9 Hz, Ar-H); 7.19 (m, 1H, Ar-H); 4.95 (d, 1H, J=3.6 Hz, OH); 3.87-3.83 (broad, 1H, CH); 3.60-3.10 (broad, 4H, $2NCH_2$) water signal partly covers this group of signals; 2.00-1.40 (broad, 4H, $2CH_2$). **SC47830:** $[M+H]^+$ calcd. for $C_{21}H_{17}N_3O_7S$, 456.086; found for $[M+H]^+$: 456.1. **SC47946:** $[M+H]^+$ calcd. for $C_{21}H_{20}N_4O_5S$, 441.123; found for $[M+H]^+$: 441.1. **SC48019:** $[M+H]^+$ calcd. for $C_{22}H_{19}N_5O_7S$, 498.108; found for $[M+H]^+$: 498.1. **SC48020:** $[M+H]^+$ calcd. for $C_{23}H_{21}N_5O_7S$, 512.123; found for $[M+H]^+$: 512.1. **SC48021:** $[M+H]^+$ calcd. for $C_{25}H_{25}N_5O_7S$, 540.155; found for $[M+H]^+$: 540.1. **SC48022:** $[M+H]^+$ calcd. for $C_{25}H_{25}N_5O_7S$, 540.155; found for $[M+H]^+$: 540.1. ^1H NMR (DMSO-d_6): 9.25 (bs, 1H), 9.11-8.94 (m, 3H), 8.39 (d, *J*=8.9Hz, 2H), 8.18 (d, *J*=8.9Hz, 2H), 8.06-8.05 (m, 1H), 7.92 (d, *J*=8.9Hz, 2H), 7.72 (d, *J*=8.9Hz, 1H), 7.65 (dd, *J*=1.3Hz, *J*=8.1Hz, 1H), 7.57 (d, *J*=7.9Hz, 1H), 7.40 (t, *J*=7.9Hz, 1H), 4.02 (t, *J*=6.6Hz, 2H), 1.64-1.55 (m, 2H), 1.43-1.30 (m, 2H), 0.91 (t, *J*=7.3Hz, 3H). **SC80504:** $[M+H]^+$ calcd. for $C_{25}H_{24}N_4O_7S$, 525.144; found for $[M+H]^+$: 525.1. ^1H NMR (DMSO-d_6): 9.34 (bs, 1H), 9.00 (bs, 1H), 8.39 (d, *J*=9.0Hz, 2H), 8.18 (d, *J*=9.0Hz, 2H), 7.92 (d, *J*=8.9Hz, 2H), 7.70 (d, *J*=9.0Hz, 2H), 7.55 (t, *J*=1.6Hz, 1H), 7.45-

7.40 (m, 1H), 7.35 (t, *J*=7.8Hz, 1H), 6.98 (td, *J*=1.3Hz, *J*=7.3Hz, 1H), 4.76 (d, *J*=4.1Hz, 1H), 4.08-3.04 (m, 4H) under water peak, 1.80-1.26 (m, 4H). **SC80548:** $[M+H]^+$ calcd. for $C_{25}H_{25}N_5O_6S$, 524.160; found for $[M+H]^+$: 524.2. ^1H NMR (DMSO-d_6): 9.74 (bs, 1H), 9.61 (bs, 1H), 9.25 (bs, 1H), 8.98 (bs, 1H), 8.39 (d, *J*=8.9Hz, 2H), 8.18 (d, *J*=8.9Hz, 2H), 7.94 (d, *J*=8.9Hz, 2H), 7.75-7.67 (m, 4H), 7.53 (d, *J*=8.7Hz, 2H), 4.94 (d, *J*=3.6Hz, 1H), 3.94-3.73 (m, 2H), 3.64-3.47 (m, 2H), 2.01-1.39 (m, 4H). **SC80548:** $[M+H]^+$ calcd. for $C_{25}H_{25}N_5O_6S$, 524.160; found for $[M+H]^+$: 524.2. **SC80613:** $[M+H]^+$ calcd. for $C_{20}H_{16}N_4O_6S$, 441.087; found for $[M+H]^+$: 441.1. ^1H NMR (DMSO-d_6): 9.34 (bs, 1H), 9.07 (bs, 1H), 8.39 (d, *J*=9.0Hz, 2H), 8.18 (d, *J*=9.0Hz, 2H), 7.93 (d, *J*=9.0Hz, 2H), 7.82 (d, *J*=8.8Hz, 2H), 7.71 (d, *J*=9.0Hz, 2H), 7.51 (d, *J*=8.8Hz, 2H), 7.16 (bs, 1H). **SC48443:** $[M+H]^+$ calcd. for $C_{20}H_{17}BrN_4O_3S$, 473.028; found for $[M+H]^+$: 473.0. ^1H NMR (DMSO-d_6): 10.04 (bm, 1H), 9.78 (bs, 1H), 9.22-8.61 (bm, 3H), 7.94-7.80 (m, 7H), 7.75-7.66 (m, 3H), 7.54 (t, *J*=8.0Hz, 1H), 7.37 (d, *J*=7.0Hz, 1H). **SC77215:** $[M+H]^+$ calcd. for $C_{25}H_{25}BrN_4O_4S$, 557.085; found for $[M+H]^+$: 556.6 ^1H NMR (DMSO-d_6): 9.59 (s, 1H, NH); 9.42 (s, 1H, NH); 9.30 (s, 1H, NH); 9.02 (s, 1H, NH); 7.82-7.43 (m, 11H, Ar-H); 7.13 (m, 1H, Ar-H); 4.88 (d, 1H, J=3.5 Hz, OH); 3.84-3.13 (m, 5H, CH+2CH_2) water signal partly covers this group of signals; 1.92-1.34 (m, 4H, 2CH_2). **SC80503:** $[M+H]^+$ calcd. for $C_{25}H_{25}ClN_4O_4S$, 513.136; found for $[M+H]^+$: 513.0. ^1H NMR (DMSO-d_6): 10.44 (s, 1H, NH); 9.33 (broad, 1H, NH); 9.26 (s, 1H, NH); 9.05 (broad, 1H, NH); 7.91-7.80 (m, 5H, Ar-H); 7.70-7.64 (m, 4H, Ar-H); 7.56-7.47 (m, 2H, Ar-H); 7.18-7.16 (m, 1H, Ar-H); 4.91 (d, 1H, J=3.6 Hz, OH); 3.86-3.79 (broad, 1H, CH); water signal covers the group of 2NCH_2 signals; 1.84-1.52 (broad, 4H, 2CH_2). **SC80549:** ^1H NMR (DMSO-d_6): 9.25 (bs, 1H), 8.93 (bs, 1H), 7.96-7.83 (m, 6H), 7.69-7.65 (m, 4H), 7.63-7.56 (m, 1H), 7.49 (d, *J*=7.9Hz, 1H), 7.35 (t, *J*=7.9Hz, 1H), 7.28 (bs, 1H). **SC80697:** $[M+H]^+$ calcd. for $C_{25}H_{25}BrN_4O_4S$, 557.085; found for $[M+H]^+$: 557.1. ^1H NMR (DMSO-d_6): 10.69 (bs, 1H), 10.58 (bs, 1H), 9.06 (bm, 1H), 7.88-7.80 (m, 6H), 7.77-7.65 (m, 4H), 7.51 (d, *J* = 8.8 Hz, 2H), 4.93 (bs, 1H), 3.34-3.88(m, 4H) under water peak, 1.50-1.94 (m, 4H). **SC81468:** $[M+H]^+$ calcd. for $C_{26}H_{27}BrN_4O_4S$, 571.101; found for $[M+H]^+$: 570.9. ^1H NMR (DMSO-d_6): 10.22 (broad, 1H, NH); 10.03 (broad, 1H, NH); 9.40 (broad, 2H, NH); 8.11-7.86 (m, 9H, Ar-H); 7.84-7.79 (m, 1H, Ar-H); 7.76-7.68 (m, 1H, Ar-H); 7.39-7.35 (m, 1H, Ar-H); 4.76 (t, 1H, J=5.0 Hz, OH); 4.07-4.02 (broad, 2H, CH_2); water signal partially covers the group of 2NCH_2 signals; 2.01-1.94 (broad, 3H, CH+CH_2); 1.50-1.40 (broad, 2H, CH_2). **SC81471:** $[M+H]^+$ calcd. for $C_{26}H_{29}BrN_4O_5S$, 589.112; found for $[M+H]^+$: 589.1. **SC81472:** $[M+H]^+$ calcd. for $C_{24}H_{23}BrN_4O_3S_2$, 559.047; found for $[M+H]^+$: 559.0. **SC81474:** $[M+H]^+$ calcd. for $C_{24}H_{23}BrN_4O_3S$, 527.075; found for $[M+H]^+$: 527.0. **SC81476:** $[M+H]^+$ calcd. for $C_{26}H_{26}BrN_5O_4S$, 584.096; found for $[M+H]^+$: 584.0. ^1H NMR (DMSO-d_6): 9.88 (bs, 1H), 9.65 (bs, 1H), 7.95-7.80 (m, 7H), 7.75-7.66 (m, 3H), 7.61-7.54 (m, 1H), 7.48 (t, *J* = 7.9 Hz, 1H), 7.30 (s, 1H), 7.13 (d, *J* = 7.6 Hz, 1H), 6.82 (bs, 1H), 3.90-3.78 (m, 1H), 3.20-2.90 (m, 4H), 1.85-1.46 (m, 4H). **SC81509:** $[M+H]^+$ calcd. for $C_{25}H_{27}N_5O_4S$, 494.186; found for $[M+H]^+$: 494.1. ^1H NMR (DMSO-d_6): 11.30 (s, 1H, NH); 11.22 (s, 1H, NH); 7.81 (m, 1H,

Ar-H); 7.72-7.66 (m, 5H, Ar-H); 7.52-7.41 (m, 3H, Ar-H); 7.10-7.06 (m, 1H, Ar-H); 6.62-6.57 (m, 2H, Ar-H); 6.05 (s broad, 2H, NH_2); 3.87-3.76 (m, 1H, CH); 3.75-3.67 (m, 2H, NCH_2); water signal partially covers a group signals; 1.90-1.78 (broad, 2H, CH_2); 1.58-1.44 (broad, 2H, CH_2). **SC08666:** $[M+H]^+$ calcd. for $C_{22}H_{23}N_5O_4S$, 454.155; found for $[M+H]^+$: 554.1. **SC48124:** $[M+H]^+$ calcd. for $C_{20}H_{19}N_5O_3S$, 410.128; found for $[M+H]^+$: 410.1. **SC48125:** $[M+H]^+$ calcd. for $C_{20}H_{19}N_5O_3S$, 410.128; found for $[M+H]^+$: 410.1. **SC48092:** $[M+H]^+$ calcd. for $C_{21}H_{21}N_5O_5S_2$, 488.106; found for $[M+H]^+$: 488.1. **SC48129:** $[M+H]^+$ calcd. for $C_{25}H_{24}N_6O_6S_2$, 569.127; found for $[M+H]^+$: 569.1. **SC81457:** $[M+H]^+$ calcd. for $C_{27}H_{31}N_7O_6S_2$, 614.185; found for $[M+H]^+$: 614.2. **SC84129:** $[M+H]^+$ calcd. for $C_{30}H_{37}N_7O_4S$, 592.270; found for $[M+H]^+$: 592.3. **SC84199:** $[M+H]^+$ calcd. for $C_{30}H_{39}N_7O_3S$, 578.291; found for $[M+H]^+$: 578.3. ^{1}H NMR (DMSO-*d$_6$*): 10.77 (bs, 1H), 10.40 (bs, 1H), 8.48 (s, 1H), 7.82 (bs, 1H), 7.68 (d, *J* = 8.2 Hz, 2H), 7.50-7.38 (m, 5H), 7.13 (t, *J* = 5.8 Hz, 1H), 7.09-6.98 (m, 3H), 6.61 (d, *J* = 8.7 Hz, 2H), 5.88 (s, 1H), 3.74-3.16 (m, 8H), 2.89-2.75 (m, 2H), 2.62-2.54 (m, 2H), 2.38-2.29 (m, 2H), 1.48-1.21 (m, 4H), 0.88 (t, *J* = 7.2 Hz, 3H). **SC84302:** $[M+H]^+$ calcd. for $C_{30}H_{37}N_7O_4S$, 592.270; found for $[M+H]^+$: 592.3. **SC84472:** $[M+H]^+$ calcd. for $C_{30}H_{39}N_7O_3S$, 578.291; found for $[M+H]^+$: 578.3. ^{1}H NMR (DMSO-*d$_6$*): 9.71-9.45 (m, 2H), 8.91 (s, 1H), 7.83-7.71 (m, 3H), 7.59 (d, *J* = 8.4 Hz, 1H), 7.52-7.42 (m, 3H), 7.26 (s, 2H), 7.17 (d, *J* = 8.8 Hz, 2H), 7.10 (d, *J* = 7.5 Hz, 1H), 6.56 (d, *J* = 8.8 Hz, 2H), 3.82 (bs, 1H), 3.48-3.16 (m, 6H), 3.10-3.02 (m, 2H), 2.95-2.86 (m, 2H), 2.75-2.58 (m, 4H), 1.53-1.19 (m, 4H), 0.88 (t, *J* = 7.3, Hz, 3H). **SC84509:** $[M+H]^+$ calcd. for $C_{28}H_{36}N_8O_5S_2$, 629.233; found for $[M+H]^+$: 629.1. ^{1}H NMR (DMSO-*d$_6$*): 11.39-11.25 (m, 2H), 8.19 (s, 1H), 7.87-7.67 (m, 7H), 7.62-7.51 (m, 4H), 7.46 (t, *J* = 7.9 Hz, 1H), 7.31 (bs, 1H), 7.08 (d, *J* = 7.3 Hz, 1H), 6.97 (s, 1H), 6.86 (d, *J* = 8.9 Hz, 1H), under water and DMSO peak (10H), 1.48-1.20 (m, 4H), 0.88 (t, *J* = 7.22, 7.22 Hz, 3H). **SC84952:** $[M+H]^+$ calcd. for $C_{28}H_{35}N_7O_3S$, 550.260; found for $[M+H]^+$: 550.3. **SC84969:** $[M+H]^+$ calcd. for $C_{29}H_{37}N_7O_3S$, 564.276; found for $[M+H]^+$: 564.3. ^{1}H NMR (DMSO-*d$_6$*): 9.98 (s, 1H), 9.38 (s, 1H), 8.44 (s, 1H), 7.80-7.73 (m, 3H), 7.58 (d, *J* = 8.2 Hz, 1H), 7.52 (d, *J* = 8.4 Hz, 2H), 7.41 (t, *J* = 7.9 Hz, 1H), 7.24 (bs, 2H), 7.15 (d, *J* = 8.9 Hz, 2H), 7.01 (d, *J* = 7.6 Hz, 1H), 6.49 (d, *J* = 8.9 Hz, 2H), 6.07 (t, *J* = 6.0 Hz, 1H), 4.32 (d, *J* = 5.2 Hz, 2H), under water and DMSO peak (10H), 1.47-1.21 (m, 4H), 0.88 (t, *J* = 7.23, 7.23 Hz, 3H). **SC85955:** $[M+H]^+$ calcd. for $C_{26}H_{29}N_7O_7S_2$, 616.164; found for $[M+H]^+$: 616.2. ^{1}H NMR (DMSO-*d$_6$*):11.04 (d, *J* = 16.5 Hz, 2H), 8.48 (s, 1H), 7.84-7.81 (m, 1H), 7.75-7.59 (m, 7H), 7.53-7.41 (m, 2H), 7.25-7.05 (m, 5H), under water peak (8H), 3.63 (s, 3H). **SC86099:** $[M+H]^+$ calcd. for $C_{28}H_{35}N_7O_5S_2$, 614.222; found for $[M+H]^+$: 613.2. ^{1}H NMR (DMSO-*d$_6$*):10.53 (bs, 1H), 10.20 (bs, 1H), 8.36 (s, 1H), 8.21 (t, *J* = 1.7 Hz, 1H), 8.05-7.99 (m, 1H), 7.90-7.84 (m, 1H), 7.82-7.78 (m, 1H), 7.74 (t, *J* = 7.81 Hz, 1H), 7.65 (d, *J* = 8.6 Hz, 1H), 7.60-7.51 (m, 2H), 7.46 (t, *J* = 7.9 Hz, 1H), 7.38 (d, *J* = 8.9 Hz, 2H), 7.07 (d, *J* = 7.6 Hz, 1H), 6.97 (d, *J* = 8.9 Hz, 2H), under water and DMSO peak (10H), 1.49-1.20 (m, 4H), 0.88 (t, *J* = 7.23, 7.23 Hz, 3H). **SC86100:** $[M+H]^+$ calcd. for $C_{28}H_{35}N_7O_5S_2$, 613.222; found for $[M+H]^+$: 613.2. ^{1}H NMR (DMSO-*d$_6$*):10.25 (bs,

1H), 9.95 (bs, 1H), 8.36 (s, 1H), 7.96 (d, *J* = 8.5 Hz, 2H), 7.88 (d, *J* = 8.60 Hz, 2H), 7.78 (s, 1H), 7.67-7.42 (m, 4H), 7.36 (d, *J* = 8.4 Hz, 2H), 7.09 (d, *J* = 7.6 Hz, 1H), 6.98 (d, *J* = 8.8 Hz, 2H), under water and DMSO peak (10H), 1.48-1.22 (m, 4H), 0.88 (t, *J* = 7.22, 7.22 Hz, 3H). **SC86123:** $[M+H]^+$ calcd. for $C_{29}H_{37}N_7O_5S_2$, 628.237; found for $[M+H]^+$: 628.2. ^{1}H NMR (DMSO-d_6):10.51 (bs, 1H), 10.17 (bs, 1H), 8.39 (s, 1H), 8.36-8.23 (m, 2H), 8.08-8.01 (m, 1H), 8.01-7.94 (m, 1H), 7.86-7.81 (m, 1H), 7.77 (t, *J* = 7.8 Hz, 1H), 7.72-7.53 (m, 3H), 7.52-7.39 (m, 3H), 7.19-7.05 (m, 3H), 3.96 (s, 2H), under water and DMSO peak (10H), 1.50-1.19 (m, 4H), 0.88 (t, *J* = 7.23, 7.23 Hz, 3H). **SC86124:** $[M+H]^+$ calcd. for $C_{29}H_{37}N_7O_5S_2$, 628.237; found for $[M+H]^+$: 628.2. ^{1}H NMR (DMSO-d_6): 10.22 (bs, 1H), 9.94 (bs, 1H), 8.40-8.19 (m, 2H), 8.05-7.91 (m, 4H), 7.85-7.79 (m, 1H), 7.71-7.53 (m, 2H), 7.48 (t, *J* = 7.9 Hz, 1H), 7.41 (d, *J* = 8.4 Hz, 2H), 7.18-7.05 (m, 3H), 3.96 (s, 2H), under water and DMSO peak (10H), 1.54-1.17 (m, 4H), 0.88 (t, *J* = 7.23, 7.23 Hz, 3H). **SC88165:** $[M+H]^+$ calcd. for $C_{31}H_{37}N_7O_4S$, 604.270; found for $[M+H]^+$: 604.3. ^{1}H NMR (DMSO-d_6): 9.84 (s, 1H), 9.71 (s, 1H), 9.47-8.90 (bm, 3H), 7.99-7.92 (m, 2H), 7.75-7.71 (m, 3H), 7.65 (t, *J*=8.9Hz, 2H), 7.54 (t, *J*=7.9Hz, 1H), 7.38 (d, *J*=7.7Hz, 1H), 7.31-7.20 (m, 5H), 3.96 (s, 2H). **SC88166:** $[M+H]^+$ calcd. for $C_{30}H_{37}N_7O_5S_2$, 640.237; found for $[M+H]^+$: 640.2. **SC48590:** $[M+H]^+$ calcd. for $C_{18}H_{21}N_5O_4S$, 404.139; found for $[M+H]^+$: 404.01. ^{1}H NMR (DMSO-d_6): 9.74-9.30 (m, 3H), 9.03-8.89 (m, 2H), 7.99 (bs, 1H), 7.77-7.71 (m, 3H), 7.67 (d, *J*=8.9Hz, 2H), 7.55 (t, *J*=8.0Hz, 1H), 7.39 (d, *J*=7.6Hz, 1H), 3.67-3.62 (m, 4H), 2.87-2.84 (m, 4H). **SC50582:** $[M+H]^+$ calcd. for $C_{28}H_{32}N_6O_4S$, 549.228; found for $[M+H]^+$: 549.2. **SC50603:** $[M+H]^+$ calcd. for $C_{29}H_{32}FN_5O_4S$, 566.223; found for $[M+H]^+$: 566.2. **SC50604:** $[M+H]^+$ calcd. for $C_{29}H_{33}FN_6O_3S$, 565.239; found for $[M+H]^+$: 565.2. **SC50633:** $[M+H]^+$ calcd. for $C_{28}H_{31}N_5O_5S$, 550.212; found for $[M+H]^+$: 550.2. **SC77227:** $[M+H]^+$ calcd. for $C_{26}H_{26}F_3N_5O_4S$, 562.173; found for $[M+H]^+$: 562.2. ^{1}H NMR (DMSO-d_6): 9.64-9.10 (m, 3H), 8.10 (m, 1H), 7.87 (bs, 1H), 7.73-7.51 (m, 6H), 7.47-7.33 (m, 1H), 7.11-6.99 (m, 1H), 4.97 (m, 1H), 4.03 (d, *J*=5.5Hz, 2H), 3.29 (m, 4H) under water peak, 2.00-1.27 (m, 4H). **SC80166:** $[M+H]^+$ calcd. for $C_{27}H_{28}F_3N_5O_4S$, 576.189; found for $[M+H]^+$: 576.2. ^{1}H NMR (DMSO-d_6): 9.86 (bs, 1H), 9.71 (bs, 1H), 9.40 (bs, 1H), 9.13 (bs, 1H), 7.82 (m, 1H), 7.76-7.70 (m, 4H), 7.64-7.61 (m, 1H), 7.58-7.45 (m, 2H), 7.21-7.12 (m, 2H), 4.96 (bs, 1H), 4.20 (bs, 2H), 3.90-3.77 (m, 2H), 3.63-3.45 (m, 2H), 3.30 (m, 3H) under water peak, 1.99-1.48 (m, 4H). **SC80502:** $[M+H]^+$ calcd. for $C_{20}H_{17}ClN_4O_3S$, 429.078; found for $[M+H]^+$: 429.1. **SC85370:** $[M+H]^+$ calcd. for $C_{30}H_{38}N_6O_4S$, 579.275; found for $[M+H]^+$: 579.3. **SC85371:** $[M+H]^+$ calcd. for $C_{30}H_{37}N_7O_4S$, 592.270; found for $[M+H]^+$: 592.3. **SC86525:** $[M+H]^+$ calcd. for $C_{23}H_{32}N_6O_3S$, 473.233; found for $[M+H]^+$: 473.2. **SC87510:** $[M+H]^+$ calcd. for $C_{24}H_{26}N_6O_6S_2$, 559.143; found for $[M+H]^+$: 559.1. ^{1}H NMR (DMSO-d_6): 10.69-9.10 (broad, 3H, 3NH); 8.44 (broad, 1H, NH); 7.81 (m, 1H, Ar-H); 7.75-7.59 (m, 7H, Ar-H); 7.49 (m, 1H, Ar-H); 7.22-7.08 (m, 5H, Ar-H+NH_2); 3.73 (broad, 4H, $2CH_2$); 3.54 (broad, 4H, $2CH_2$); water signal interferes with these two signals. **SC87512:** $[M+H]^+$ calcd. for $C_{25}H_{29}N_7O_5S_2$, 572.175; found for $[M+H]^+$: 572.1. ^{1}H NMR (DMSO-d_6): 10.45 (broad, 1H, NH);

10.34 (broad, 1H, NH); 8.21 (s, 1H, NH); 7.77-7.60 (m, 8H, Ar-H); 7.51 (m, 1H, Ar-H); 7.27-7.21 (m, 2H, Ar-H); 7.19-7.12 (m, 3H, Ar-H+NH_2); water signal partially covers a group signals; 2.23 (s, 3H, CH_3). **SC87513:** $[M+H]^+$ calcd. for $C_{26}H_{32}N_8O_5S_2$, 601.201; found for $[M+H]^+$: 601.2. ^1H NMR (DMSO-d_6): 11.69 (bs, 1H), 11.63 (bs, 1H), 8.42 (s, 2H), 8.03-7.97 (m, 1H), 7.82 (d, J = 8.2 Hz, 1H), 7.75-7.68 (m, 4H), 7.63 (d, J = 8.8 Hz, 2H), 7.48 (t, J = 8.0 Hz, 1H), 7.28-7.15 (m, 4H), 3.51 (t, J = 5.86, 5.86 Hz, 2H), under water and DMSO peak (11H). **SC81373:** $[M+H]^+$ calcd. for $C_{20}H_{17}BrN_4OS$, 441.038; found for $[M+H]^+$: 441. ^1H NMR (DMSO-d_6): 9.40 (bs, 2H), 9.32 (bs, 2H), 8.94 (bs, 2H), 7.95 (t, J = 1.8 Hz, 1H), 7.70-7.73 (m, 1H), 7.61-7.47 (m, 5H), 7.42 (d, J = 8.7 Hz, 2H), 7.36 (d, J = 8.4 Hz, 1H), 7.10 (d, J = 8.7 Hz, 2H). **SC82748:** $[M+H]^+$ calcd. for $C_{23}H_{28}F_3N_5O$, 448.232; found for $[M+H]^+$: 448.2. ^1H NMR (DMSO-d_6): 9.85-9.21 (m, 4H), 8.08 (bs, 1H), 7.90 (bs, 1H), 7.67-7.48 (m, 4H), 7.33 (d, J = 7.6 Hz, 1H), 7.19 (d, J = 7.5 Hz, 1H), under DMSO and water peak (9H), 1.58-1.40 (m, 2H), 1.38-1.21 (m, 2H), 0.89 (t, J = 7.3 Hz, 3H). **SC88407:** $[M+H]^+$ calcd. for $C_{21}H_{21}N_5O_4S$, 440.139; found for $[M+H]^+$: 440.1. **SC90612:** $[M+H]^+$ calcd. for $C_{25}H_{26}N_6O_7S_2$, 587.138; found for $[M+H]^+$: 587.1. ^1H NMR (DMSO-d_6): 9.19 (s, 1H), 8.94 (s, 1H), 8.15-8.01 (m, 2H), 7.78-7.69 (m, 4H), 7.65 (d, J = 9.0 Hz, 2H), 7.59-7.47 (m, 2H), 7.44 (d, J = 8.5 Hz, 2H), 7.29 (bs, 2H), 4.04 (d, J = 6.6 Hz, 2H), 3.85-3.80 (m, 4H), 3.49-3.41 (m, 1H). **SC12215:** $[M+H]^+$ calcd. for $C_{13}H_{15}N_3O_4S_2$, 342.058; found for $[M+H]^+$: 342.1. ^1H NMR (DMSO-d_6): 7.76-7.69 (m, 3H), 7.47-7.42 (m, 4H), 7.28 (bs, 2H), 6.23 (d, J=8.7Hz, 2H), 5.91 (bs, 2H), 3.96 (d, J=6.4Hz, 2H). **SC83244:** $[M+H]^+$ calcd. for $C_{15}H_{17}N_3O_5S_2$, 384.068; found for $[M+H]^+$: 384.1. ^1H NMR (DMSO-d_6): 10.28 (s, 1H), 8.08 (t, J = 6.3 Hz, 1H), 7.80-7.70 (m, 6H), 7.42 (d, J = 8.3 Hz, 2H), 7.28 (bs, 2H), 4.02 (d, J = 6.3 Hz, 2H), 2.09 (s, 3H). **SC86438:** $[M+H]^+$ calcd. for $C_{14}H_{16}N_4O_4S_3$, 401.041; found for $[M+H]^+$: 401.0. ^1H NMR (DMSO-d_6): 10.09 (s, 1H), 8.13 (t, J = 6.3 Hz, 1H), 7.81-7.70 (m, 7H), 7.45 (d, J = 8.4 Hz, 2H), 7.29 (bs, 2H), 4.04 (d, J = 6.3 Hz, 2H).

Supplementary References

1. Fox, J. G. *et al. The Mouse in Biomedical Research: Normative Biology, Husbandry, and Models*. Elsevier Science (2006).

2. Abdullah, A. S. & Baggot, J. D. Pharmacokinetics of imidocarb in normal dogs and goats. *J Vet Pharmacol Ther* **6**, 195-199 (1983).

3. Omasits, U., Ahrens, C. H., Muller, S. & Wollscheid, B. Protter: interactive protein feature visualization and integration with experimental proteomic data. *Bioinformatics* **30**, 884-886 (2014).
